# Identification of RNA-binding proteins in exosomes capable of interacting with different types of RNA: RBP-facilitated transport of RNAs into exosomes

**DOI:** 10.1371/journal.pone.0195969

**Published:** 2018-04-24

**Authors:** Luisa Statello, Marco Maugeri, Elena Garre, Muhammad Nawaz, Jessica Wahlgren, Alexandros Papadimitriou, Christina Lundqvist, Lennart Lindfors, Anna Collén, Per Sunnerhagen, Marco Ragusa, Michele Purrello, Cinzia Di Pietro, Natalie Tigue, Hadi Valadi

**Affiliations:** 1 Department of Rheumatology and Inflammation Research, Sahlgrenska Academy, University of Gothenburg, Guldhedsgatan, Gothenburg, Sweden; 2 Department of Chemistry and Molecular Biology, University of Gothenburg, Gothenburg, Sweden; 3 Department of Pharmaceutical Sciences, Innovative Medicines, AstraZeneca AB, Gothenburg, Sweden; 4 Department of Chemistry and Molecular Biology, Lundberg Laboratory, University of Gothenburg, Gothenburg, Sweden; 5 Department of Biomedical and Biotechnological Sciences—Section of Biology and Genetics G Sichel—BioMolecular, Genome and Complex Systems BioMedicine Unit (BMGS)—University of Catania, Catania, Italy; 6 Department of Antibody Discovery and Protein Engineering, MedImmune Ltd, Granta Park, Cambridge; University of Surrey, UNITED KINGDOM

## Abstract

The RNA that is packaged into exosomes is termed as exosomal-shuttle RNA (esRNA); however, the players, which take this subset of RNA (esRNA) into exosomes, remain largely unknown. We hypothesized that RNA binding proteins (RBPs) could serve as key players in this mechanism, by making complexes with RNAs and transporting them into exosomes during the biosynthesis of exosomes. Here, we demonstrate the presence of 30 RBPs in exosomes that were shown to form RNA–RBP complexes with both cellular RNA and exosomal-RNA species. To assess the involvement of these RBPs in RNA-transfer into exosomes, the gene transcripts encoding six of the proteins identified in exosomes (HSP90AB1, XPO5, hnRNPH1, hnRNPM, hnRNPA2B1, and MVP) were silenced by siRNA and subsequent effect on esRNA was assessed. A significant reduction of total esRNA was observed by post-transcriptional silencing of MVP, compared to other RBPs. Furthermore, to confirm the binding of MVP with esRNA, a biotinylated-MVP was transiently expressed in HEK293F cells. Higher levels of esRNA were recovered from MVP that was eluted from exosomes of transfected cells, as compared to those of non-transfected cells. Our data indicate that these RBPs could end up in exosomes together with RNA molecules in the form of RNA–ribonucleoprotein complexes, which could be important for the transport of RNAs into exosomes and the maintenance of RNAs inside exosomes. This type of maintenance may favor the shuttling of RNAs from exosomes to recipient cells in the form of stable complexes.

## Introduction

Extracellular vesicles (EVs) represent a heterogeneous population of nano- and micro-sized vesicles secreted by virtually all cell types studied so far–ranging from prokaryotes to eukaryotes. Among these, the best characterized are exosomes which are 30–150 nm in diameter, originate from the endocytic compartments and are matured in the multivesicular bodies (MVBs) of the late endosomes (for detailed mechanisms see [1, 2]). Upon maturation, the MVBs are fused with the plasma membrane and exosomes are then released into the extracellular environment, where they can subsequently be taken up by other cells. During the course of their biogenesis, the lumen of exosomes is filled with a subset of bioactive molecules, including, DNA and proteins, coding and non-coding RNAs (ncRNA) [3], from their originating (parental) cells. As this exosomal “cargo” can be delivered to other cells, exosomes are thought to be central mediators of intercellular communication [4–8], and can modulate physiological responses by mediating short and long distance inter-organ communication [9–11], and the progression of various diseases [10–15]. Their unique content makes them an ideal source for disease biomarkers [2], with the implementation of high throughput technologies [16].

Notably, we for the first time, and later on other researchers have shown that exosomes obtained from various sources contain a substantial amount of RNA [17–24], and that this exosomal RNA is intact and functional in recipient cells [3, 25–27]. In 2007, we reported that microRNAs (miRNAs) and mRNAs are transferred between cells as novel mechanism of genetic exchange between cells and was termed as exosomal shuttle RNA (esRNA) [17]. Unlike cells, exosomes contain little or no ribosomal RNA (rRNA), *i*.*e*., 18S and 28S rRNA [17, 19, 28], and the complement of RNA found in exosomes differs greatly from that detected in the cytoplasm of their donor cells [17, 28–32]. These differences suggest that RNA is not indiscriminately packaged into exosomes during their biosynthesis; rather a tightly regulated sorting and packaging mechanism may exist that provides exosomes with a unique subset of RNA [27, 32, 33]. However, the mechanisms by which esRNA is packaged into exosomes, remain largely unknown.

It is important to note that almost all RNA in cells exists as ribonucleoprotein (RNP) complexes. As such, proteins capable of interacting with RNA (i.e. RNA-binding proteins, or RBPs) are key players in the regulation of the post-transcriptional processing and transport of RNA molecules. Through specific interactions with their cognate RNA molecules, RBPs regulate RNA processing, nucleocytoplasmic transport and maturation, intra-compartmental localization and turnover [34–36]. In fact, RBPs can bind to both single-stranded as well as double-stranded RNA; demonstrating an ability to interact not only with mRNAs but also with small ncRNAs which may form RNP complexes. Such a compendium of interactions can be long lasting and is considered driving factor in several aspects of cellular metabolism.

Not only within the cell, but also in the extracellular space the presence of RNA in association with RBPs is increasingly being recognized. This implies the secretion of RNA in EVs as EV-associated forms [17, 27, 37], or secretion of RNA free of EVs as non-EV forms such as those coupled with Argonaute2 (Ago2) and high-density lipoprotein complexes [38–41]. These both forms are thought to help the maintenance of RNA in extracellular environment or extracellular transport. However, currently there is no consensus regarding the respective mechanisms, by which RBPs contribute the precise regulation of selective release of RNAs from cells either as EV free secreted RNAs or as EV-associated forms (i.e. esRNA). There is initial evidence that the sorting of selective miRNA species occurs across a range of cell types [42, 43]. This includes the participation of RBPs in the sorting of miRNAs into exosomes through recognizing specific miRNA motifs [44–46], and the abundance of free RNA in the cytoplasm, intra-cellular compartmental localization of RNA species or other mechanisms of active and passive sorting [27, 47–51]. The mechanisms through which such protein-RNA complexes are recognized by MVBs and RNA species are maintained in exosomes, however, remain elusive.

In this study, we sought to identify whether the RBPs extracted from exosomes could interact with RNAs extracted from different sources i.e. with exosomal RNA (esRNA), as well as cellular mRNA and cellular miRNA and their interactions can be detected in the extracellular environment. We hypothesized that these proteins could be involved in (a) the packaging of RNA into exosomes, (b) maintenance of RNA molecules inside exosomes when secreted to extracellular environment. We present a method (RNA electrophoretic mobility shift assay, REMSA) showing the binding of exosomal proteins to cellular RNA (miRNA, mRNA) and esRNA. Further to this, we developed a method for the purification of RNA-RBP complexes from exosomes for the identification of RBPs in exosomes. The extracted exosomal proteins were allowed to bind with biotinylated RNAs from cells and exosomes with added streptavidin-coated Dynabeads. The bound proteins were re-extracted and were then loaded onto an SDS-PAGE gel, and RNA-RBP bands were excised, trypsinised and analyzed using LC-MS/MS allowing us the identification of 30 RBPs in exosomes that bind to different RNA species (esRNA, cellular mRNA and cellular miRNA). Further, we validated the effect of RPBs on esRNA by silencing some of these RBPs in the cytoplasm and observed their consequence on RNA amount in exosomes. The post-transcriptional gene silencing of transcripts encoding six of the identified proteins (HSP90AB1, XPO5, hnRNPH1, hnRNPM, hnRNPA2B1, and MVP) show that down-regulation of RBPs (in particular Major Vault Protein (MVP)) caused a significant reduction of the total RNA present in exosomes. Furthermore, to validate the binding of MVP with esRNA, cells were transfected with a plasmid encoding the full-length human MVP. After transiently expressing the biotinylated-MVP in transfected cells, the exosomes were isolated and the biotinylated MVP was eluted/purified from exosomes of transfected cells to assess the MVP binding with esRNA. Results showed that the biotinylated-MVP was detected in transfected cells and was partitioned to exosomes. Moreover, the higher levels of esRNA were recovered from the MVP eluted from exosomes of transfected cells as compared to non-transfected cells. In light of our findings, we propose (a) these RBPs could end up in exosomes together with exosomal-RNA (esRNA) molecules in the form of complex of RNA and ribonucleoproteins (RNP-complexes), and (b) these exosomal-RBPs could be important for the fate of the exosomal RNAs, including the transport of RNAs into exosomes and the maintenance of RNAs inside exosomes. This type of maintenance and stability may favor the shuttling of RNAs from exosomes to recipient cells (so-called exosomal-shuttle RNA; esRNA), when cells are required to package RNA into exosomes and deliver to other cells.

## Materials and methods

### Cell culture and isolation of exosomes

Human epithelial (HTB-177) cells (ATCC, USA) were cultured in 175 cm^2^ culture flasks (Sarstedt, Sweden) according to the manufacturer’s instructions. The fetal bovine serum (Sigma-Aldrich, Sweden) was depleted of exosomes by centrifugation for 70 minutes at 120000*g* followed by filtration through 0.2 μm filters and was added to culture medium. Cells were cultured for 72 hr and were harvested at 80% confluency. Cells were trypsinized and pelleted at 300g for 5 minutes and cell pellets were collected for cell lysate analysis. Exosomes were isolated from culture supernatant as described previously [47], with slight modifications. Briefly, culture supernatant was centrifuged at 300g for 15 minutes to pellet the cellular debris. The supernatant was further centrifugation at 16500g for 30 minutes followed by filtration through a 0.2 μm filter. Finally, the supernatant was ultracentrifuged at 120000*g* on a Beckman Optima LE80K ultracentrifuge by using Ti70 rotor for 70 minutes. Exosome pellets were resuspended in 300 μl PBS or directly lysed for RNA isolation.

### RNA extraction and analysis

The short RNA fractions, composed of RNAs smaller than 200 nucleotides and enriched in miRNAs from HTB-177 cells, were isolated using miRNeasy Mini kit followed by miRNeasy Cleanup Kit (Qiagen, Hilden, Germany), according to the manufacturer’s instructions. MRNA from HTB-177 cells was extracted from total RNA using the Oligotex mRNA Mini Kit (Qiagen, Hilden, Germany). Total RNA from HTB-177 exosomes was isolated by Trizol reagent (Life Technologies, Carlsbad, California), following the manufacturer’s instructions. The pattern of RNA (yield, quality, and size) was evaluated using the Agilent RNA 6000 Nano Kit on an Agilent 2100 Bioanalyzer (Agilent Technologies).

### Isolation of native proteins

The native proteins from cells and exosomes were extracted using ProteoJET Mammalian Cell Lysis Reagent (Fermentas, Sweden) together with Protease Inhibitor Cocktail (Calbiochem) according to manufacturer’s instructions.

### Biotinylation of cellular and exosomal RNA

The biotinylation of cellular miRNA, cellular mRNA, and exosomal total RNA was performed using Pierce RNA 3' End Biotinylation Kit (Pierce, Thermo Fisher Scientific, USA) following the manufacturer’s instructions with some modifications. For each reaction cellular miRNA (50 pmol), cellular mRNA (1.6 pmol), RNA template control (50 pmol) and exosomal RNA (10 to 20 pmol) were biotinylated. Before incubating the reactions, the cellular miRNA/mRNA and total exosomal RNA were heated at 85°C for 5 minutes to relax the RNA secondary structure. Biotinylation reactions were incubated at 16°C for 2h (cellular miRNA), overnight (cellular mRNA), and 3h (exosomal total RNA), and determination of the biotinylation efficiency was performed according to the manufacturer´s protocol. Briefly, serial dilutions of the biotinylated cellular short RNA and total exosomal RNA were dotted onto a nylon membrane (Hybond-N+, Amersham Biosciences) using a biotinylated IRE (Iron Response Element) RNA control. The nylon membrane was UV cross-linked with biotinylated RNA for 2 minutes followed by detection with enhanced chemiluminescent substrates for horseradish peroxidase (HRP) from Chemiluminescent Nucleic Acid Detection Module (Pierce, Thermo Scientific), as specified in the manufacturer’s protocol.

### RNA electrophoretic mobility shift assay (REMSA)

Measurement of RNA:protein interactions was performed with the LightShift Chemiluminescent RNA EMSA Kit (Pierce, Thermo Scientific). The IRE RNA with cytosolic liver extract included in the kit was used as positive control according to the manufacturer’s instructions with some modifications. The incubated reactions included 4 nM of cellular miRNA with 4 μg lung cellular (HTB-177) protein extract, 4 nM of cellular mRNA with 8 μg lung cellular protein extract, 5 nM of cellular miRNA with 20 μg exosomal protein extract, 2 nM of cellular mRNA with 10 μg exosomal protein extract, 5 nM exosomal RNA with 20 μg exosomal protein extract. The specific unlabeled RNA competitors were added in excess (200 fold) in RNA-protein binding buffer (1X REMSA-buffer) for each of above-mentioned assays. Regarding the IRE RNA control, 6.25 nM biotinylated IRE RNA control was incubated with 4 μg of cytosolic liver extract and with 6 μg lung cellular (HTB-177 cell line) protein extract. Before adding the biotinylated RNA, the reaction components were pre-incubated for 15 minutes at room temperature (RT), and the binding reactions were incubated for 30 minutes at RT before adding the loading buffer. The binding reactions for IRE RNA and cytosolic liver extract and lung cells (HTB-177) proteins were electrophoresed on 4% and 6% DNA Retardation Pre-cast gels (Invitrogen, Life Technologies). The gels were transferred to a nylon membrane using the XCell II™ Blot Module (Invitrogen Dynal, Oslo, Norway, Life Technologies) in 0.5X TBE, at 300 mA for 45 minutes. After UV crosslinking of biotinylated RNA to the nylon membrane, the biotinylated RNA was detected with the Chemiluminescent Nucleic Acid Detection Module (Pierce, Thermo Scientific), following the manufacturer’s protocol. The excess of unlabeled RNA (200 fold) was added to the binding reactions, and specificities of the RNA-protein interactions were examined (to observe if the labelled and unlabeled RNA sequences competed for binding to the same protein).

### Purification of RNA binding proteins

For the purification of RNA Binding Proteins (RBPs), paramagnetic Dynabeads M-280 Streptavidin (Invitrogen, Life Technologies) were used. The beads were first washed in Wash and Binding Buffer (W&B) three times. For RNA application, the beads were washed twice in the same volume of Solution A, once in Solution B, and re-suspended in W&B buffer, following the manufacturer’s instructions. 100 pmol of cellular biotinylated miRNAs were mixed with 1 mg Dynabeads, 40 pmol of biotinylated exosomal RNA were mixed with 400 μg Dynabeads, and 5 pmol of labelled mRNA were incubated with 500 μg of Dynabeads. All reactions were incubated for 20 min at RT with gentle rotation. The beads were separated on a MPC-S magnet (Dynal, Invitrogen, Life Technologies), and after washing a small fraction (2 μl) of the sample was used for evaluating the binding after UV crosslinking of biotinylated RNA to the nylon membrane, by chemiluminescence. Biotinylated RNA/Dynabeads complexes were resuspended in the 2X RNA-proteins Binding Buffer (10 mM Tris-HCl (pH 7.5), 1 mM EDTA, 2 M NaCl), and one volume of cellular and exosomal proteins + H_2_O, respectively, were added in order to reach the final concentration 1X for the binding buffer, scaling the relative amount of RNA/proteins from the REMSA experiments. The samples were incubated for 30 minutes at RT, with gentle rotation. To remove unbound proteins, the samples were washed twice with 1X binding buffer. The RBPs bound to the RNA were isolated by the addition of extraction buffer (50 mM Tris-HCl pH 7.5 + 0.1% SDS) to the complex and incubated at 85°C for 5 minutes. Samples without RNA were treated equally and used as negative controls to identify unspecific proteins bound to the beads.

### Identification of proteins

For protein identification, the isolated RBPs (actual sample with RNA) or proteins from negative samples (without RNA) were collected in 5% SDS gel, stained with RuBPs, bands were excised, trypsinised and analyzed with LC-MS/MS by the Proteomics Core facility at the University of Gothenburg (http://www.proteomics.cf.gu.se/). Briefly, for the liquid chromatography an Agilent 1100 binary pump was used, together with a reversed phase column, 200 x 0.075 mm, packed in-house with 3 μm particles Reprosilpur C_18_-AQ. To separate the peptides, the flow through the column was reduced by a split followed by a 50 min gradient of 0–50% CH_3_CN. The nano-flow LC-MS/MS was done on a 7-Tesla LTQ-FT mass spectrometer (Thermo Electron) equipped with a nanospray source modified in-house. The spectrometer was operated in a data-dependent mode, automatically switching to MS/MS mode. MS-spectra were acquired in the FTICR, while MS/MS-spectra were acquired in the LTQ-trap. For each scan of FTICR, the three most intense, doubly or triply charged, ions were sequentially fragmented in the linear trap by collision induced dissociation. All the tandem mass spectra were searched by MASCOT (Matrix Science, London, UK) program to identify proteins. The analysis was repeated two times.

Samples without RNA were processed in parallel and were used as negative controls to identify proteins that bound non-specifically to the beads. The experiment was performed twice and proteins identified in both biological replicates, but not in the negative controls, were taken forward.

### Computational analysis of identified proteins

Bio-computational analysis of the proteins identified by LC-MS/MS was carried out based on their biological functions and cellular localization by using the DAVID functional annotation tool (http://www.david.abcc.ncifcrf.gov) for Gene Ontology (GO) analysis relative to RNA-related processes and cellular localization, and by literature search. To identify conserved RNA-binding domains, the specialized blast from NCBI was used, searching against Conserved Domain Database (CCD) database (http://www.ncbi.nlm.nih.gov/Structure/cdd/cdd.shtml), which includes NCBI curated 3D protein domains, as well as data from 5 among the major sources of protein families databases [52]. This analysis was further improved by searching the proteins that were identified in RBPDB database (http://rbpdb.ccbr.utoronto.ca).

Finally, the Ingenuity software (http://www.ingenuity.com) was used to build a biological network of identified RBPs in exosomes and their parental cells. Individual network analysis was performed for the identified-exosomal-RBPs interacting only with miRNAs and mRNA. Similarly, the network analysis for identified RBPs in parent cells interacting with both cell-mRNAs and cell-miRNAs was performed and biological functions were retrieved.

### Post-transcriptional gene silencing

Gene silencing in HTB-177 cells was achieved by transfecting corresponding siRNA against the mRNA transcripts of each of the six genes using HiPerFect transfection reagent from Qiagen according to the manufacturer´s protocol. Cells were plated in 6-well tissue culture plate (Sarstedt) at a density of 6 x 10^5^ cells per well and grown in the presence of Opti-MEM I Reduced Serum Media (Thermo Fisher Scientific), 10% FBS (Sigma) previously depleted of exosomes, 1% Penicillin-Streptomycin (Gibco) and 1% L-glutamine (Gibco). HiPerFect transfection reagent was mixed with siRNA and added to the cells at a final concentration of 10 nM. The list of siRNAs and QPCR-primers used in this study is presented in **Table 1**. AllStars negative control siRNA (Qiagen, Hilden, Germany) was used as non-targeting irrelevant siRNA. The transcripts were silenced in duplicate experiments except for hnRNPA2B1 and MVP, which were performed in 4-replicates.

**Table 1:** List of siRNAs and qPCR primers used in this study.

**Small interfering RNAs (siRNAs):**			
**siRNA target name**	**Qiagen cat #**	**Target sequence**	**Entrez Gene ID**
**XPO5**	SI02778510	5´-CCAGATGTTTCGAACACTAAA-3´	57510
**HSP90AB**	SI02780561	5´-CAAGAATGATAAGGCAGTTAA-3´	3326
**HNRNPA2B1**	SI00300426	5´-AATTGATGGGAGAGTAGTTGA-3´	3181
**HNRNPH1**	SI02654799	5´-CAGGTATATTGAAATCTTTAA-3´	3187
**HNRNPM**	SI000300482	5´-AAGACTTGGAAGCACAGTATT-3´	4670
**MVP**	SI03057516	5´-CACCATCGAAACGGCGGATCA-3´	9961
**QPCR primers:**			
**Target name**	**Primer sequence**		**Entrez Gene ID**
**XPO5-fw**	ATCCTGGAACACGTTGTCAAG		57510
**XPO5-rev**	CACTACAATTCGAGACAGAGCAT		
**HSP90AB1-fw**	TGGGAAGACCACTTGGCAGTCA		3326
**HSP90AB1-rev**	AGGTCAAAGGGAGCCCGACGA		
**HNRNPA2B1-fw**	GATCTGATGGATATGGCAGTGGAC		3181
**HNRNPA2B1-rev**	GAAGAAGCTCAGTATCG GCTCCTC		
**HNRNPH1-fw**	GCCGGACCGCGTAAGAGACG		3187
**HNRNPH1-rev1**	AGCCTCGCCACTTGGTCTGC		
**HNRNPM-fw1**	AGCTGCGGAAGTCCTAAACAAGCA		4670
**HNRNPM-rev**	ACCCATCCCACCAGTCGTAGCC		
**MVP-fw1**	https://www.thermofisher.com/order/genome-database/browse/gene-expression/keyword/hs00245438_m1?ICID=uc-gex-hs00245438_m1&mode=and		9961
**MVP-rev**			

After 48 hours of incubation at 37°C, the supernatants were harvested and pooled; exosomes were extracted from the conditioned cell culture supernatant from siRNA transfected cells by centrifugation at 300g for 15 minutes to pellet the cell debris; then the supernatant was ultracentrifuged at 120000g on a Beckman Optima LE80K ultracentrifuge by using Ti70 rotor for 70 minutes. The isolated exosomes were resuspended in 30μl of PBS, and were used for both their quantification, as proteins, by using the Qubit spectrofluorometer (Thermo Fisher Scientific) and the isolation of total RNA using the miRCURY™ RNA Isolation Kit–Cell & Plant (Exiqon) according to the manufacturers’ instructions. Exosomal RNA was quantified using the Quant-iT™ RiboGreen® RNA Assay Kit (Thermo Fisher Scientific) and taking advantage of a FLUOstar Omega Microplate Reader (BMG LABTECH). To express the relative amounts (normalized) of RNA present in exosomes originated from HTB-177 cells, transfected with siRNA against MVP for 48 hours in comparison with the correspondent non-targeting negative control siRNAs, for each sample, the ratio between total exosomal RNA (ng) / total exosomes (μg) was calculated.

After 48 hours of incubation at 37°C, the siRNA transfected cells were directly lysed in the wells for RNA isolation using the miRCURY™ RNA Isolation Kit–Cell & Plant (Exiqon), according to the manufacturers’ instructions. The RNA was quantified by Qubit spectrofluorometer (Thermo Fisher Scientific).

The statistical significance of exosomal and cellular RNA variation after MVP silencing was calculated by applying an unpaired t-test with Welch`s correction using the software GraphPad Prism v7.02 (https://www.graphpad.com/), the significant p-value threshold was >0.05.

### Gene expression analysis

RNA from transfected HTB-177 cells was reverse transcribed with High-Capacity cDNA Reverse Transcription Kit (Applied Biosystems). For the assessment of MVP silencing efficiency, 1 μg of total RNA from transfected HTB-177 cells was reverse transcribed with High-Capacity cDNA Reverse Transcription Kit (Applied Biosystems). Q-PCR was performed on an Applied Biosystems Real-Time PCR System Viia 7 using 100 ng of the resultant cDNA, TaqMan Fast Advanced Master Mix (Applied Biosystems) and gene specific TaqMan probe. The same procedure was used for the quantification of other silenced five genes except the use of Fast SYBRgreen PCR Master Mix (AppliedBiosystems) and gene specific primers. Gene expression was normalized to the housekeeping gene GAPDH as below:

In Q-PCR, the relative quantification of each silenced gene, after 48h siRNA-silencing, is calculated in comparison to the relative negative control samples (calibrator) and normalized to the housekeeping gene GAPDH. We used the method of **2**^-ΔΔ**CT**^:

Δct=Ct(MVP)−Ct(GAPDH)

ΔΔct=Δct(MVP)−Δct(neg.ctl.)

$$RQ=2^{\Delta \Delta ct}$$

Where RQ stands for relative quantification and represents the fold change of silenced gene, e.g. the transcripts of MVP gene. The data is presented as average fold change values (RQ). The average of four biological replicates for hnRNPA2B1 and MVP, and two biological replicates for other four transcripts were presented.

### Transient expression of MVP in HEK293F cells

HEK293F cells (Life Technologies) carrying a stable integration of the BirA (biotin ligase) coding sequence were transiently transfected with a plasmid encoding the full-length human MVP protein with a C-terminal avitag and a 10x histidine tag. Two hundred and seventy mL of cell suspension at a density of 1.1 x 106/mL were resuspended in Freestyle media in a 2L Erlenmyer flask and placed in a shaking incubator for three hours at 37°C, 8% CO2, 140 rpm. Transfection reactions were set up by incubating 200ug plasmid DNA in Optimem and 300μl 293fectin (life Technologies) in Optimem separately for 5 minutes at room temperature, before mixing and incubating for a further 20 minutes at room temperature. The DNA/293fectin complex was added to the cells that were supplemented by 300mL of Freestyle media three days later.

Six days post-transfection, the cells were harvested by centrifugation at 2500xg for 20 minutes. The cell pellets were resuspended in RIPA buffer + protease inhibitors (100ul), spun down and kept for MVP analysis from cell lysate). 1 volume of 2x lysis buffer [100 mM Tris-HCl pH 7.5, 300 mM NaCl, 3.6 mM MgCl_2_, 2 mM DTT, 160 U/mL RNasin (Promega), 2x proteases inhibitor (Complete, Mini, EDTA-free, Roche), and 0.4% Triton x-100) were added to cell pellets and resuspended well to create the cell lysate.

The supernatants were then run through a stericup filter (0.2 micron) and concentrated using tangential flow filtration with a 300kDa cut-off filter to a volume of 50mL. Ultracentrifugation was then performed to isolate exosomes at 38,000 rpm for 90 minutes at 4°C (Beckman: Ti45 rotor) and the exosome pellets were resuspended in 500μl PBS.

### MVP capture from exosomes of transfected cells

Exosomes (from transiently transfected HEK293F cells) were used for MVP analysis. 1 volume of 2x lysis buffer [100 mM Tris-HCl pH 7.5, 300 mM NaCl, 3.6 mM MgCl_2_, 2 mM DTT, 160 U/mL RNasin (Promega), 2x proteases inhibitor (Complete, Mini, EDTA-free, Roche), and 0.4% Triton x-100) were added to exosome pellets and resuspended well to create the exosome lysate. The resultant exosome lysate was then used for the MVP analysis. The biotinylated MVP from exosome lysates was captured onto streptavidin-coated Dynabeads M-280 that had been blocked with BSA and yeast tRNA to prevent the non-specific binding of proteins and RNA, respectively. For the MVP capture, 1.4 mg of total protein was incubated with 450 μL of paramagnetic Dynabeads M-280 Streptavidin (Invitrogen, Life Technologies), at 4°C for 3 hours with gentle agitation. Previously, the Dynabeads were washed and prepared for RNA manipulation following the manufacturer’s specifications, and a blocking step was performed by adding the yeast tRNA (0.1 mg/100uL beads, Invitrogen) and incubated for 1 hour at 4°C with agitation, followed by two washes with 0.01% PBS-BSA. Exosomes from untransfected HEK293F cells were processed in parallel and were included in the analysis as negative control.

After the MVP capture incubation, 511ng of denatured RNA extracted from un-transfected exosomes was added to the dynabeads-exosome samples and incubated for 1 hour at 25°C. Finally, samples were washed 4 times with PBS-BSA (0.01%), and MVP/RNA complexes were eluted from the Dynabeads adding 350 uL of lysis solution buffer (miRCURY RNA Isolation Kit, Exiqon), vortexing 15 seconds and incubating 5 min at room temperature with gentle agitation. 20 uL of eluted sample was kept for protein detection and the RNA was extracted from the remaining volume following kit specifications. Protein samples from different stages (elutes) of RBP purification were separated by SDS-PAGE and the protein bands were detected by Comassie staining. The presence of MVP in the elution buffer from different elutes/fractions was confirmed by SDS-PAGE (pull-down). RNA was quantified by Quant-iT™ RiboGreen® RNA Assay Kit (ThermoFisher Scientific).

### Statistical analysis

The statistical analysis was performed applying an unpaired t-test with Welch`s correction using the software GraphPad Prism v7.02 (https://www.graphpad.com/), considering the significant p-value threshold as >0.05.

## Results

### Identifying the interactions of exosomal-proteins with RNA species: RNA-RNP complexes

**Fig 1:**
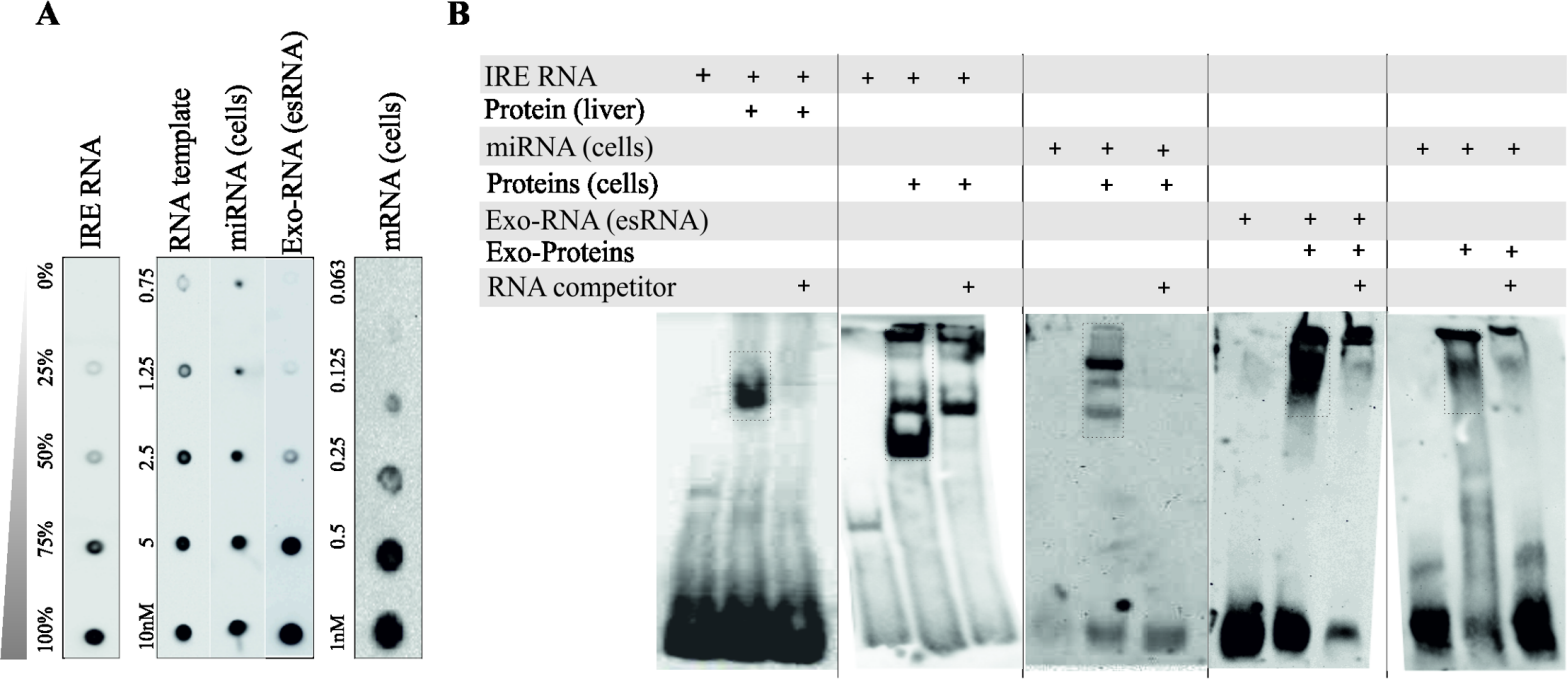
**(A) RNA 3’-end labelling of cellular (miRNA, mRNA) and exosomal-RNAs (esRNA).** The dot blot shows the efficiency of biotinylation of cellular miRNAs and mRNA, and exosomal total RNA (esRNA), relative to the biotinylated IRE control. A 43 nucleotide synthetic RNA template was used as a positive control for the reaction and as a general mass control. Serial dilutions of the labelled samples were loaded onto a nylon membrane, UV cross-linked, and detected by chemiluminescence. Dot blots show the efficiency of the RNA labelling reaction and the amount of the biotinylated RNA obtained. The biotinylation reaction for all RNA species esRNA, cell-miRNA and cell-mRNA was highly efficient, with biotinylation rates greater than 75%. **(B) RNA electrophoretic mobility shift sssays (REMSAs) of cellular and exosomal RNA-RBP-complexes:** Total exosomal-RNA (esRNA), cell-miRNA and cell-mRNA fractions extracted from HTB (lung) cells were incubated in binding reactions with exosomal proteins and with HTB cellular proteins. In all experiments, an electrophoretic shift was observed when the biotinylated RNA was incubated with the proteins from exosomes and cells. The specificities of the RNA-protein interactions were determined using competition assays. *From left to right*: the interaction of IRE RNA and cytosolic liver extract (positive control), the interaction of IRE RNA with cellular proteins, the interaction of cellular miRNAs with cellular proteins, the interaction of esRNA with exosomal proteins, and the interaction of cellular miRNA with exosomal proteins. The band-shifts are indicated by squares.

The physical interactions between exosomal-RBPs with RNAs from exosomes and RNA from their parental cells were investigated using RNA electrophoretic mobility shift assays (REMSAs). RNA from exosomes (esRNA) and RNA from their parental cells (miRNA and mRNA) were enzymatically labelled with biotin at their 3’ terminus. A 43 nucleotide synthetic RNA template and a biotinylated iron response element (IRE) RNA were used as an internal reference and positive control, respectively for the assessment of biotinylation rate. Dot blots were performed to evaluate the efficiency of the RNA labelling reaction and to calculate the amount of the biotinylated RNA obtained (**Fig 1A**). The results revealed that the biotinylation reaction for all RNA species used (i.e. esRNA, cellular miRNA and cellular mRNA) was highly efficient, with biotinylation rates greater than 75%.

To perform REMSA, the protein extracts from exosomes, HTB-177 lung cells, and liver were obtained and incubated with the biotinylated RNA species described above. A reaction containing the IRE RNA and cytosolic liver protein extract was used as a positive control because the IRE and iron-regulatory protein interaction is ubiquitous in cells. The native proteins from cells and exosomes were incubated with biotinylated iron-responsive element RNA (IRE RNA), cellular miRNA, and esRNA in separate assays. The presence of RNA-protein complexes for the different RNA species *i*.*e*. cellular miRNA, and esRNA was detected based on their mobility shifts, where each band-shift represents RNA-RNP complex (**Fig 1B**). The specificities of RNA-protein interactions were determined using competition assays, in which the excess (200 fold) of unlabeled RNA was added to the binding reactions, resulting in a decrease in the shifted signal if the labelled and unlabeled RNA sequences competed for binding to the same protein. The control samples behaved as expected (first group). Although, the same amount (200-fold) of competitor RNA was used, there was a difference in band shifts between IRE RNA + liver proteins (shift abolished, second group) and IRE RNA + cellular proteins (partial shift, third group). This difference in the shift i.e. partial can be explained by following possibility; there was an excess of cellular proteins for which the loaded competitor RNA (unlabeled, non-visible) was not enough to compete with IRE RNA (labelled, visible) and we see the partial band shift from this labeled RNA. Moreover, the liver extracts are abundant in IRP (iron-responsive protein) and bind more specifically to IRE RNA, whereas the proteins from our cell extracts are heterogeneous and not enriched in IRP as compared to liver extracts which is enrich in IRP. In addition to shift assays of esRNA and cell miRNA, although, although we identified mRNA-binding proteins in exosomes, but the gel did not show the shift for mRNA (**S1 Fig**).

### Identification of RNA binding proteins in exosomes and their parent cells

**Fig 2:**
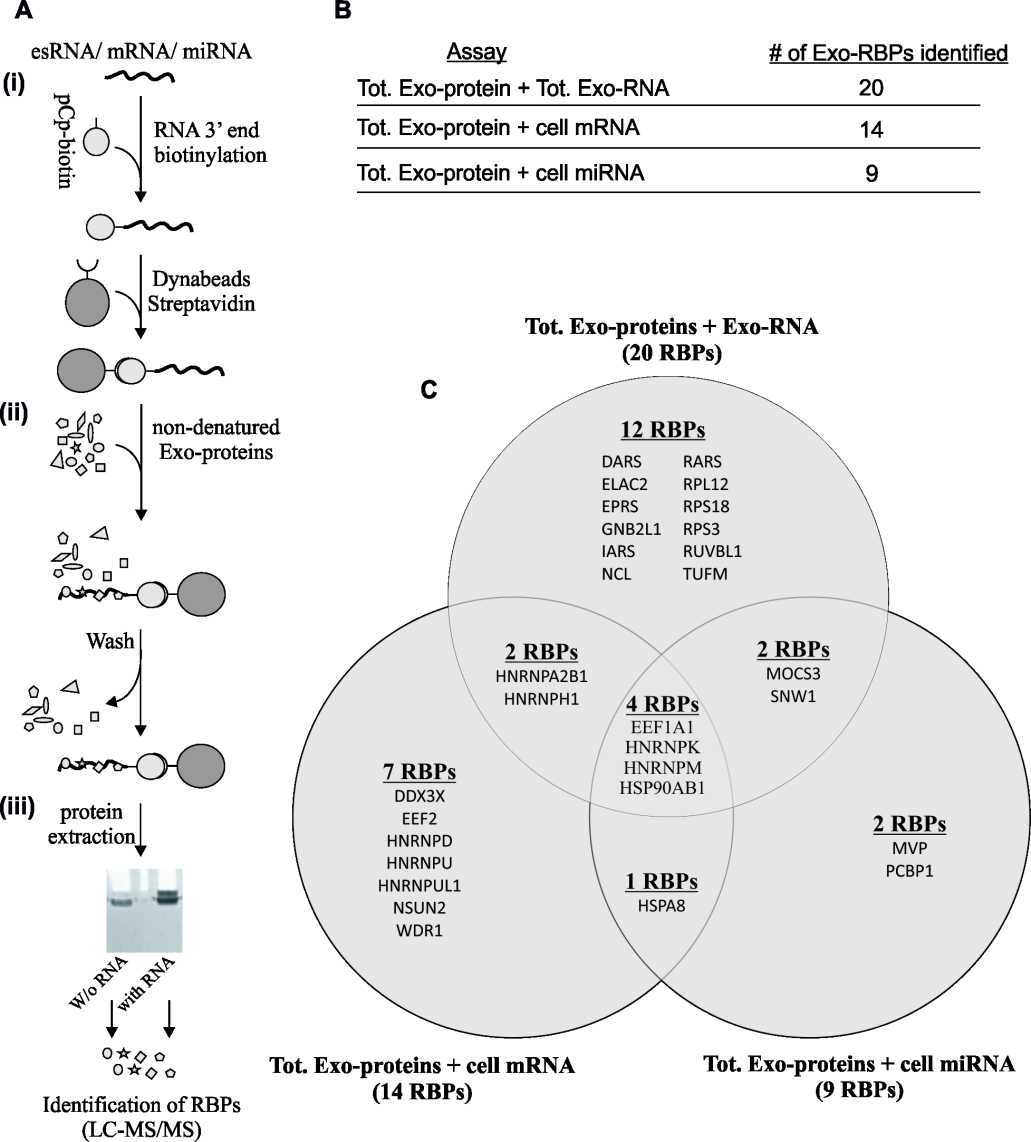
Identification of RNA-binding proteins (RBPs) from exosomes and their interactions with different RNA species. Total exosomal proteins were isolated and incubated separately in independent assays; (total exosomal protein extract + esRNA; total exosomal protein extract + cell-mRNA; total exosomal protein extract + cell-miRNA) and their interaction with RNA species were identified. From total exosomal proteins, 30 were identified as RNA-binding proteins (i.e. exosomal-RBPs). **(A)** Schematic representation of biotinylation, streptavidin and LC-MS/MS experimental procedures used to label RNA, and to identify exosomal-RBPs respectively. **(i)** Total RNA isolated from exosomes, and cells from independent assays was biotinylated and streptavidin-coated Dynabeads were added. **(ii)** The isolated native total exosomal proteins were incubated separately with esRNA, cell-mRNA and cell-miRNA, and allowed to bind with biotinylated RNAs in separate reactions. **(iii)** Proteins were re-extracted and RBPs bound to the different RNAs were then loaded onto an SDS-PAGE gel, and target bands were excised, trypsinised and analyzed using LC-MS/MS. **(B)** List of the identified exosomal-RBPs from independent assays, which interact with cellular RNA and esRNA from different assays. **(C)** Venn diagrams displaying the overlap of 30 RBPs identified in exosomal-associated with exosomal RNA (esRNA), cellular mRNAs, and cellular miRNAs. In total, 30 different RBPs were identified in exosomes, including 20 in complex with esRNA. Out of 20 esRNA-binding exosomal-RBPs, the 12 exosomal-RBPs were exclusive to esRNA, 2 were common between the esRNA and cell-mRNA samples, 2 were common between the esRNA and cell-miRNA samples and 4 RBPs were common in all three samples i.e. esRNA, cell-mRNA and cell-miRNA. 9 exosomal-RBPs in complex with cell-miRNA, 14 in complex with cell-mRNA and 1 was common between cell-miRNA and cell-mRNA samples. 2 exosomal-RBPs were in complex with cell-miRNA only, and 7 were in complex with cell-mRNA only.

To detect and isolate the RBPs in cells and exosomes, the biotinylated esRNAs, cellular miRNAs, and cellular mRNAs were attached to streptavidin-coated paramagnetic Dynabeads M-280 and were incubated with native protein extracts of exosomes in separate RNA species assays i.e. (total exosomal-protein extract + esRNA; total exosomal-protein extract + cell-mRNA; total exosomal-protein extract + cell-miRNA). The protein-RNA complexes were eluted from the beads. The RBPs bound to different RNAs were then loaded onto an SDS-PAGE gel and target bands were excised, trypsinised and analyzed using LC-MS/MS (**Fig 2A**). Samples without RNA were processed in parallel and were used as negative controls to identify proteins that bound non-specifically to the beads. The experiment was performed twice and proteins identified in both biological replicates, but not in the negative controls, were taken forward (**S1–S6 Tables**). For each assay, we compared the proteins detected in negative control and the actual sample. We detected some common proteins both in negative sample (this might be due to non-specific binding to beads) and actual sample. We excluded the common proteins that were also detected in negative sample. It is likely that they might be RBPs but we excluded them to avoid any misinterpretation of data. In total, 30 RBPs were identified from exosomes from the individual RNA species assays (**Fig 2B and 2C**). From 30 exosomal-RBPs, 20 RBPs were identified in complex with esRNA. Out of these 20 esRNA-binding exosomal RBPs, 12 were exclusive to esRNA and rest of the 8 were shared (**Fig 2C, Table 2** and **S1 Table**). 9 RBPs were identified in complex with cellular miRNA (**Fig 2C; Table 2** and **S2 Table**), and 14 RBPs were identified in complex with cellular mRNA (**Fig 2C; Table 2** and **S3 Table**). The overlapped proteins between the lists of exosomal RBPs that bind with different RNA species are shown in Venn diagrams (**Fig 2C**). Notably, four RBPs (EEFIA1, HNRNPK, HNRNPM and HSP90AB1) were common to all three subsets (identified RBPs from the assays with esRNA, cellular mRNA, and cellular miRNA). 1 exosomal-RBP was common between cell-miRNA and cell-mRNA samples. 2 exosomal-RBPs were in complex with cell-miRNA only, and 7 were in complex with cell-mRNA only.

**Table 2:** Exosomal-RBPs that formed complexes with esRNA, cell-miRNA or cell-mRNA. In total, 30 different RBPs were identified in exosomes, including 20 in complex with esRNA. Out of these 20 RBPs, 12 are exclusive to esRNA, 2 were common between the esRNA and cell-mRNA samples, 2 were common between the esRNA and cell-miRNA samples and 4 were common in all three samples i.e. esRNA, cell-mRNA and cell-miRNA. 9 exosomal-RBPs were in complex with cell-miRNA, 14 in complex with cell-mRNA and 1 was common between cell-miRNA and cell-mRNA samples. 2 exosomal-RBPs were in complex with cell-miRNA only, and 7 were in complex with cell-mRNA only. The protein domains may have distinct binding preferences to target RNA sequence motifs available at RBP database (http://rbpdb.ccbr.utoronto.ca/).

Accession	Gene Symbol	RNA-binding domain	Function	Identified in complex with:
O00571	DDX3X	-	The roles of this protein include transcriptional regulation, mRNP assembly, pre-mRNA splicing, and mRNA export. In the cytoplasm, this protein is thought to be involved in translation, cellular signaling, and viral replication.	Cell-mRNA
P13639	EEF2	-	It is an essential factor for protein synthesis. It promotes the GTP-dependent translocation of the nascent protein chain from the A-site to the P-site of the ribosome.	Cell-mRNA
O75083	WDR1	WD40	WD repeat-containing protein 1	Cell-mRNA
Q14103	hnRNPD	RRM	It is implicated in the regulation of mRNA stability	Cell-mRNA
Q00839	hnRNPU	SAP domain	This protein is thought to be involved in the packaging of hnRNA into large ribonucleoprotein complexes.	Cell-mRNA
Q9BUJ2	hnRNPUL1	SAP domain	This gene encodes a nuclear RNA-binding protein of the heterogeneous nuclear ribonucleoprotein (hnRNP) familyIt may play an important role in nucleocytoplasmic RNA transport.	Cell-mRNA
Q08J23	NSUN2	-	Catalyzes the methylation of cytosine to 5-methylcytosine (m5C) at position 34 of intron-containing tRNA(Leu)(CAA) precursors. This modification is necessary to stabilize the anticodon-codon pairing and correctly translate the mRNA.	Cell-mRNA
Q14764	MVP	-	Vaults are multi-ribonucleoproteic subunit structures that may be involved in nucleo-cytoplasmic transport.	Cell-miRNA
Q15365	PCBP1 (hnRNPE1)	KH-I	It’s involved in regulation of mRNA stability, translational regulation, tumorigenesis and cancer progression	Cell-miRNA
P14868	DARS	AspRS_cyto_N	It charges its cognate tRNA with aspartate during protein biosynthesis.	esRNA
Q9BQ52	ELAC2	-	Probably involved in tRNA maturation, by removing a 3'-trailer from precursor tRNA.	esRNA
P07814	EPRS	WEPRS_RNA	Aminoacyl-tRNA synthetase.	esRNA
P63244	GNB2L1	WD40	It contributes to the recruitment of miRISC to the site of translation	esRNA
P41252	IARS	Anticodon_Ia_Ile_ABEc	Aminoacyl-tRNA synthetase.	esRNA
P19338	NCL	RRM	Genotoxic stress activates NCL RNA-binding properties	esRNA
P54136	RARS	Anticodon_Ia_like	Aminoacyl-tRNA synthetase.	esRNA
P30050	RPL12	Ribosomal_L11	Ribosomal protein that is a component of the 60S subunit and binds directly to the 26S rRNA.	esRNA
P62269	RPS18	-	This gene encodes a ribosomal protein that is a component of the 40S subunit.	esRNA
P23396	RPS3	40S_S3_KH	It is a component of the 40S subunit, where it forms part of the domain where translation is initiated.	esRNA
Q9Y265	RUVBL1	-	RuvBL1 interacts with single-stranded DNA/RNA and double-stranded DNA	esRNA
P49411	TUFM	GTP_EFTU_D2	This protein promotes the GTP-dependent binding of aminoacyl-tRNA to the A-site of ribosomes during protein biosynthesis.	esRNA
P11142	HSPA8	-	HSC70/HSP90 complex plays a direct role in miRNAs loading to RISC	Cell-miRNA, mRNA
P22626	hnRNPA2B1	RRM	It is associated with pre-mRNAs in the nucleus, influences pre-mRNA processing, mRNA metabolism and transport.	esRNA, cell-mRNA
P31943	hnRNPH1	RRM	It was demonstrating to be directly involved in miRNA maturation	esRNA, cell-mRNA
O95396	MOCS3	-	It has a role in tRNA thiolation and molybdenum cofactor biosynthesis	esRNA, cell-miRNA
Q13573	SNW1	-	It’s a splicing factor which functions can be extended to several other steps of the mRNA processing	esRNA, cell-miRNA
P68104	EEF1A1	-	This protein is responsible for the enzymatic delivery of aminoacyl tRNAs to the ribosome.	esRNA, cell-miRNA, cell-mRNA
P61978	hnRNPK	PCBP_like_KH	It is located in the nucleoplasm. It is distinct among other hnRNP proteins in its binding preference.	esRNA, cell-miRNA, cell-mRNA
P52272	hnRNPM	RRM	The protein encoded by this gene has three repeats of quasi-RRM domains that bind to RNAs.	esRNA, cell-miRNA, cell-mRNA
P08238	HSP90AB1	-	It influences miRISC, regulating miRNA function indirectly	esRNA, cell-miRNA, cell-mRNA

The assays were also performed for protein extracts from cells i.e. (cellular-protein extract + esRNA; cellular-protein extract + cell-mRNA; cellular-protein extract + cell-miRNA) and the protein-RNA complexes were eluted from the beads and RBPs were then identified by LC-MS/MS (same method adopted in **Fig 2A**). As expected, the number of RBPs identified in the parental cells was higher than the number of RBPs in exosomes. In total, 122 RBPs were identified from cells (**S4 Table**), of which 72 were in complex with miRNA (**S5 Table**), 82 were in complex with mRNA (**S6 Table**), and 33 were common to both samples (RBPs identified in complex with cell-miRNA and cell-mRNA) (**S4 Table**). The overlap between the lists of identified RBPs in cells and exosomes are shown in Venn diagrams **(S2 Fig**).

To classify the tentative functions of these RBPs (of particular interest those found in exosomes), the list was subsequently filtered based on the predicted abilities of the proteins to interact with RNA either directly or indirectly (**Table 2**). We screened the candidate proteins for the presence of known functional RNA-binding domains (e.g., RRM, KH, RNA helicase domain, WD40, and YTH) using the NCBI tool CDS. In addition, we performed a gene ontology (GO) term molecular function analysis of all identified proteins using the DAVID annotation tool (https://david.ncifcrf.gov/), considering the GO terms related to RNA binding, processing, splicing, mRNA/ncRNA metabolic processing, and the RNP complex. The protein classification was improved further by a literature search to identify experimental evidence of the involvement of the proteins in direct or indirect RNA binding.

**Fig 3:**
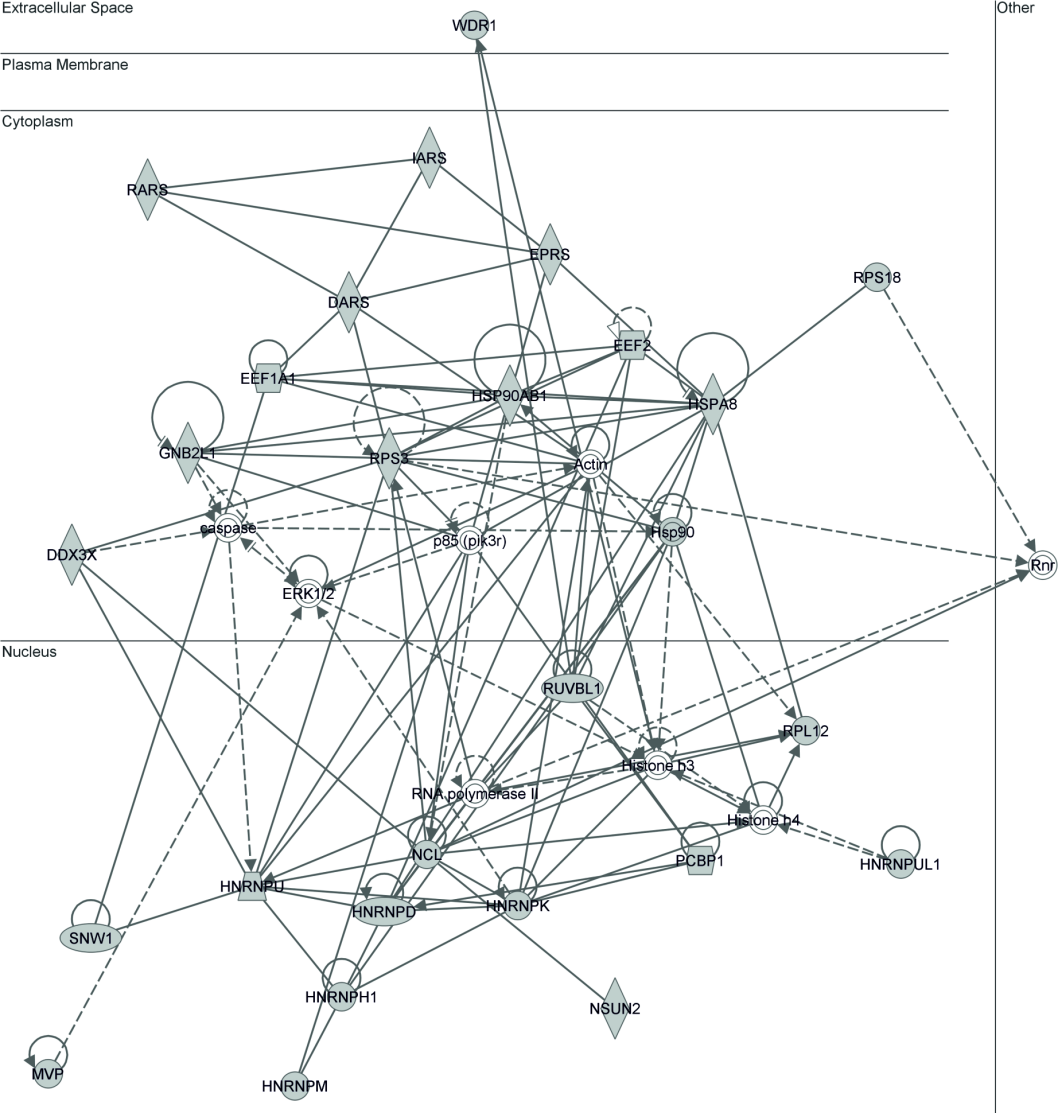
Network analysis of all (30) identified RBPs in exosomes. The key function of the identified RBPs was retrieved using Ingenuity software (www.ingenuity.com) to build a biological network of the RBPs in exosomes. The network included 35 nodes (gene products), 27 of which were among the identified exosomal-RBPs including MVP (indicated in grey). The network shows the involvement of exosomal-RBPs in ‘RNA posttranscriptional modifications’.

To understand the direct and indirect functional associations between the query protein sets, we used Ingenuity Pathway Analysis (IPA) software to build a biological network of the RBPs in exosomes and their parental cells. A key function of the exosomal-RBPs identified in this study was ‘RNA post-transcriptional modification and protein synthesis’ (**Fig 3**). The network included 35 nodes (gene products), 27 of which were among the identified exosomal-RBPs including MVP (indicated in grey). Individual network analysis was performed for the identified-exosomal-RBPs interacting only with miRNAs and mRNA (**S3 and S4 Figs** respectively). The retrieved functions showed that these RBPs are involved in ‘RNA post-transcriptional modification, protein synthesis, and RNA-transport. Similarly, the network analysis of identified RBPs in parent cells interacting with both cell-mRNAs and cell-miRNAs was performed and biological functions were retrieved (**S5 Fig and S5 Table**). The network retrieved for the cellular-RBPs identified in this study comprised 35 proteins (including MVP) involved in ‘RNA post-transcriptional modification, protein synthesis, and gene expression’. The proteins’ MS data (S1–S6 Tables) has been deposited to Vesiclepedia (http://microvesicles.org). Vesiclepedia is an integrated and comprehensive compendium of molecular data (proteome, transcriptome, and lipidome) identified in different classes of extracellular vesicles derived from different species ranging from archaea, bacteria, and eukarya, including human.

### Contribution of RBPs on exosomal RNA (esRNA) content

**Fig 4:**
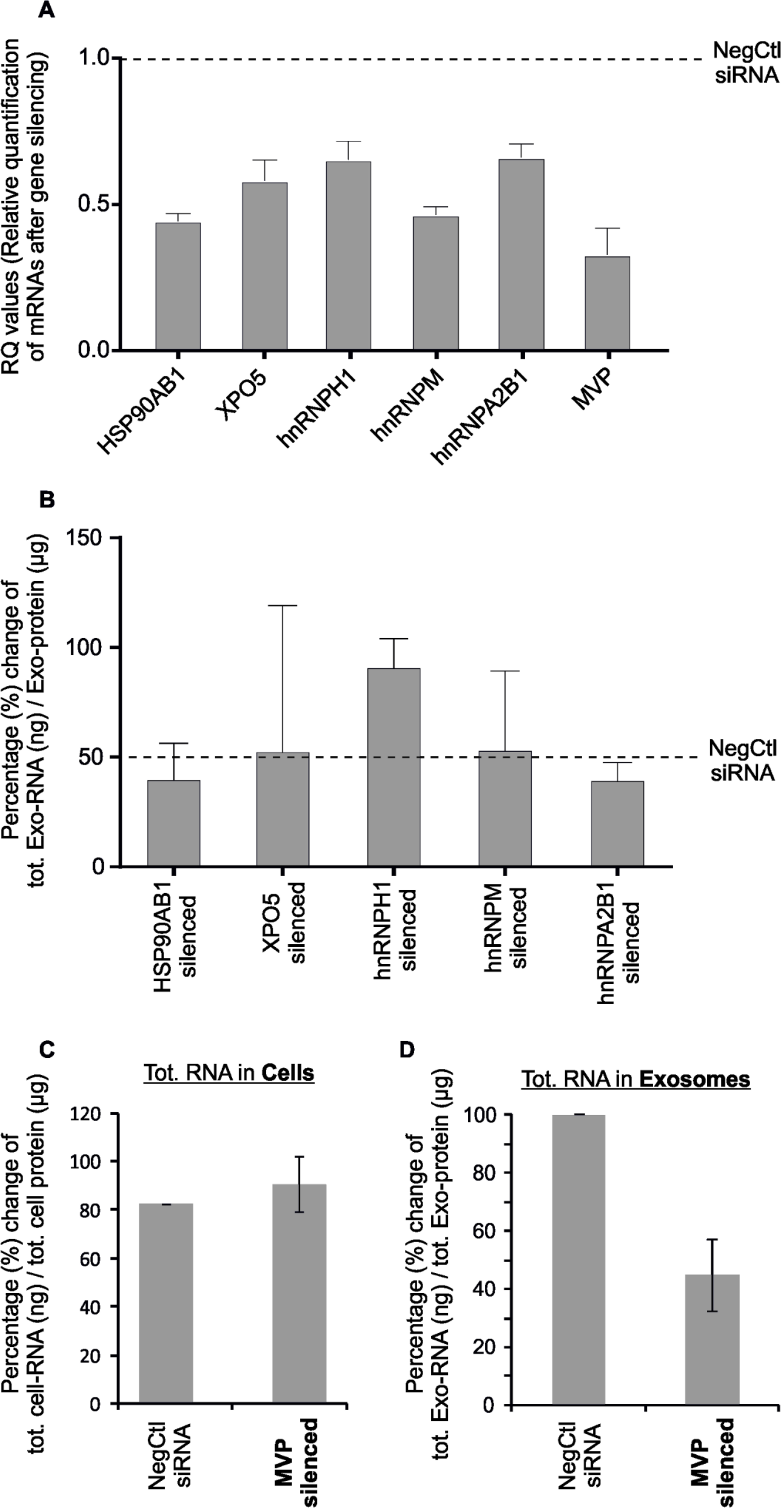
The gene transcripts encoding six RBPs identified in exosomes were silenced in the cytoplasm by siRNA and the subsequent effect on the amount of esRNA was assessed. A significant reduction of total RNA in exosomes (esRNA) was shown by post-transcriptional silencing of MVP. **(A)** Confirmation of gene silencing (silenced transcripts) in the cells after transfection with the siRNAs against the transcripts of HSP90AB1, XPO5, hnRNPH1, hnRNPM, hnRNPA2B1, and MVP with respect to the Negative Control siRNA (scrambled siRNA). Quantitative PCR was performed using cDNA, Fast SYBR Green Master Mix, and gene-specific primers. Gene expression was normalized to that of the housekeeping gene *GAPDH*. The error bars represent average of 4 biological replicates for hnRNPA2B1 and MVP, and 2 biological replicates for other four transcripts, and the average fold change values presented as RQ (relative quantification). **(B)** Quantification (the percentage-change, %) of total RNA present in exosomes (ng total esRNA / μg total exosomal proteins) after each gene silencing, *i*.*e*. silencing of HSP90AB1, XPO5, hnRNPH1, hnRNPM, and hnRNPA2B1. The silencing of hnRNPA2B1 caused a slight reduction of total RNA present in exosomes, but at non-significant level ≈13%. However, after silencing of hnRNPH1 the amount of RNA in exosomes was increased as compared to negative control. The experiment was performed in duplicates, except from hnRNPA2B1 that was performed in 4 replicates. The graph shows the range of values and median (grey). **(C-D)** Quantification of total RNA (ng total RNA / μg total protein) present in cells and exosomes after gene silencing of MVP. **(C)** A slight increase (but not significant, p-value 0.57) of the amount of total RNA in cytoplasm was observed. **(D)** On the contrary, down regulation of MVP (silencing) caused a significant reduction of the total RNA present in exosomes (esRNA), approximately by 50% (p-value 0.02). The experiment for MVP silencing was performed in four replicates; the graph shows the range of values and median (grey).

To evaluate the possible role of exosomal-RBPs in the transport of RNA into exosomes (esRNA), gene transcripts encoding six of the identified exosomal-proteins (HSP90AB1, XPO5, HNRNPH1, HNRNPM, HNRNPA2B1, and MVP) were silenced in cells using short interfering RNAs (siRNAs). XPO5 was identified only in one of the replicates but still it was included among the genes that were silenced (however not included in Table 2). These exosomal-RBPs were chosen to be analyzed for siRNA-mediated post-transcriptional analysis because they represent a class of RBPs, which are involved in different stages of mRNA and miRNA metabolism, splicing, maturation and importantly the RNA transport. The quantitative PCR analysis was performed to confirm the gene silencing, which showed a reduction of expression of gene transcripts in cells, with the MVP transcript being silenced significantly (**Fig 4A and S7 Table**).

To further determine whether the selected RBPs are involved in the transport of RNA into exosomes, the amount of RNA present in exosomes was determined after silencing. The results showed that post-transcriptional silencing of HSP90AB1, XPO5, hnRNPH1, or hnRNPM does not reduce significantly the amount total RNA present in exosomes (**Fig 4B).** Of particular note, the silencing of MVP caused a slight increase of RNA in the cytoplasm, but did not significantly affect the amount of total RNA (p-value 0.57) (**Fig 4C**). Conversely, the down regulation (silencing) of MVP caused a significant reduction in total RNA present in exosomes, approximately by 50% (p-value 0.02) (**Fig 4D**). However, after silencing of hnRNPH1 the amount of RNA in exosomes was increased as compared to negative control. Interestingly, the silencing of hnRNPA2B1, an RBP that has previously been shown for sorting of RNA into exosomes [44], in our case caused a slight decrease in RNA present in exosomes ≈13% (**Fig 4B**). The reduction of RNA amount in exosomes after the silencing of RBPs in cytoplasm indicates the possible involvement of RBPs (particularly MVP) in RNA transport into exosomes.

### Validation of MVP

**Fig 5:**
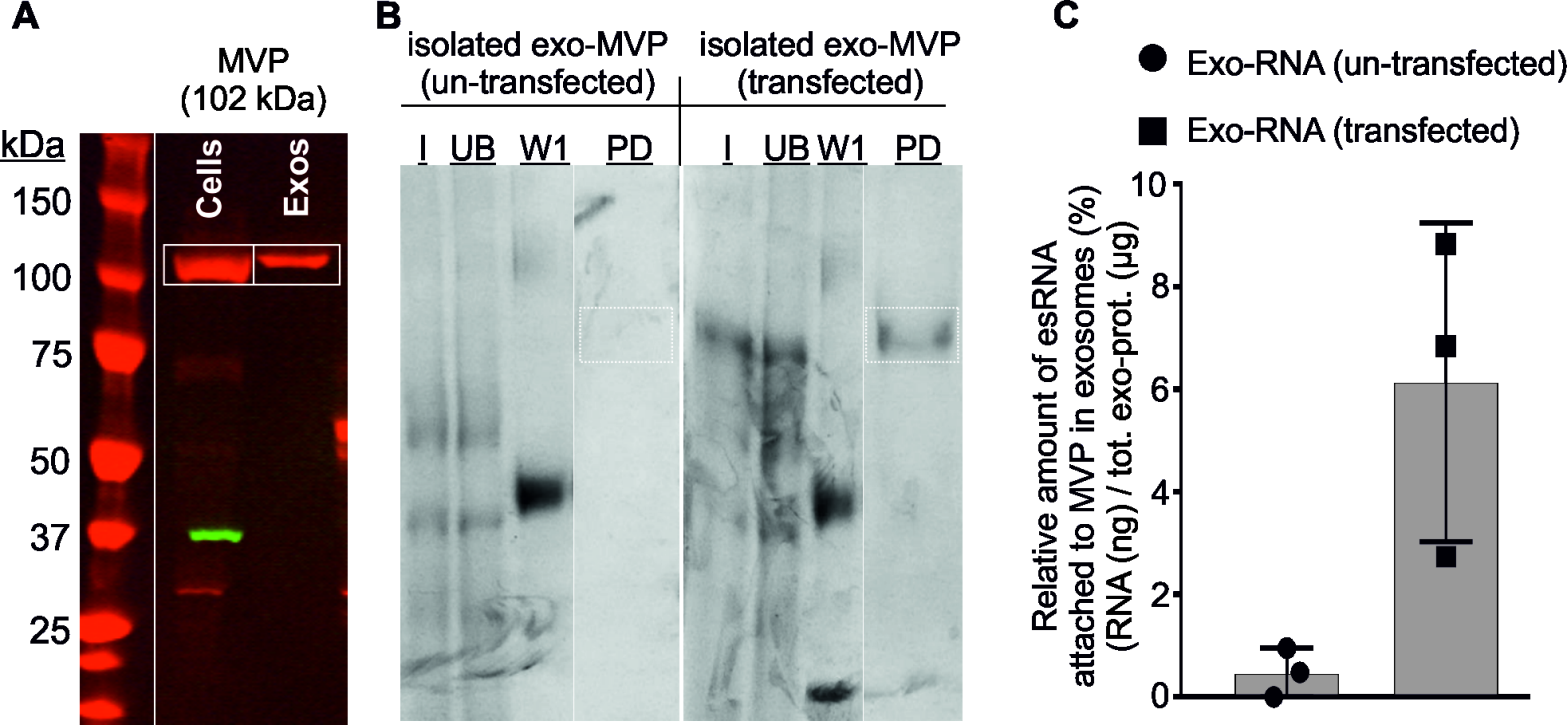
Validation of MVP. Transfected cells are expressing MVP-biotin and un-transfected cells were used as negative control. **(A)** Western blot analysis for the detection of MVP in HEK293F cell lysate and exosomal protein extracts shows that plasmid (MVP-biotin) was successfully expressed in HEK293F cells and was partitioned into exosomes isolated from these transfected cells. (**B**) Protein samples from different stages of MVP purification were separated by SDS-PAGE and the protein bands were detected by Comassie staining. After the pull down assay, the presence of MVP within exosomes was confirmed, i.e. MVP in exosomes from biotinylated MVP-transfected cells PD (pull-down lane), whereas it was absent in exosomes from untransfected cells. The position of the MVP-biotin band is indicated by squares. A representative experiment out of 3 is shown. (**C**) Total amount of RNAs coupled to MVP were extracted and quantified by Quant-iT™ RiboGreen® RNA Assay Kit (ThermoFisher Scientific). Captured RNA was expressed as a percentage of RNA eluted from the beads after the pull-down respect to the total RNA incubated with the beads. Exosomal-MVP was coupled with RNAs and the quantification of RNAs that had co-eluted with MVP showed that the amount of RNA present in the MVP elute (i.e. from MVP-RNA complex) was significantly higher than that from the untransfected control. The experiment was performed in 3-replicates and the graph shows the range of values and median (grey). Average and standard error of three independent experiments are shown. **Samples:** W = proteins from different wash steps during the MVP purification, and PD = pull-down proteins (MVP) after elution, I = input, and UB = unbound after RBP capture.

Based on our results that transcriptional-silencing of MVP causes down regulation of the RNA content in exosomes, we further investigated (i) whether, the Major Vault Protein (MVP) is in complex with RNAs within exosomes, as RNA-protein (RNPs) complexes. We transiently expressed biotinylated MVP in HEK293F cells stably expressing biotin ligase (BirA) and purified exosomes from these cells. A Western blot analysis for the detection of MVP in HEK293F cell lysate and exosomal protein extracts shows that plasmid (MVP-biotin) was successfully expressed in cells and was partitioned into exosomes when overexpressed in their parent HEK293F cells (**Fig 5A**). Next, we captured the biotinylated MVP (from lysed cells and exosomes) onto streptavidin-coated Dynabeads M-280, and eluted by pulldown assay to confirm the expression of biotinylated MVP in transfected cells and subsequent detection in exosomes. After the pull down assay, the transient expression of MVP in the cytoplasm of transfected cells was confirmed, whereas shown absent in untransfected cells (**S6A Fig,** PD (pull-down lane). After the pull down assay, the presence of MVP within exosomes from these transected cells was confirmed (**Fig 5B,** PD (pull-down lane)), whereas absent in exosomes from untransfected cells (original electrophoretogram is shown in **S6B Fig,** PD (pull-down lane)). Further, we investigated whether the exosomal-MVP was coupled with RNAs. RNAs that had co-eluted with MVP was subsequently isolated and quantified which showed that the amount of RNA present in the MVP elute (i.e. from MVP-RNA complex) was significantly higher than untransfected control (**Fig 5C**).

## Discussion

It has been demonstrated that a selective subset of RNAs egresses from cells, into the extracellular environment, via secretion of vesicles such as exosomes [17, 19, 28]. As such, there are major differences between the RNA content of exosomes and their parent cells; for example, exosomes lack 18S and 28S rRNA [17, 28–31], whereas 80% of the cytoplasmic RNA in eukaryotic cells is rRNA [53]. However, no definitive mechanism has yet been presented to reveal the key players involved in the selective loading of cytoplasmic RNAs into exosomes or the way in which this process is regulated. A transport mechanism must exist in cells to load the RNA cargo into the exosomes. For this, we hypothesize that the process of RNA transport into exosomes most likely occurs through the action of certain RBPs.

Using high-throughput protein identification and characterization, other investigators have identified nucleoproteins in exosomes listed in Vesiclepedia [54]. Based on REMSA and LC-MS/MS analyses, we identified RBPs in exosome extracts that were capable of interacting with cell-RNA, cell-miRNA and esRNA (**Fig 1B** and **Fig 2**). Because the predominant cellular content i.e. rRNA (80–85%) [53], is almost completely absent in exosomes [17], we selectively used only the cellular-miRNA and cellular-mRNA fraction of the parental cells for comparison. Total exosomal protein extracts were incubated with both cellular RNA and exosomal-RNAs. Their interactions were observed and RBPs were identified. Exosomal proteins binding to cellular RNA i.e. exosomal-RBPs could have a double purpose: (i) the RNA-binding properties of certain proteins may be maintained once they are transferred to exosomes, despite the post-transcriptional modifications that likely determine their subcellular localization [55]; and (ii) once exosomal-RBPs transferred to other cells via exosomes and released in the recipient cell, these proteins may be able to interact with their target RNAs in recipient cells. The results presented here indicate that several exosomal proteins could interact with RNAs from cells (i.e. cell-miRNA and cell-mRNA) and with RNA in exosomes (esRNA).

### The possible involvement of exosomal RBPs in post-transcriptional modifications and RNA transport

In this study, we identified 30 RBPs in exosomes that were able to bind to cellular RNA and esRNA species. Out of these 30 RBPs, twenty exosomal-RBPs bound to esRNA (12 being selective to esRNA, and 8 shared); 14 to cell-mRNA, and 9 to cell-miRNA, whereas four RBPs were common between the three groups (**Fig 2C** and **Table 2**). However, there is no clear picture how many RBPs may exist in exosomes and we may have missed identifying all RBPs in these EVs, especially RBPs that are expressed slightly in exosomes or they bind to few number of RNA molecules. Among the specific exosomal-RBPs that we identified, approximately 90% have already been reported in proteomic studies of different exosome types [56–62]. Thirty of the exosomal-RBPs are constituents of RNP complexes that are known to perform cellular functions related to several stages of RNA metabolism, such as maturation, transport, and translation. Most of the identified known RBPs are characterized by multiple localizations in cellular compartments and multifaceted functions. Notably, we identified a number of exosomal-RBPs that are members of the ubiquitously expressed hnRNP family; namely, hnRNPA2B1, hnRNPD, hnRNPH1, hnRNPK, hnRNPE1, hnRNPM, hnRNPU, and hnRNPUL1. These proteins are involved in RNA metabolism and drive pre-mRNAs through their maturation steps from the nucleus to the cytoplasm compartment, including post-transcriptional regulation, alternative splicing, transport, and localization [63]. While all members of the hnRNP family are localized to the nucleus, a number (*e*.*g*., hnRNPA2, hnRNPH1, hnRNPK, hnRNPE1, and hnRNPUL1) may play a role in nucleocytoplasmic RNA transport under specific conditions [64–67]. Moreover, hnRNPH1 is able to bind pri-miRNA molecules directly and promote pri-miRNA processing; therefore, it is directly involved in miRNA maturation [67]. Factors such as hnRNPH1-mediated RNA processing, are important for ensuring that the ratio between pre-miRNA and mature miRNA is correct [67].

Additionally, we identified a number of RBPs in exosomes that are involved in tRNA and small nucleolar RNA (snoRNA) maturation. We detected ELAC2, which catalyzes the removal of the 3’ trailer from precursor tRNAs, leading to the production of tRF-1001 [68], as well as MOCS3, which is involved in tRNA thiolation, and RUVBL1, which plays an important role in the assembly and stability of snoRNAs [69]. Moreover, four members of the aminoacyl-tRNA synthetase family (IARS, DARS, EPRS, and RARS) were identified in exosomes. The main function of these proteins is to catalyze the aminoacylation reaction during translation [70]. We also identified TUFM (Tu translation elongation factor, mitochondrial) and EEF1A1 (eukaryotic translation elongation factor 1) proteins in exosomes, both of which play a key role in translation; TUFM is a mitochondrial elongation factor, while EEF1A1 is responsible for the enzymatic delivery of aminoacyl tRNAs to the ribosomes. The interaction between these two proteins inhibits the release of EEF1A1 and leads to translational silencing [71]. The presence of proteins within exosomes that are involved in tRNA and snoRNA maturation was not surprising because exosomes contain an abundance of ncRNAs that are able to bind to Argonaute proteins to form RNA-induced silencing complexes (RISCs) [18, 72, 73]. We also identified GNB2L1, a core component of the ribosome, in exosomes. This protein acts as a molecular adaptor that is able to recruit a variety of regulators of mRNA translation, such as miRNA-induced silencing complexes, and facilitates the interactions of these regulators with the translational machinery during diverse steps of translation [74]. Moreover, a number of the RBPs detected in exosomes (HSP90AB1, HSP1A, HSPD, and HSPA8) belong to the heat shock protein family. Although this class of proteins has no recognized specific RNA-binding domains, they do play a role in miRNA-mediated gene silencing. Indeed, several heat shock proteins reside in complexes with Argonaute proteins [75]. The Hsc70 (HSPA8)-HSP90 complex is essential for RISC assembly and plays a direct role in loading miRNAs to RISCs [76].

In the context of networking and post-transcriptional modifications (**Fig 3**), recent evidence show that post-transcriptional modifications of RNA could be relevant for the sorting of RNA into exosomes [44, 77]. 3′ adenylation of miRNAs has been associated with intracellular retention whereas uridylated isoforms are enriched in exosome fractions [77]. This could suggest the specific subcellular localization of RNA-modifying proteins may have an impact on RNA sorting. Additionally, post-transcriptional modifications can contribute to sorting of specific miRNA sets into exosomes e.g., miR-2909, whereas the nature of post-transcriptional modification of miR-2909 could contribute to selective partitioning of adenosine kinase between cancer cells and their secreted exosomes [78].

The networking and presence of post-transcriptional modifier RBPs identified in our exosomes (**Fig 3**) could collaborate (could work cooperatively) to determine above-mentioned roles in RNA sorting into exosomes. Regarding post-transcriptional modification and gene synthesis in the exosome context, there are already some evidences that the exosomal proteome is enriched in proteins involved in such processes [79]. For instance, recently it has been shown that exosomes contain proteins related to RNA synthesis, translation and protein modification, which suggests that these proteins might be translated from the mRNAs after being packaged into exosomes. Since RNA processing machinery could exist in exosomes [9, 80], the existence of several exosomal proteins further shows their relation to RNA and protein synthesis and processing machinery, suggesting that exosomes harbor not only ready-to-use protein machinery but also mRNAs that can be translated if necessary [79]. In addition, the mRNAs could not only function in target cells but also be expressed in the exosomes after secretion [79]. Since it has been extensively reported in recent years that exosomes transport proteins and nucleic acids between cells and they may then be able to regulate gene expression in recipient cells. The presence of post-transcription modifiers in exosomes may help stabilize RNA shuttling (transport) between cells and epigenetic activities in recipient cells.

The key protein detected from our assays was MVP, a major component of the vault complex, an orphan protein belonging to a highly conserved ubiquitous class of RNPs of unidentified function (vaults). This protein is a multi-subunit RNP that together with other proteins forms a large macromolecular complex, a characteristic hollow barrel-like shape [81]. Because of its characteristic shape and putative function (nucleocytoplasmic transport of RNA) [81], we hypothesize that MVP may not only transport RNA into exosomes, but could also be involved in the transport of esRNA to other cells via exosomes. Several other RBPs with multiple localizations were also found in exosomes, including NCL and SNW/SKIP. NCL is involved in the maturation and processing of rRNA, and has the ability to bind to cytoplasmic mRNAs, leading to post-transcriptional regulation, the promotion of RNA stability, and the regulation of translation in the cytosol [82–85]. NW/SKIP is a splicing factor that is involved in the regulation of mRNA synthesis [86].

### The possible role of identified RBPs in the transport of RNAs into exosomes: MVP is important protein

The differences between the presentation of cellular and esRNAs indicate that esRNAs are not randomly packaged into exosomes; rather there must be a strictly regulated sorting mechanism that provides these vesicles with a subset of RNA, during their biosynthesis. It has been shown that all RNAs within cells are not naked; rather they are coupled to RBPs, as well as their associated proteins (binding partners), that affect their metabolism as well as intra-compartmental translocations. This suggests that esRNA must be coupled to proteins (i.e. they must exist as RNP complexes).

We identified RBPs in exosomes that were able to interact with different RNA species. Our assessment is that: (a) since RBPs are found bound to their related RNAs, these RBPs do not end up in exosomes alone, but together with RNA molecules as a complex of RNA and ribonucleoproteins, and (b) the presence of RBPs in exosomes could be for purpose such as the maintenance of RNAs inside exosomes and shuttling of this RNA into other cells–so called exosome shuttle RNA (esRNA) [17].

Although, RBPs have been reported to regulate exosome biogenesis, their role in RNA sorting into exosomes is only recently beginning to be explored [27, 33, 44, 45]. Notwithstanding, the mechanisms through which cytosolic RBP-RNA complexes are recognized by MVBs and are loaded into exosomes remain unknown. In our study, the presence of RBPs in exosomes suggests that, similar to cells, exosomes contain RNAs bound to their cognate proteins. These RBPs may be important for the fate and function of esRNAs, including their packaging into exosomes, stability and maintenance for their delivery to recipient cells. To evaluate the possible roles of RBPs in RNA packaging into exosomes, six gene transcripts, encoding RBPs identified in exosomes (HSP90AB1, XPO5, hnRNPH1, hnRNPM, hnRNPA2B1, and MVP), were silenced in parent cells (XPO5 was identified only in one of the replicates but still it was included among the genes that were silenced, however is not included in Table 2). Four out of six selected RBPs contain a known functional RNA-binding domain (**Table 2**) and are known to be involved in different stages of RNA metabolism, including transport, splicing, and maturation. Notably, our results revealed that selective silencing of cytoplasmic MVP significantly reduced the amount of total RNA in exosomes (**Fig 4**), indicating the possible role of MVP in RNA transport from cells to exosomes. Moreover, after the pull down assay the quantification of RNA that had co-eluted with MVP showed that the amount of esRNA present in the MVP-elute was significantly higher than esRNA from untransfected cells. Interestingly, this indicates that MVP is complexed with higher level of exosomal RNAs than exosomes from non-transfected cells (**Fig 5B and 5C**). In the current scenario, our results indicate that MVP is important in RNA transport into exosomes, however, which RNA species interact with MVP in these exosomes still remains to be answered. Studies have shown that the most abundant RNA species in exosomes is vault RNA (vRNA) and that this RNA binds to MVP [87]. In line with this observation, we speculate that MVP silencing could be preventing the packaging of vRNA and thus the reduction of total RNA in exosomes (**Fig 4D**). However, in addition to vault RNA found in exosomes, the presence of several other coding and ncRNA species have been reported in exosomes [3, 88]. MVP may get along many of them. For example, recently, it has been shown that MVP interacts with miR-193a, whereas knockout of MVP leads to miR-193a accumulation in the exosomal donor cells instead of exosomes [89]. It has also been proposed that different cell types may use RBPs with distinct binding preferences to secrete specific miRNA subsets, and perhaps other RNA classes, in exosomes [33]. Given together, MVP could be an important player in transport of RNA into exosomes; however, further studies may warrant the underlying mechanisms.

Surprisingly, after silencing of hnRNPH1 the amount of RNA in exosomes was increased as compared to negative control (**Fig 4B**). This inverse correlation of hnRNPH1 to the amount of esRNA can be hypothesized in a way that this protein could be important in feedback-loop to keep the cytoplasmic RNA levels (intracellular retention of RNA) by preventing RNA transport into exosomes. However, the current data regarding hnRNPH1 is obtained from two biological replicates and whether this protein has a possible role in cytoplasmic RNA retention would need further validation.

In addition to reduction of total exosomal RNA after MVP silencing, the silencing of hnRNPA2B1 also caused a slight reduction of total RNA present in exosomes, but at non-significant level ≈13% (**Fig 4B**). Recently, a study identified the role of sumoylated hnRNPA2B1 in the transfer of specific subset of miRNAs into exosomes from Jurkat cells [44]. However, in our experiments, we identified hnRNPA2B1 in the binding assays from exosomal-proteins and cellular mRNAs or exosomal RNA, as well as binding assays containing cellular proteins and cellular miRNAs/mRNA, but could not identify it in assays containing exosomal proteins and cellular-miRNAs (**Fig 2C**). This discrepancy of hnRNPA2B1 in the transfer of specific subset of miRNAs into exosomes could be due to steric hindrance caused by sumoylated residues in hnRNPA2B1 that prevent the protein/RNA interaction at the surface of the Dynabeads. If miRNAs are transported to the exosomes by this modified form of the protein, which is predominant but not exclusive in exosomes, we might have identified the interactions taking place between RNAs and the regular form of the protein (non-sumoylated). Alternatively, the interaction between hnRNPA2B1 and its target RNAs may be specific and targeted to certain miRNA molecules that cannot be detected by the method used here. However, further studies may rule out these discrepancies.

### The possible transport mechanism of exosomal RNA

After having shown the interactions of exosomal-RBPs with different RNA species, it indicates that the RNA found in exosomes are coupled to RBPs (i.e. they exist as RNP complexes) that we detected in our study (**Fig 1B)**, and identified the RBPs from these interaction (**Fig 2**). However, in which stage RNP-RNA complexes are inserted into exosomes–during the biosynthesis of vesicles and what are the underlying mechanisms remains unclear.

**Fig 6:**
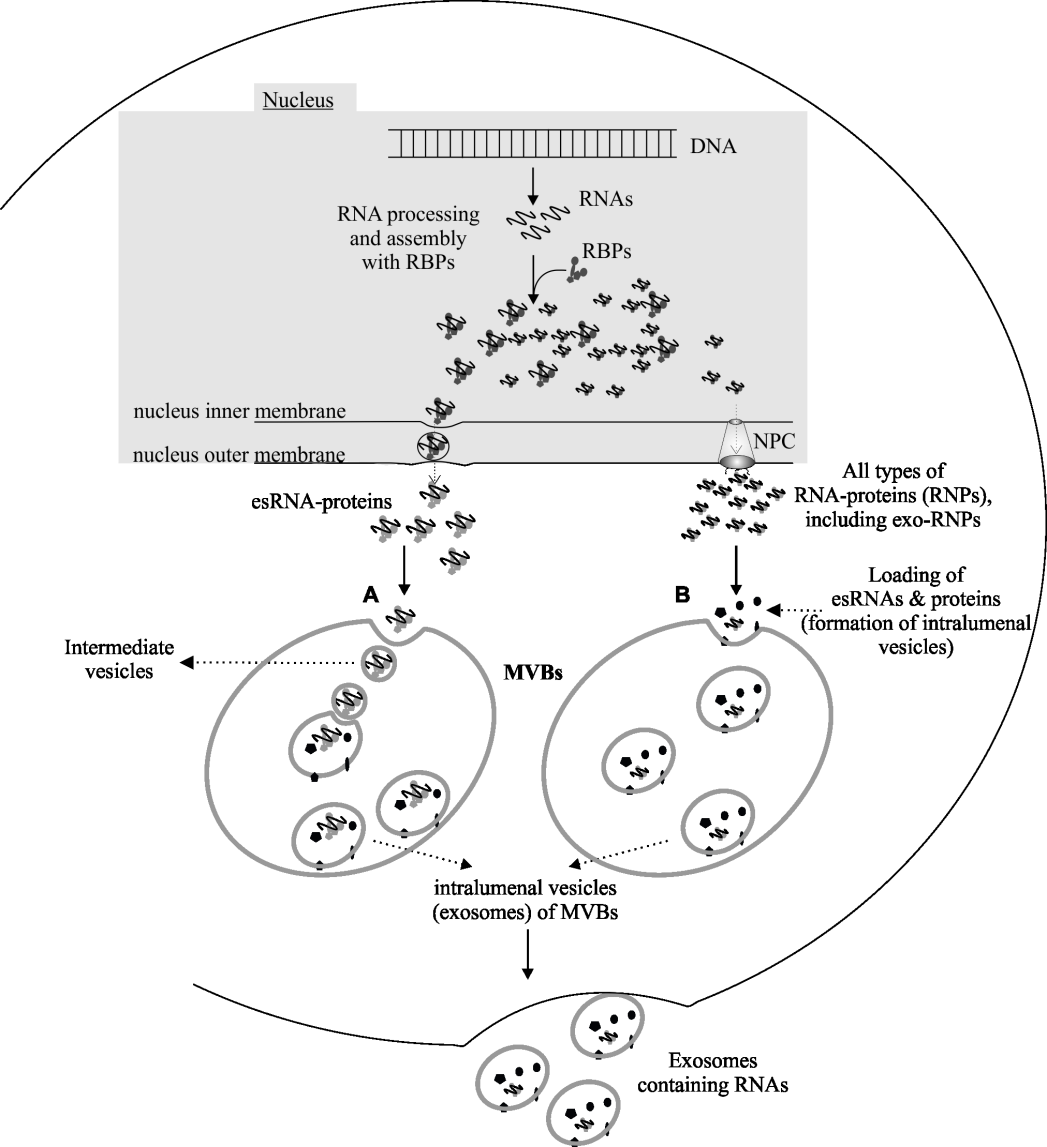
Two hypothetical paths (**A** and **B)** for the packaging of RNA–protein–complexes (RNPs) into exosomes during biosynthesis of these vesicles from the multivesicular bodies (MVBs): (**A**) The RNP-granules i.e. RNA–protein complexes to be included into exosomes (so called esRNA-protein complex) use a budding mechanism to enter into intraluminal vesicles of MVBs as RNA-protein granules. In doing so, first, they form an intermediate vesicle inside the MVBs, by inward budding–a mechanism similar to nuclear envelop (NE) budding as shown by [90–92]. The intermediate vesicles are matured and transformed into intraluminal vesicles inside MVB. Upon fusion of MVB with the plasma membrane, the intraluminal vesicles (exosomes) will be released outside containing RNA-RNPs complexes into the extracellular environment. In the second model (**B**) RNA–protein complexes (RNPs)—transported via nuclear pore complex (NPC)—are attracted to the internalization site having different receptor-proteins that could mediate inward budding of MVBs, to form intraluminal vesicles. Upon fusion with the plasma membrane, the MVBs release the intraluminal vesicles (exosomes) containing RNA–RNPs complexes into the extracellular environment.

Here, we postulate two plausible paths for the transport of RNAs into exosomes during biosynthesis of these vesicles from multivesiclur bodies (MVBs) (**Fig 6**). In the first model, RNA-protein-complexes (RNPs) could use a budding mechanism from nuclear envelop (NE) to be packaged into vesicles; termed “esRNA–protein” granules. The esRNA-protein granules could fuse and enter into MVBs forming intermediate vesicles, which later mature into intraluminal vesicles of MVBs. A similar process has been described in *Drosophila* for the existence of RNP-granules from the nucleus at the neuromuscular junction. Specifically, RNP-granules fuse with the inner nuclear membrane and bud into the nuclear envelope. Subsequently, the RNP-containing buds could migrate into the cytoplasm by fusing with the outer nuclear membrane [90]. Similarly we suggest that, in the first step of esRNA packaging, the RNP-granules (i.e. esRNA-protein complex) may form an intermediate vesicle inside the MVBs, by inward budding–similar to nuclear membrane budding. The intermediate vesicles are matured and transformed into intraluminal vesicles inside MVB. Upon fusion of MVB with the plasma membrane, the intraluminal vesicles (exosomes) will be released outside containing RNA–RNPs complexes into the extracellular environment. In the second model (**Fig 6,** right side), RNA–protein complexes (RNPs)—transported via nuclear pore complex (NPC)—are attracted to the internalization site / receptor-proteins that could mediate inward budding of MVBs, to form intraluminal vesicles. Upon fusion with the plasma membrane, the MVBs release the intraluminal vesicles as exosomes containing RNA–RNPs complexes into the extracellular environment.

Recent studies have shown the involvement of both budding of NE and NPC in the transport of RNPs from nucleus to cytoplasm [91, 92]. We hypothesize that RNPs after NE budding can further follow the similar budding into MVBs. However, to validate these paths further studies will be required.

In summary, considering the role of RBPs in RNA transport and metabolism and their recently reported role in RNA sorting into exosomes, it is paramount to understand which RBPs are crucial for RNA packaging into exosome. This also raises the possibility of the involvement of RBPs in the maintenance of RNA in exosomes for sufficient periods that they may facilitate the transfer of intact exosomal RNA to recipient cells to ensure the functional transfer of RNA. To that end, our data report several RBPs (in particular MVP) in exosomes and opens further avenues to explore the underlying mechanisms. However, our data does not show the specific sequence motifs of MVP and esRNA species. Since the formation as well as the silencing of MVP-RNA complexes have been validated and verified in all independent assays, and at least in two cells lines (HTB-177 and HEK293F), the absence of RNA sequencing to find binding motif does not alter our conclusion that MVP plays important role in RNA transport into exosomes. Shurtleff and colleagues have proposed that different cell types may use RBPs with distinct binding preferences to secrete miRNAs, and perhaps other RNA classes, in exosomes. Authors have also proposed that some cell types may deploy multiple RBPs to sort RNAs into exosomes [33]. Our data showing the identification of multiple RBPs is, in agreement to this argument, however it is unclear whether these proteins work together in combination or alone. Additionally, authors of this study argued that in this case motif discovery would be challenging, even in highly purified vesicles, due to diverse motif preferences from distinct proteins [33].

## Supporting information

S1 Fig RNA electrophoretic mobility shift assay (REMSA) showing the interaction of cellular mRNA with exosomal proteins using competition assays. Although we identified mRNA-binding proteins in exosomes, but the gel did not show the shift for mRNA.
(TIF)

S2 Fig Venn diagrams that present the overlap between the lists of identified RBPs in cells (A) and exosomes (B) capable of binding to different species of RNAs.
(TIF)

S3 Fig Network analysis of identified RBPs in exosomes interacting with cell-miRNAs. The RBPs in this network are involved in “RNA post-transcriptional modification, carbohydrate metabolism, and lipid metabolism”.
(TIF)

S4 Fig Network analysis of identified RBPs in exosomes interacting only with mRNAs. The main function of this network is “RNA post-transcriptional modification, gene expression, and protein synthesis”.
(TIF)

S5 Fig Network analysis of identified RBPs in parent cells interacting with both cell-mRNAs and cell-miRNAs. The proteins of this network are involved in “RNA post-transcriptional modification, protein synthesis, and gene expression”.
(TIF)

S6 Fig Validation of transiently expressed MVP. (A) Western blot showing the confirmation of MVP-biotin expression in transfected HEK293F cells after the pull down assay (PD (pull-down lane). (B) The presence of MVP within exosomes from biotinylated MVP-transfected cells PD (pull-down lane), whereas it was absent in exosomes from untransfected cells.
MVP-Bio: Biotinylated MVP. Samples: W = proteins from different wash steps during the MVP purification, and PD = pull-down proteins (MVP) after elution, I = input, and UB = unbound after RBP capture.
(TIF)

S1 Table All proteins identified in the assay with exosomes: “Exosomal proteins + Exosomal total RNA”. In total, 47 proteins were identified, including 20 RBPs (bold), according to GO terms and data retrieved from literature. Proteins in common with negative controls (13 proteins) are listed separately below. None of the proteins present in the negative control were RBPs.
(PDF)

S2 Table All proteins identified in the assay with exosomes: “Exosomal proteins + Cellular miRNA”. In total, 64 proteins were identified of which 9 proteins were RBPs (bold) according to the GO terms. Proteins in common with negative controls (12 proteins) are listed separately below. None of the proteins present in the negative control were RBPs.
(PDF)

S3 Table All proteins identified in the assay with exosomes: “Exosomal proteins + Cellular mRNA”. In total, 26 proteins were identified of which 14 proteins were RBPs (bold) according to the GO terms. Proteins in common with negative controls (81 proteins) are listed separately below. None of the proteins present in the negative control were RBPs.
(PDF)

S4 Table RBPs in cells identified in complex with mRNA and miRNA. 122 known RBPs were identified in total: 72 RBPs in complex with miRNA and 82 in complex with mRNA. 32 RBPs were in common in both the samples (miRNA and mRNA).
(PDF)

S5 Table All proteins identified in the assay with cell: “Cellular proteins + cellular miRNA”. In total, 157 proteins were identified, including 72 RBPs (bold), according to GO terms. Proteins in common with negative controls (33 proteins) are listed separately below. None of the proteins present in the negative control were RBPs.
(PDF)

S6 Table All proteins identified in the assay with cell: “Cellular proteins + Cellular mRNA”. In total, 238 proteins were identified of which 83 proteins were RBPs (bold) according to the GO terms. Proteins in common with negative controls (56 proteins) are listed separately below. None of the proteins present in the negative control were RBPs.
(PDF)

S7 Table MVP gene qPCR dataset after its silencing in HTB cells. The RQ values have been calculated accordingly to: (1) endogenous control GAPDH and (2) calibrator sample negative control (HTB-177 cells treated with NC-siRNA). Data from four biological replicates are shown.
(PDF)
