# Supplementary material for: Identification of RNA-binding proteins in exosomes capable of interacting with different types of RNA: RBP-facilitated transport of RNAs into exosomes
Source: PLoS One. 2018 Apr 24;13(4):e0195969. doi: 10.1371/journal.pone.0195969 (PMC5918169; doi:10.1371/journal.pone.0195969)
Supplement: S1 Table — In total, 47 proteins were identified, including 20 RBPs (bold), according to GO terms and data retrieved from literature. Proteins in common with negative controls (13 proteins) are listed separately below. None of the proteins present in the negative control were RBPs. (PDF) [file pone.0195969.s007.pdf]

S1 Table. All proteins identified in the assay with exosomes: "Exosomal proteins + Exosomal total RNA". In total, 47 proteins were identified, including 20 RBPs (bold), according to GO terms and data retrieved from literature. Proteins in common with negative controls (13 proteins) are listed separately below. None of the proteins present in the negative control were RBPs.

Accession	Gene Symbol	Description
**P14868**	**DARS**	**Aspartyl-tRNA synthetase, cytoplasmic OS=Homo sapiens GN=DARS PE=1 SV=2 - [SYDC_HUMAN]**
**P68104**	**EEF1A1**	**Elongation factor 1-alpha 1 OS=Homo sapiens GN=EEF1A1 PE=1 SV=1 - [EF1A1_HUMAN]**
**Q9BQ52**	**ELAC2**	**Zinc phosphodiesterase ELAC protein 2 OS=Homo sapiens GN=ELAC2 PE=1 SV=2 - [RNZ2_HUMAN]**
**P07814**	**EPRS**	**Bifunctional aminoacyl-tRNA synthetase OS=Homo sapiens GN=EPRS PE=1 SV=5 - [SYEP_HUMAN]**
**P63244**	**GNB2L1**	**Guanine nucleotide-binding protein subunit beta-2-like 1 OS=Homo sapiens GN=GNB2L1 PE=1 SV=3 - [GBLP_HUMAN]**
**P22626**	**HNRNPA2B1**	**Heterogeneous nuclear ribonucleoproteins A2/B1 OS=Homo sapiens GN=HNRNPA2B1 PE=1 SV=2 - [ROA2_HUMAN]**
**P31943**	**HNRNPH1**	**Heterogeneous nuclear ribonucleoprotein H OS=Homo sapiens GN=HNRNPH1 PE=1 SV=4 - [HNRH1_HUMAN]**
**P61978**	**HNRNPK**	**Heterogeneous nuclear ribonucleoprotein K OS=Homo sapiens GN=HNRNPK PE=1 SV=1 - [HNRPK_HUMAN]**
**P52272**	**HNRNPM**	**Heterogeneous nuclear ribonucleoprotein M OS=Homo sapiens GN=HNRNPM PE=1 SV=3 - [HNRPM_HUMAN]**
**P08238**	**HSP90AB1**	**Heat shock protein HSP 90-beta OS=Homo sapiens GN=HSP90AB1 PE=1 SV=4 - [HS90B_HUMAN]**
**P41252**	**IARS**	**Isoleucyl-tRNA synthetase, cytoplasmic OS=Homo sapiens GN=IARS PE=1 SV=2 - [SYIC_HUMAN]**
**O95396**	**MOCS3**	**Adenylyltransferase and sulfurtransferase MOCS3 OS=Homo sapiens GN=MOCS3 PE=1 SV=1 - [MOCS3_HUMAN]**
**P19338**	**NCL**	**Nucleolin OS=Homo sapiens GN=NCL PE=1 SV=3 - [NUCL_HUMAN]**
**P54136**	**RARS**	**Arginyl-tRNA synthetase, cytoplasmic OS=Homo sapiens GN=RARS PE=1 SV=2 - [SYRC_HUMAN]**
**P30050**	**RPL12**	**60S ribosomal protein L12 OS=Homo sapiens GN=RPL12 PE=1 SV=1 - [RL12_HUMAN]**
**P62269**	**RPS18**	**40S ribosomal protein S18 OS=Homo sapiens GN=RPS18 PE=1 SV=3 - [RS18_HUMAN]**
**P23396**	**RPS3**	**40S ribosomal protein S3 OS=Homo sapiens GN=RPS3 PE=1 SV=2 - [RS3_HUMAN]**
**Q9Y265**	**RUVBL1**	**RuvB-like 1 OS=Homo sapiens GN=RUVBL1 PE=1 SV=1 - [RUVB1_HUMAN]**
**Q13573**	**SNW1**	**SNW domain-containing protein 1 OS=Homo sapiens GN=SNW1 PE=1 SV=1 - [SNW1_HUMAN]**
**P49411**	**TUFM**	**Elongation factor Tu, mitochondrial OS=Homo sapiens GN=TUFM PE=1 SV=2 - [EFTU_HUMAN]**
O94805	ACTL6B	Actin-like protein 6B OS=Homo sapiens GN=ACTL6B PE=1 SV=1 - [ACL6B_HUMAN]
P02748	C9	Complement component C9 OS=Homo sapiens GN=C9 PE=1 SV=2 - [CO9_HUMAN]
Q494V2	CCDC37	Coiled-coil domain-containing protein 37 OS=Homo sapiens GN=CCDC37 PE=1 SV=1 - [CCD37_HUMAN]
Q96A33	CCDC47	Coiled-coil domain-containing protein 47 OS=Homo sapiens GN=CCDC47 PE=1 SV=1 - [CCD47_HUMAN]
Q8WXG9	GPR98	G-protein coupled receptor 98 OS=Homo sapiens GN=GPR98 PE=1 SV=2 -

		[GPR98_HUMAN]
P10809	HSPD1	60 kDa heat shock protein, mitochondrial OS=Homo sapiens GN=HSPD1 PE=1 SV=2 - [CH60_HUMAN]
P02545	LMNA	Prelamin-A/C OS=Homo sapiens GN=LMNA PE=1 SV=1 - [LMNA_HUMAN]
P43490	NAMPT	Nicotinamide phosphoribosyltransferase OS=Homo sapiens GN=NAMPT PE=1 SV=1 - [NAMPT_HUMAN]
P62937	PPIA	Peptidyl-prolyl cis-trans isomerase A OS=Homo sapiens GN=PPIA PE=1 SV=2 - [PPIA_HUMAN]
Q04837	SSBP1	Single-stranded DNA-binding protein, mitochondrial OS=Homo sapiens GN=SSBP1 PE=1 SV=1 - [SSBP_HUMAN]
P82094	TMF1	TATA element modulatory factor OS=Homo sapiens GN=TMF1 PE=1 SV=2 - [TMF1_HUMAN]
Q9NZR1	TMOD2	Tropomodulin-2 OS=Homo sapiens GN=TMOD2 PE=1 SV=1 - [TMOD2_HUMAN]
P68366	TUBA4A	Tubulin alpha-4A chain OS=Homo sapiens GN=TUBA4A PE=1 SV=1 - [TBA4A_HUMAN]
P04004	VTN	Vitronectin OS=Homo sapiens GN=VTN PE=1 SV=1 - [VTNC_HUMAN]
**Proteins identified in this assay, but in common with proteins in the negative control**		
P01023	A2M	Alpha-2-macroglobulin OS=Homo sapiens GN=A2M PE=1 SV=3 - [A2MG_HUMAN]
P02768	ALB	Serum albumin OS=Homo sapiens GN=ALB PE=1 SV=2 - [ALBU_HUMAN]
P04406	GAPDH	Glyceraldehyde-3-phosphate dehydrogenase OS=Homo sapiens GN=GAPDH PE=1 SV=3 - [G3P_HUMAN]
P07900	HSP90AA1	Heat shock protein HSP 90-alpha OS=Homo sapiens GN=HSP90AA1 PE=1 SV=5 - [HS90A_HUMAN]
P12259	F5	Coagulation factor V OS=Homo sapiens GN=F5 PE=1 SV=4 - [FA5_HUMAN]
P19823	ITIH2	Inter-alpha-trypsin inhibitor heavy chain H2 OS=Homo sapiens GN=ITIH2 PE=1 SV=2 - [ITIH2_HUMAN]
P23526	AHCY	Adenosylhomocysteinase OS=Homo sapiens GN=AHCY PE=1 SV=4 - [SAHH_HUMAN]
P60709	ACTB	Actin, cytoplasmic 1 OS=Homo sapiens GN=ACTB PE=1 SV=1 - [ACTB_HUMAN]
P69905	HBA1	Hemoglobin subunit alpha OS=Homo sapiens GN=HBA1 PE=1 SV=2 - [HBA_HUMAN]
Q08380	LGALS3BP	Galectin-3-binding protein OS=Homo sapiens GN=LGALS3BP PE=1 SV=1 - [LG3BP_HUMAN]
Q15582	TGFBI	Transforming growth factor-beta-induced protein ig-h3 OS=Homo sapiens GN=TGFBI PE=1 SV=1 - [BGH3_HUMAN]
Q96KK5	HIST1H2AH	Histone H2A type 1-H OS=Homo sapiens GN=HIST1H2AH PE=1 SV=3 - [H2A1H_HUMAN]
Q9Y490	TLN1	Talin-1 OS=Homo sapiens GN=TLN1 PE=1 SV=3 - [TLN1_HUMAN]
