# Supplementary material for: Identification of RNA-binding proteins in exosomes capable of interacting with different types of RNA: RBP-facilitated transport of RNAs into exosomes
Source: PLoS One. 2018 Apr 24;13(4):e0195969. doi: 10.1371/journal.pone.0195969 (PMC5918169; doi:10.1371/journal.pone.0195969)
Supplement: S2 Table — In total, 64 proteins were identified of which 9 proteins were RBPs (bold) according to the GO terms. Proteins in common with negative controls (12 proteins) are listed separately below. None of the proteins present in the negative control were RBPs. (PDF) [file pone.0195969.s008.pdf]

S2 Table. All proteins identified in the assay with exosomes: "Exosomal proteins + Cellular miRNA". In total, 64 proteins were identified of which 9 proteins were RBPs (bold) according to the GO terms. Proteins in common with negative controls (12 proteins) are listed separately below. None of the proteins present in the negative control were RBPs.

Accession	Gene Symbol	Description
**P68104**	**EEF1A1**	**Elongation factor 1-alpha 1 OS=Homo sapiens GN=EEF1A1 PE=1 SV=1 - [EF1A1_HUMAN]**
**P61978**	**HNRNPK**	**Heterogeneous nuclear ribonucleoprotein K OS=Homo sapiens GN=HNRNPK PE=1 SV=1 - [HNRPK_HUMAN]**
**P52272**	**HNRNPM**	**Heterogeneous nuclear ribonucleoprotein M OS=Homo sapiens GN=HNRNPM PE=1 SV=3 - [HNRPM_HUMAN]**
**P08238**	**HSP90AB1**	**Heat shock protein HSP 90-beta OS=Homo sapiens GN=HSP90AB1 PE=1 SV=4 - [HS90B_HUMAN]**
**P11142**	**HSPA8**	**Heat shock cognate 71 kDa protein OS=Homo sapiens GN=HSPA8 PE=1 SV=1 - [HSP7C_HUMAN]**
**O95396**	**MOCS3**	**Adenylyltransferase and sulfurtransferase MOCS3 OS=Homo sapiens GN=MOCS3 PE=1 SV=1 - [MOCS3_HUMAN]**
**Q14764**	**MVP**	**Major vault protein OS=Homo sapiens GN=MVP PE=1 SV=4 - [MVP_HUMAN]**
**Q15365**	**PCBP1**	**Poly(rC)-binding protein 1 OS=Homo sapiens GN=PCBP1 PE=1 SV=2 - [PCBP1_HUMAN]**
**Q13573**	**SNW1**	**SNW domain-containing protein 1 OS=Homo sapiens GN=SNW1 PE=1 SV=1 - [SNW1_HUMAN]**
O94805	ACTL6B	Actin-like protein 6B OS=Homo sapiens GN=ACTL6B PE=1 SV=1 - [ACL6B_HUMAN]
Q9UHI8	ADAMTS1	A disintegrin and metalloproteinase with thrombospondin motifs 1 OS=Homo sapiens GN=ADAMTS1 PE=1 SV=4 - [ATS1_HUMAN]
O00468	AGRN	Agrin OS=Homo sapiens GN=AGRN PE=1 SV=4 - [AGRIN_HUMAN]
O95831	AIFM1	Apoptosis-inducing factor 1, mitochondrial OS=Homo sapiens GN=AIFM1 PE=1 SV=1 - [AIFM1_HUMAN]
P49189	ALDH9A1	4-trimethylaminobutyraldehyde dehydrogenase OS=Homo sapiens GN=ALDH9A1 PE=1 SV=3 - [AL9A1_HUMAN]
P50995	ANXA11	Annexin A11 OS=Homo sapiens GN=ANXA11 PE=1 SV=1 - [ANX11_HUMAN]
P20073	ANXA7	Annexin A7 OS=Homo sapiens GN=ANXA7 PE=1 SV=3 - [ANXA7_HUMAN]
P31939	ATIC	Bifunctional purine biosynthesis protein PURH OS=Homo sapiens GN=ATIC PE=1 SV=3 - [PUR9_HUMAN]
Q9BXJ4	C1QTNF3	Complement C1q tumor necrosis factor-related protein 3 OS=Homo sapiens GN=C1QTNF3 PE=1 SV=1 - [C1QT3_HUMAN]
Q494V2	CCDC37	Coiled-coil domain-containing protein 37 OS=Homo sapiens GN=CCDC37 PE=1 SV=1 - [CCD37_HUMAN]
Q96A33	CCDC47	Coiled-coil domain-containing protein 47 OS=Homo sapiens GN=CCDC47 PE=1 SV=1 - [CCD47_HUMAN]
P50991	CCT4	T-complex protein 1 subunit delta OS=Homo sapiens GN=CCT4 PE=1 SV=4 -

		[TCPD_HUMAN]
P08962	CD63	CD63 antigen OS=Homo sapiens GN=CD63 PE=1 SV=2 - [CD63_HUMAN]
P11532	DMD	Dystrophin OS=Homo sapiens GN=DMD PE=1 SV=3 - [DMD_HUMAN]
P06733	ENO1	Alpha-enolase OS=Homo sapiens GN=ENO1 PE=1 SV=2 - [ENOA_HUMAN]
P50395	GDI2	Rab GDP dissociation inhibitor beta OS=Homo sapiens GN=GDI2 PE=1 SV=2 - [GDIB_HUMAN]
P62873	GNB1	Guanine nucleotide-binding protein G(I)/G(S)/G(T) subunit beta-1 OS=Homo sapiens GN=GNB1 PE=1 SV=3 - [GBB1_HUMAN]
Q8WXG9	GPR98	G-protein coupled receptor 98 OS=Homo sapiens GN=GPR98 PE=1 SV=2 - [GPR98_HUMAN]
P06899	HIST1H2BJ	Histone H2B type 1-J OS=Homo sapiens GN=HIST1H2BJ PE=1 SV=3 - [H2B1J_HUMAN]
O60814	HIST1H2BK	Histone H2B type 1-K OS=Homo sapiens GN=HIST1H2BK PE=1 SV=3 - [H2B1K_HUMAN]
P08107	HSPA1A	Heat shock 70 kDa protein 1A/1B OS=Homo sapiens GN=HSPA1A PE=1 SV=5 - [HSP71_HUMAN]
P10809	HSPD1	60 kDa heat shock protein, mitochondrial OS=Homo sapiens GN=HSPD1 PE=1 SV=2 - [CH60_HUMAN]
Q92743	HTRA1	Serine protease HTRA1 OS=Homo sapiens GN=HTRA1 PE=1 SV=1 - [HTRA1_HUMAN]
P17936	IGFBP3	Insulin-like growth factor-binding protein 3 OS=Homo sapiens GN=IGFBP3 PE=1 SV=2 - [IBP3_HUMAN]
P07942	LAMB1	Laminin subunit beta-1 OS=Homo sapiens GN=LAMB1 PE=1 SV=2 - [LAMB1_HUMAN]
P55268	LAMB2	Laminin subunit beta-2 OS=Homo sapiens GN=LAMB2 PE=1 SV=2 - [LAMB2_HUMAN]
Q14112	NID2	Nidogen-2 OS=Homo sapiens GN=NID2 PE=1 SV=3 - [NID2_HUMAN]
Q9HB63	NTN4	Netrin-4 OS=Homo sapiens GN=NTN4 PE=2 SV=2 - [NET4_HUMAN]
Q68BL7	OLFML2A	Olfactomedin-like protein 2A OS=Homo sapiens GN=OLFML2A PE=2 SV=1 - [OLM2A_HUMAN]
Q13219	PAPPA	Pappalysin-1 OS=Homo sapiens GN=PAPPA PE=1 SV=3 - [PAPP1_HUMAN]
P14618	PKM2	Pyruvate kinase isozymes M1/M2 OS=Homo sapiens GN=PKM2 PE=1 SV=4 - [KPYM_HUMAN]
P62937	PPIA	Peptidyl-prolyl cis-trans isomerase A OS=Homo sapiens GN=PPIA PE=1 SV=2 - [PPIA_HUMAN]
P62820	RAB1A	Ras-related protein Rab-1A OS=Homo sapiens GN=RAB1A PE=1 SV=3 - [RAB1A_HUMAN]
Q14563	SEMA3A	Semaphorin-3A OS=Homo sapiens GN=SEMA3A PE=2 SV=1 - [SEM3A_HUMAN]
Q9H4F8	SMOC1	SPARC-related modular calcium-binding protein 1 OS=Homo sapiens GN=SMOC1 PE=1 SV=1 - [SMOC1_HUMAN]
P78539	SRPX	Sushi repeat-containing protein SRPX OS=Homo sapiens GN=SRPX PE=2 SV=1 - [SRPX_HUMAN]
Q15526	SURF1	Surfeit locus protein 1 OS=Homo sapiens GN=SURF1 PE=1 SV=1 - [SURF1_HUMAN]
Q86XT9	TMEM219	Transmembrane protein 219 OS=Homo sapiens GN=TMEM219 PE=2 SV=1 - [TM219_HUMAN]
P82094	TMF1	TATA element modulatory factor OS=Homo sapiens GN=TMF1 PE=1 SV=2 - [TMF1_HUMAN]
Q9NZR1	TMOD2	Tropomodulin-2 OS=Homo sapiens GN=TMOD2 PE=1 SV=1 -

		[TMOD2_HUMAN]
P68371	TUBB2C	Tubulin beta-2C chain OS=Homo sapiens GN=TUBB2C PE=1 SV=1 - [TBB2C_HUMAN]
P04004	VTN	Vitronectin OS=Homo sapiens GN=VTN PE=1 SV=1 - [VTNC_HUMAN]
P61981	YWHAG	14-3-3 protein gamma OS=Homo sapiens GN=YWHAG PE=1 SV=2 - [1433G_HUMAN]
**Proteins identified in this assay, but in common with proteins in the negative control**		
P01023	A2M	Alpha-2-macroglobulin OS=Homo sapiens GN=A2M PE=1 SV=3 - [A2MG_HUMAN]
P02768	ALB	Serum albumin OS=Homo sapiens GN=ALB PE=1 SV=2 - [ALBU_HUMAN]
P02794	FTH1	Ferritin heavy chain OS=Homo sapiens GN=FTH1 PE=1 SV=2 - [FRIH_HUMAN]
P04406	GAPDH	Glyceraldehyde-3-phosphate dehydrogenase OS=Homo sapiens GN=GAPDH PE=1 SV=3 - [G3P_HUMAN]
P07900	HSP90AA1	Heat shock protein HSP 90-alpha OS=Homo sapiens GN=HSP90AA1 PE=1 SV=5 - [HS90A_HUMAN]
P19823	ITIH2	Inter-alpha-trypsin inhibitor heavy chain H2 OS=Homo sapiens GN=ITIH2 PE=1 SV=2 - [ITIH2_HUMAN]
P51884	LUM	Lumican OS=Homo sapiens GN=LUM PE=1 SV=2 - [LUM_HUMAN]
P60709	ACTB	Actin, cytoplasmic 1 OS=Homo sapiens GN=ACTB PE=1 SV=1 - [ACTB_HUMAN]
P69905	HBA1	Hemoglobin subunit alpha OS=Homo sapiens GN=HBA1 PE=1 SV=2 - [HBA_HUMAN]
Q08380	LGALS3BP	Galectin-3-binding protein OS=Homo sapiens GN=LGALS3BP PE=1 SV=1 - [LG3BP_HUMAN]
Q96KK5	HIST1H2AH	Histone H2A type 1-H OS=Homo sapiens GN=HIST1H2AH PE=1 SV=3 - [H2A1H_HUMAN]
Q9BQE3	TUBA1C	Tubulin alpha-1C chain OS=Homo sapiens GN=TUBA1C PE=1 SV=1 - [TBA1C_HUMAN]
