# Supplementary material for: Identification of RNA-binding proteins in exosomes capable of interacting with different types of RNA: RBP-facilitated transport of RNAs into exosomes
Source: PLoS One. 2018 Apr 24;13(4):e0195969. doi: 10.1371/journal.pone.0195969 (PMC5918169; doi:10.1371/journal.pone.0195969)
Supplement: S3 Table — In total, 26 proteins were identified of which 14 proteins were RBPs (bold) according to the GO terms. Proteins in common with negative controls (81 proteins) are listed separately below. None of the proteins present in the negative control were RBPs. (PDF) [file pone.0195969.s009.pdf]

S3 Table. All proteins identified in the assay with exosomes: "Exosomal proteins + Cellular mRNA". In total, 26 proteins were identified of which 14 proteins were RBPs (bold) according to the GO terms. Proteins in common with negative controls (81 proteins) are listed separately below. None of the proteins present in the negative control were RBPs.

Accession	Gene name	Description
**P68104**	**EEF1A1**	**Elongation factor 1-alpha 1 OS=Homo sapiens GN=EEF1A1 PE=1 SV=1 - [EF1A1_HUMAN]**
**P13639**	**EEF2**	**Elongation factor 2 OS=Homo sapiens GN=EEF2 PE=1 SV=4 - [EF2_HUMAN]**
**Q14103**	**HNRNPD**	**Heterogeneous nuclear ribonucleoprotein D0 OS=Homo sapiens GN=HNrnpD PE=1 SV=1 - [HNRPD_HUMAN]**
**P31943**	**HNRNPH1**	**Heterogeneous nuclear ribonucleoprotein H OS=Homo sapiens GN=HNrnpH1 PE=1 SV=4 - [HNRH1_HUMAN]**
**P61978**	**HNRNPK**	**Heterogeneous nuclear ribonucleoprotein K OS=Homo sapiens GN=HNrnpK PE=1 SV=1 - [HNRPK_HUMAN]**
**P52272**	**HNRNPM**	**Heterogeneous nuclear ribonucleoprotein M OS=Homo sapiens GN=HNrnpM PE=1 SV=3 - [HNRPM_HUMAN]**
**Q00839**	**HNRNPU**	**Heterogeneous nuclear ribonucleoprotein U OS=Homo sapiens GN=HNrnpU PE=1 SV=6 - [HNRPU_HUMAN]**
**Q9BUJ2**	**HNRNPUL1**	**Heterogeneous nuclear ribonucleoprotein U-like protein 1, OS=Homo sapiens GN=HNRNPUL1, PE=1 SV=3 - [HNRNPUL1_HUMAN]**
**P08238**	**HSP90AB1**	**Heat shock protein HSP 90-beta OS=Homo sapiens GN=HSP90AB1 PE=1 SV=4 - [HS90B_HUMAN]**
**P11142**	**HSPA8**	**Heat shock cognate 71 kDa protein OS=Homo sapiens GN=HSPA8 PE=1 SV=1 - [HSP7C_HUMAN]**
**O00571**	**DDX3X**	**ATP-dependent RNA helicase DDX3X OS=Homo sapiens GN=DDX3X PE=1 SV=3 - [DDX3X_HUMAN]**
**P22626**	**HNRNPA2B1**	**Heterogeneous nuclear ribonucleoproteins A2/B1 OS=Homo sapiens GN=HNrnpA2B1 PE=1 SV=2 - [ROA2_HUMAN]**
**Q08J23**	**NSUN2**	**tRNA (cytosine(34)-C(5))-methyltransferase OS=Homo sapiens GN=NSUN2 PE=1 SV=2 - [NSUN2_HUMAN]**
**O75083**	**WDR1**	**WD repeat-containing protein 1 OS=Homo sapiens GN=WDR1 PE=1 SV=4 - [WDR1_HUMAN]**
P04745	AMY1A	Alpha-amylase 1 OS=Homo sapiens GN=AMY1A PE=1 SV=2 - [AMY1_HUMAN]
Q06828	FMOD	Fibromodulin OS=Homo sapiens GN=FMOD PE=1 SV=2 - [FMOD_HUMAN]
P01876	IGHA1	Ig alpha-1 chain C region OS=Homo sapiens GN=IGHA1 PE=1 SV=2 - [IGHA1_HUMAN]
P05109	S100A8	Protein S100-A8 OS=Homo sapiens GN=S100A8 PE=1 SV=1 - [S10A8_HUMAN]
P27348	YWHAQ	14-3-3 protein theta OS=Homo sapiens GN=YWHAQ PE=1 SV=1 - [1433T_HUMAN]
P01031	C5	Complement C5 OS=Homo sapiens GN=C5 PE=1 SV=4 - [CO5_HUMAN]
P0C0S5	H2AFZ	Histone H2A.Z OS=Homo sapiens GN=H2AFZ PE=1 SV=2 - [H2AZ_HUMAN]
O60814	HIST1H2BK	Histone H2B type 1-K OS=Homo sapiens GN=HIST1H2BK PE=1 SV=3 - [H2B1K_HUMAN]
P04439	HLA-A	HLA class I histocompatibility antigen, A-3 alpha chain OS=Homo sapiens GN=HLA-A PE=1 SV=2 - [1A03_HUMAN]

P17987	TCP1	T-complex protein 1 subunit alpha OS=Homo sapiens GN=TCP1 PE=1 SV=1 - [TCPA_HUMAN]
Q9P0L0	VAPA	Vesicle-associated membrane protein-associated protein A OS=Homo sapiens GN=VAPA PE=1 SV=3 - [VAPA_HUMAN]
P21796	VDAC1	Voltage-dependent anion-selective channel protein 1 OS=Homo sapiens GN=VDAC1 PE=1 SV=2 - [VDAC1_HUMAN]
**Proteins identified in this assay, but in common with proteins in the negative control**		
P01023	A2M	Alpha-2-macroglobulin OS=Homo sapiens GN=A2M PE=1 SV=3 - [A2MG_HUMAN]
P60709	ACTB	Actin, cytoplasmic 1 OS=Homo sapiens GN=ACTB PE=1 SV=1 - [ACTB_HUMAN]
P23526	AHCY	Adenosylhomocysteinase OS=Homo sapiens GN=AHCY PE=1 SV=4 - [SAHH_HUMAN]
P02765	AHSG	Alpha-2-HS-glycoprotein OS=Homo sapiens GN=AHSG PE=1 SV=1 - [FETUA_HUMAN]
P14550	AKR1A1	Alcohol dehydrogenase [NADP(+)] OS=Homo sapiens GN=AKR1A1 PE=1 SV=3 - [AK1A1_HUMAN]
C9JRZ8	AKR1B15	Aldo-keto reductase family 1 member B15 OS=Homo sapiens GN=AKR1B15 PE=2 SV=1 - [AK1BF_HUMAN]
P02768	ALB	Serum albumin OS=Homo sapiens GN=ALB PE=1 SV=2 - [ALBU_HUMAN]
O75891	ALDH1L1	Cytosolic 10-formyltetrahydrofolate dehydrogenase OS=Homo sapiens GN=ALDH1L1 PE=1 SV=2 - [AL1L1_HUMAN]
P06727	APOA4	Apolipoprotein A-IV OS=Homo sapiens GN=APOA4 PE=1 SV=3 - [APOA4_HUMAN]
P02649	APOE	Apolipoprotein E OS=Homo sapiens GN=APOE PE=1 SV=1 - [APOE_HUMAN]
O95445	APOM	Apolipoprotein M OS=Homo sapiens GN=APOM PE=1 SV=2 - [APOM_HUMAN]
P05089	ARG1	Arginase-1 OS=Homo sapiens GN=ARG1 PE=1 SV=2 - [ARGI1_HUMAN]
P00966	ASS1	Argininosuccinate synthase OS=Homo sapiens GN=ASS1 PE=1 SV=2 - [ASSY_HUMAN]
O43505	B3GNT1	N-acetyllactosaminide beta-1,3-N-acetylglucosaminyltransferase OS=Homo sapiens GN=B3GNT1 PE=1 SV=1 - [B3GN1_HUMAN]
P01024	C3	Complement C3 OS=Homo sapiens GN=C3 PE=1 SV=2 - [CO3_HUMAN]
P0C0L4	C4A	Complement C4-A OS=Homo sapiens GN=C4A PE=1 SV=2 - [CO4A_HUMAN]
P31944	CASP14	Caspase-14 OS=Homo sapiens GN=CASP14 PE=1 SV=2 - [CASPE_HUMAN]
P10909	CLU	Clusterin OS=Homo sapiens GN=CLU PE=1 SV=1 - [CLUS_HUMAN]
P12109	COL6A1	Collagen alpha-1(VI) chain OS=Homo sapiens GN=COL6A1 PE=1 SV=3 - [CO6A1_HUMAN]
P49747	COMP	Cartilage oligomeric matrix protein OS=Homo sapiens GN=COMP PE=1 SV=2 - [COMP_HUMAN]
P01040	CSTA	Cystatin-A OS=Homo sapiens GN=CSTA PE=1 SV=1 - [CYTA_HUMAN]
P81605	DCD	Dermcidin OS=Homo sapiens GN=DCD PE=1 SV=2 - [DCD_HUMAN]
Q08554	DSC1	Desmocollin-1 OS=Homo sapiens GN=DSC1 PE=1 SV=2 - [DSC1_HUMAN]
Q02413	DSG1	Desmoglein-1 OS=Homo sapiens GN=DSG1 PE=1 SV=2 - [DSG1_HUMAN]
P15924	DSP	Desmoplakin OS=Homo sapiens GN=DSP PE=1 SV=3 - [DESP_HUMAN]
O43854	EDIL3	EGF-like repeat and discoidin I-like domain-containing protein 3 OS=Homo sapiens GN=EDIL3 PE=1 SV=1 - [EDIL3_HUMAN]
P06733	ENO1	Alpha-enolase OS=Homo sapiens GN=ENO1 PE=1 SV=2 - [ENOA_HUMAN]
P12259	F5	Coagulation factor V OS=Homo sapiens GN=F5 PE=1 SV=4 - [FA5_HUMAN]
P23142	FBLN1	Fibulin-1 OS=Homo sapiens GN=FBLN1 PE=1 SV=4 - [FBLN1_HUMAN]
Q86UX7	FERMT3	Fermitin family homolog 3 OS=Homo sapiens GN=FERMT3 PE=1 SV=1 - [URP2_HUMAN]

P02675	FGB	Fibrinogen beta chain OS=Homo sapiens GN=FGB PE=1 SV=2 - [FIBB_HUMAN]
P21333	FLNA	Filamin-A OS=Homo sapiens GN=FLNA PE=1 SV=4 - [FLNA_HUMAN]
P02751	FN1	Fibronectin OS=Homo sapiens GN=FN1 PE=1 SV=4 - [FINC_HUMAN]
P02794	FTH1	Ferritin heavy chain OS=Homo sapiens GN=FTH1 PE=1 SV=2 - [FRIH_HUMAN]
P04406	GAPDH	Glyceraldehyde-3-phosphate dehydrogenase OS=Homo sapiens GN=GAPDH PE=1 SV=3 - [G3P_HUMAN]
P06396	GSN	Gelsolin OS=Homo sapiens GN=GSN PE=1 SV=1 - [GELS_HUMAN]
P08263	GSTA1	Glutathione S-transferase A1 OS=Homo sapiens GN=GSTA1 PE=1 SV=3 - [GSTA1_HUMAN]
Q7RTV2	GSTA5	Glutathione S-transferase A5 OS=Homo sapiens GN=GSTA5 PE=1 SV=1 - [GSTA5_HUMAN]
P69905	HBA1	Hemoglobin subunit alpha OS=Homo sapiens GN=HBA1 PE=1 SV=2 - [HBA_HUMAN]
P68871	HBB	Hemoglobin subunit beta OS=Homo sapiens GN=HBB PE=1 SV=2 - [HBB_HUMAN]
P62805	HIST1H4A	Histone H4 OS=Homo sapiens GN=HIST1H4A PE=1 SV=2 - [H4_HUMAN]
Q86YZ3	HRNR	Hornerin OS=Homo sapiens GN=HRNR PE=1 SV=2 - [HORN_HUMAN]
P07900	HSP90AA1	Heat shock protein HSP 90-alpha OS=Homo sapiens GN=HSP90AA1 PE=1 SV=5 - [HS90A_HUMAN]
P08107	HSPA1A	Heat shock 70 kDa protein 1A/1B OS=Homo sapiens GN=HSPA1A PE=1 SV=5 - [HSP71_HUMAN]
Q13418	ILK	Integrin-linked protein kinase OS=Homo sapiens GN=ILK PE=1 SV=2 - [ILK_HUMAN]
P19827	ITIH1	Inter-alpha-trypsin inhibitor heavy chain H1 OS=Homo sapiens GN=ITIH1 PE=1 SV=3 - [ITIH1_HUMAN]
P19823	ITIH2	Inter-alpha-trypsin inhibitor heavy chain H2 OS=Homo sapiens GN=ITIH2 PE=1 SV=2 - [ITIH2_HUMAN]
Q06033	ITIH3	Inter-alpha-trypsin inhibitor heavy chain H3 OS=Homo sapiens GN=ITIH3 PE=1 SV=2 - [ITIH3_HUMAN]
Q14624	ITIH4	Inter-alpha-trypsin inhibitor heavy chain H4 OS=Homo sapiens GN=ITIH4 PE=1 SV=4 - [ITIH4_HUMAN]
P14923	JUP	Junction plakoglobin OS=Homo sapiens GN=JUP PE=1 SV=3 - [PLAK_HUMAN]
Q08380	LGALS3BP	Galectin-3-binding protein OS=Homo sapiens GN=LGALS3BP PE=1 SV=1 - [LG3BP_HUMAN]
Q07954	LRP1	Prolow-density lipoprotein receptor-related protein 1 OS=Homo sapiens GN=LRP1 PE=1 SV=2 - [LRP1_HUMAN]
P51884	LUM	Lumican OS=Homo sapiens GN=LUM PE=1 SV=2 - [LUM_HUMAN]
P61626	LYZ	Lysozyme C OS=Homo sapiens GN=LYZ PE=1 SV=1 - [LYSC_HUMAN]
Q08431	MFGE8	Lactadherin OS=Homo sapiens GN=MFGE8 PE=1 SV=2 - [MFGM_HUMAN]
P35579	MYH9	Myosin-9 OS=Homo sapiens GN=MYH9 PE=1 SV=4 - [MYH9_HUMAN]
P52209	PGD	6-phosphogluconate dehydrogenase, decarboxylating OS=Homo sapiens GN=PGD PE=1 SV=3 - [6PGD_HUMAN]
P00558	PGK1	Phosphoglycerate kinase 1 OS=Homo sapiens GN=PGK1 PE=1 SV=3 - [PGK1_HUMAN]
P36871	PGM1	Phosphoglucomutase-1 OS=Homo sapiens GN=PGM1 PE=1 SV=3 - [PGM1_HUMAN]
P14618	PKM	Pyruvate kinase PKM OS=Homo sapiens GN=PKM PE=1 SV=4 - [KPYM_HUMAN]
P06737	PYGL	Glycogen phosphorylase, liver form OS=Homo sapiens GN=PYGL PE=1 SV=4 - [PYGL_HUMAN]
P61224	RAP1B	Ras-related protein Rap-1b OS=Homo sapiens GN=RAP1B PE=1 SV=1 - [RAP1B_HUMAN]
P13489	RNH1	Ribonuclease inhibitor OS=Homo sapiens GN=RNH1 PE=1 SV=2 -

		[RINI_HUMAN]
P06702	S100A9	Protein S100-A9 OS=Homo sapiens GN=S100A9 PE=1 SV=1 - [S10A9_HUMAN]
Q6UWP8	SBSN	Suprabasin OS=Homo sapiens GN=SBSN PE=2 SV=2 - [SBSN_HUMAN]
O00560	SDCBP	Syntenin-1 OS=Homo sapiens GN=SDCBP PE=1 SV=1 - [SDCB1_HUMAN]
Q96P63	SERPINB12	Serpin B12 OS=Homo sapiens GN=SERPINB12 PE=1 SV=1 - [SPB12_HUMAN]
P29508	SERPINB3	Serpin B3 OS=Homo sapiens GN=SERPINB3 PE=1 SV=2 - [SPB3_HUMAN]
P01008	SERPINC1	Antithrombin-III OS=Homo sapiens GN=SERPINC1 PE=1 SV=1 - [ANT3_HUMAN]
P05546	SERPIND1	Heparin cofactor 2 OS=Homo sapiens GN=SERPIND1 PE=1 SV=3 - [HEP2_HUMAN]
P36955	SERPINF1	Pigment epithelium-derived factor OS=Homo sapiens GN=SERPINF1 PE=1 SV=4 - [PEDF_HUMAN]
P37802	TAGLN2	Transgelin-2 OS=Homo sapiens GN=TAGLN2 PE=1 SV=3 - [TAGL2_HUMAN]
Q15582	TGFBI	Transforming growth factor-beta-induced protein ig-h3 OS=Homo sapiens GN=TGFBI PE=1 SV=1 - [BGH3_HUMAN]
Q08188	TGM3	Protein-glutamine gamma-glutamyltransferase E OS=Homo sapiens GN=TGM3 PE=1 SV=4 - [TGM3_HUMAN]
P07996	THBS1	Thrombospondin-1 OS=Homo sapiens GN=THBS1 PE=1 SV=2 - [TSP1_HUMAN]
Q9Y490	TLN1	Talin-1 OS=Homo sapiens GN=TLN1 PE=1 SV=3 - [TLN1_HUMAN]
P68366	TUBA4A	Tubulin alpha-4A chain OS=Homo sapiens GN=TUBA4A PE=1 SV=1 - [TBA4A_HUMAN]
P07437	TUBB	Tubulin beta chain OS=Homo sapiens GN=TUBB PE=1 SV=2 - [TBB5_HUMAN]
P0CG48	UBC	Polyubiquitin-C OS=Homo sapiens GN=UBC PE=1 SV=3 - [UBC_HUMAN]
Q16851	UGP2	UTP--glucose-1-phosphate uridylyltransferase OS=Homo sapiens GN=UGP2 PE=1 SV=5 - [UGPA_HUMAN]
P63104	YWHAZ	14-3-3 protein zeta/delta OS=Homo sapiens GN=YWHAZ PE=1 SV=1 - [1433Z_HUMAN]
