# Supplementary material for: Identification of RNA-binding proteins in exosomes capable of interacting with different types of RNA: RBP-facilitated transport of RNAs into exosomes
Source: PLoS One. 2018 Apr 24;13(4):e0195969. doi: 10.1371/journal.pone.0195969 (PMC5918169; doi:10.1371/journal.pone.0195969)
Supplement: S4 Table — 122 known RBPs were identified in total: 72 RBPs in complex with miRNA and 82 in complex with mRNA. 32 RBPs were in common in both the samples (miRNA and mRNA). (PDF) [file pone.0195969.s010.pdf]

S4 Table. RBPs in cells identified in complex with mRNA and miRNA. 122 known RBPs were identified in total: 72 RBPs in complex with miRNA and 82 in complex with mRNA. 32 RBPs were in common in both the samples (miRNA and mRNA).

Accession	Gene symbol	Function	RNA-binding domain	Assay
O43324	EEF1E1	It is an auxiliary component of the macromolecular aminoacyl-tRNA synthase complex.	-	microRNA
O43776	NARS	Aminoacyl-tRNA synthetase.	-	microRNA
O43809	NUDT21	It’s interaction with the RNA is one of the earliest steps in the assembly of the 3' end processing complex and facilitates the recruitment of other processing factors.	-	microRNA
P05387	RPLP2	The protein belongs to the L12P family of ribosomal proteins. It plays an important role in the elongation step of protein synthesis.	-	microRNA
P06748	NPM1	Activation of the RNA-binding properties of nucleolin and NPM is part of the cellular response to genotoxic stress (PMID: 12000845).	-	microRNA
P07814	EPRS	Aminoacyl-tRNA synthetase	WEPRS_RNA	microRNA
P08865	RPSA	This protein is required for the processing of the 20S rRNA-precursor to mature 18S rRNA in a late step of the maturation of 40S ribosomal subunits.	RPS2	microRNA
P12081	HARS	Aminoacyl-tRNA synthetase.	HisRS_RNA	microRNA
P14868	DARS	Aspartyl-tRNA synthetase: it charges its cognate tRNA with aspartate during protein biosynthesis.	AspRS_cyto_N	microRNA
P35268	RPL22	Cytoplasmic ribosomal protein that is a component of the 60S subunit.	-	microRNA
P39019	RPS19	This ribosomal protein is a component of the 40S subunit.	-	microRNA
P41250	GARS	Aminoacyl-tRNA synthetase.	GlyRS_RNA	microRNA
P41252	IARS	Aminoacyl-tRNA synthetase.	Anticodon_Ia_Ile_ABEc	microRNA
P46783	RPS10	Ribosomal protein, component of the 40S subunit.	Plectin/S10 domain	microRNA
P47897	QARS	Aminoacyl-tRNA synthetase.	tRNA-synt_1c_C, tRNA_synt_1c_R1, tRNA_synt_1c_R2	microRNA
P52594	AGFG1	It binds the activation domain of the human immunodeficiency virus Rev protein when Rev is assembled onto its RNA target, and is required for the nuclear export of Rev-directed RNAs.	-	microRNA

P52597	HNRNPF	It belongs to the subfamily of ubiquitously expressed hnRNPs. This protein is very similar to the family member hnRPH.	RRM	microRNA
P54136	RARS	Aminoacyl-tRNA synthetase.	Anticodon_Ia_like	microRNA
P55084	HADHB	It can bind RNA and decreases the stability of some mRNAs.	-	microRNA
P62899	RPL31	Ribosomal protein that is a component of the 60S subunit. It is located in the cytoplasm.	Ribosomal_L31e	microRNA
Q01085	TIAL1	Member of a family of RNA-binding proteins. It regulates various activities including translational control, splicing and apoptosis.	RRM	microRNA
Q12904	AIMP1	The precursor protein is identical to the p43 subunit, which is associated with the multi-tRNA synthetase complex, and it modulates aminoacylation activity of tRNA synthetase in normal cells.	tRNA_bind_EMAP-II_like	microRNA
Q13148	TARDBP	It has been shown to bind both DNA and RNA and have multiple functions in transcriptional repression, pre-mRNA splicing and translational regulation.	RRM	microRNA
Q15046	KARS	Aminoacyl-tRNA synthetase.	LysRS_N	microRNA
Q32MZ4	LRRFIP1	Transcriptional repressor which preferentially binds to the GC-rich consensus sequence. It may also bind double-stranded RNA.	DUF2051	microRNA
Q6P2E9	EDC4	Enhancer of mRNA decapping 4.	WD40	microRNA
Q7Z739	YTHDF3	YTH domain is a novel RNA binding domain that binds to a short, degenerated, single-stranded RNA sequence motif (PMID: 20167602)	YTH	microRNA
Q8NC51	SERBP1	It may play a role in the regulation of mRNA stability.	HABP4_PAI-RBP1	microRNA
Q92804	TAF15	Initiation of transcription by RNA polymerase II requires the activities of more than 70 polypeptides. This gene encodes a subunit of TFIID present in a subset of TFIID complexes.	RRM	microRNA
Q92841	DDX17	DEAD box proteins are putative RNA helicases. They are implicated in a number of cellular processes involving alteration of RNA secondary structure.	DEADc	microRNA
Q92945	KHSRP	It’s a multifunctional RNA-binding protein implicated in several cellular processes, including transcription, alternative pre-mRNA splicing, and mRNA localization.	KH-I	microRNA
Q96E39	RBMXL1	Product of a retrogene of RNA binding motif protein, X-linked (RBMX), which is located on chromosome X.	RRM	microRNA
Q99729	HNRNPAB	The protein encoded by this gene has two repeats of quasi-RRM (RNA	RRM	microRNA

		recognition motif) domains that bind to RNAs.		
Q9BQ52	ELAC2	Zinc phosphodiesterase, which displays some tRNA 3' processing endonuclease activity. Probably involved in tRNA maturation, by removing a 3'-trailer from precursor tRNA.	-	microRNA
Q9BXP5	SRRT	Contributes to the stability and delivery of capped primary miRNA transcripts to the primary miRNA processing complex containing DGCR8 and DROSHA. Binds capped RNAs.	RRM	microRNA
Q9BYJ9	YTHDF1	YTH domain is a novel RNA binding domain that binds to a short, degenerated, single-stranded RNA sequence motif (PMID: 20167602)	YTH	microRNA
Q9NXH9	TRMT1	This protein dimethylates a single guanine residue at position 26 of most tRNAs using S-adenosyl-L-methionine as donor of the methyl groups.	Znf_CCCH	microRNA
Q9UMS4	PRPF19	It plays a role in DNA double-strand break (DSB) repair and pre-mRNA splicing reaction.	WD40	microRNA
Q9UQ80	PA2G4	This RNA-binding protein is involved in growth regulation; it may be involved in ribosome assembly and the regulation of intermediate and late steps of rRNA processing.	-	microRNA
Q9Y5A9	YTHDF2	YTH domain is a novel RNA binding domain that binds to a short, degenerated, single-stranded RNA sequence motif (PMID: 20167602)	YTH	microRNA
P05388	RPLP0	Ribosomal protein that is a component of the 60S subunit. It is located in the cytoplasm.	Ribosomal_P0_L10e	microRNA, mRNA
P07900	HSP90AA1	HSP90 is known to stabilize unloaded AGO2 and influences miRISC, regulating miRNA function indirectly (PMID: 21822212).	-	microRNA, mRNA
P08238	HSP90AB1	HSP90 is known to stabilize unloaded AGO2 and influences miRISC, regulating miRNA function indirectly (PMID: 21822212).	-	microRNA, mRNA
P09651	HNRNPA1	It is involved in the packaging of pre-mRNA into hnRNP particles, transport of poly A+ mRNA from the nucleus to the cytoplasm, and may modulate splice site selection.	RRM	microRNA, mRNA
P11142	HSPA8	HSP70(HSPA8)/HSP90 complex is essential for the RISC assembly, playing a direct role in miRNAs loading to RISC (PMID: 20605501)	-	microRNA, mRNA
P13489	RNH1	RNH may be essential for control of mRNA turnover	microRNA, mRNA	
P13639	EEF2	It is an essential factor for protein synthesis. It promotes the GTP-	-	microRNA, mRNA

		dependent translocation of the nascent protein chain from the A-site to the P-site of the ribosome.		
P19338	NCL	Nucleolin is a multidomain phosphoprotein involved in ribosome biogenesis. (PMID: 15170331).	RRM	microRNA, mRNA
P22626	HNRNPA2B1	This protein is associated with pre-mRNAs in the nucleus and appears to influence pre-mRNA processing and other aspects of mRNA metabolism and transport.	RRM	microRNA, mRNA
P23246	SFPQ	It is an essential pre-mRNA splicing factor required early in spliceosome formation. (PMID: 8449401).	RRM	microRNA, mRNA
P26599	PTBP1	It binds to the intronic polypyrimidine tracts that requires pre-mRNA splicing; it may also promote the binding of U2 snRNP to pre-mRNAs.	RRM, hnRNP-L_PTB	microRNA, mRNA
P29692	EEF1D	The elongation factor-1 complex is responsible for the enzymatic delivery of aminoacyl tRNAs to the ribosome.	-	microRNA, mRNA
P30050	RPL12	Ribosomal protein that is a component of the 60S subunit and binds directly to the 26S rRNA.	Ribosomal_L11	microRNA, mRNA
P31942	HNRNPH3	It is involved in the splicing process and it also participates in early heat shock-induced splicing arrest by transiently leaving the hnRNP complexes.	RRM	microRNA, mRNA
P31943	HNRNPH1	It has three repeats of quasi-RRM domains that bind to RNAs. It is very similar to the family member HNRPF.	RRM	microRNA, mRNA
P49411	TUFM	This protein promotes the GTP-dependent binding of aminoacyl-tRNA to the A-site of ribosomes during protein biosynthesis.	GTP_EFTU_D2	microRNA, mRNA
P52272	HNRNPM	The protein encoded by this gene has three repeats of quasi-RRM domains that bind to RNAs.	RRM	microRNA, mRNA
P56192	MARS	Aminoacyl-tRNA synthetase class I.	MetRS_RNA	microRNA, mRNA
P60842	EIF4A1	It is a subunit of the eIF4F complex involved in cap recognition and is required for mRNA binding to ribosome	-	microRNA, mRNA
P61978	HNRNPK	It is located in the nucleoplasm and has three repeats of KH domains that bind to RNAs. It is distinct among other hnRNP proteins in its binding preference.	PCBP_like_KH	microRNA, mRNA
P62249	RPS16	This ribosomal protein is a component of the 40S subunit.	-	microRNA, mRNA
P62826	RAN	RAN (ras-related nuclear protein) belongs to the RAS superfamily that is essential for the translocation of RNA and proteins through the	-	microRNA, mRNA

		nuclear pore complex.		
P68104	EEF1A1	It is an isoform of the alpha subunit of the elongation factor-1 complex, which is responsible for the enzymatic delivery of aminoacyl tRNAs to the ribosome	-	microRNA, mRNA
Q00839	HNRNPU	This protein is thought to be involved in the packaging of hnRNA into large ribonucleoprotein complexes.	-	microRNA, mRNA
Q08211	DHX9	It localizes to both the nucleus and the cytoplasm and functions as a transcriptional regulator. It may also be involved in the expression and nuclear export of retroviral RNAs.	DSRM, DEXDc, HA2	microRNA, mRNA
Q14103	HNRNPD	It is implicated in the regulation of mRNA stability	RRM	microRNA, mRNA
Q15365	PCBP1 (HNRNPE1)	Together with PCBP-2 and hnRNPK, this protein corresponds to the major cellular poly(rC)-binding protein.	KH-I	microRNA, mRNA
Q96AE4	FUBP1	It is a ssDNA binding protein that activates the far upstream element (FUSE) of c-myc and stimulates expression of c-myc in undifferentiated cells.	KH-I	microRNA, mRNA
Q9NSD9	FARSB	Aminoacyl-tRNA synthetase.	-	microRNA, mRNA
Q9P2J5	LARS	Cytosolic leucine-tRNA synthetase.	Anticodon_Ia_Leu_AEc	microRNA, mRNA
Q9Y265	RUVBL1	RuvBL1 interacts with single-stranded DNA/RNA and double-stranded DNA (PMID: 17060327).	-	microRNA, mRNA
Q9Y285	FARSA	This protein, member of the tRNA synthetase gene family, is expressed in a tumor-selective and cell cycle stage- and differentiation-dependent manner.	-	microRNA, mRNA
O00571	DDX3X	RNA helicase which nuclear roles include transcriptional regulation, mRNP assembly, pre-mRNA splicing, and mRNA export.	-	mRNA
O15427	SLC16A3	Identified as an mRNA interacting protein in HeLa cells (PMID: 22658674)	-	mRNA
P02786	TFRC	Identified as an mRNA interacting protein in HeLa cells (PMID: 22658674)	-	mRNA
P07910	HNRNPC	The heterotetramer of hnRNPC1/C2 measures the length of the transcripts like a molecular ruler, by selectively binding to the unstructured RNA regions longer than 200 to 300 nucleotides, thus classifying RNA polymerase II transcripts for export (PMID: 22461616).	RRM	mRNA
P08195	SLC3A2	Identified as an mRNA interacting protein in HeLa cells (PMID:	-	mRNA

		22658674)		
P0CW22	RPS17L		Ribosomal_S17e	mRNA
P17844	DDX5	RNA helicase which is a RNA-dependent ATPase, and also a proliferation-associated nuclear antigen	-	mRNA
P18077	RPL35A	Ribosomal protein, component of the 60S subunit.	Ribosomal_L35Ae	mRNA
P18621	RPL17		Ribosomal_L22	mRNA
P22087	FBL	IT's a component of a nucleolar small nuclear ribonucleoprotein (snRNP) particle thought to participate in the first step in processing preribosomal RNA.	-	mRNA
P26373	RPL13	Ribosomal protein, component of the 60S subunit.	Ribosomal_L13e	mRNA
**P27635**	**RPL10**	Ribosomal protein, component of the 40S subunit.	-	mRNA
P36578	RPL4		Ribos_L4_asso_C	mRNA
P38919	EIF4A3	RNA helicase involved in RNA metabolism.	HELICc	mRNA
P39023	RPL3		Ribosomal_L3	mRNA
P42704	LRPPRC	Binds mature or unprocessed mitochondrial mRNA in vivo and is often found associated with another smaller RNA-binding protein, SLIRP. The complex regulates the lenght of the poly(A) tail (PMID: 25008111).	-	mRNA
P46778	RPL21		Ribosomal_L21e	mRNA
P46779	RPL28		Ribosomal_L28e	mRNA
P46781	RPS9		Ribosomal_S4	mRNA
P46782	RPS5			mRNA
P50914	RPL14		Ribosomal_L14e	mRNA
P54886	ALDH18A1	Identified as an mRNA interacting protein in HeLa cells (PMID: 22658674)	-	mRNA
P61353	RPL27		Ribosomal_L27e	mRNA
P62081	RPS7		Ribosomal_S7e	mRNA
P62241	RPS8		Ribosomal_S8e_like	mRNA
P62244	RPS15A		-	mRNA
P62266	RPS23		Ribosomal_S23	mRNA
P62277	RPS13	Ribosomal protein, component of the 40S subunit.	Ribosomal_S15p_S13e	mRNA
P62753	RPS6		Ribosomal_S6e	mRNA
P62829	RPL23		Ribosomal_L24e_L24	mRNA
P62851	RPS25		Ribosomal_S25	mRNA
P62854	RPS26		Ribosomal_S26e	mRNA
P62906	RPL10A	Ribosomal protein, component of the 60S subunit.	Ribosomal_L1	mRNA
P62913	RPL11	Ribosomal protein, component of the 60S subunit that associates with the 5S rRNA.	Ribosomal_L5	mRNA

P62917	RPL8		Ribosomal_L2	mRNA
P83731	RPL24		Ribosomal_L24e_L24	mRNA
Q02878	RPL6		Ribosomal_L6e_N	mRNA
Q07020	RPL18		Ribosomal_L18e	mRNA
Q08J23	NSUN2	Catalyzes the methylation of cytosine to 5-methylcytosine (m5C) at position 34 of intron-containing tRNA(Leu)(CAA) precursors. This modification is necessary to stabilize the anticodon-codon pairing and correctly translate the mRNA.	NB multidomain !!! 25S_rRNA_cytosine2870 -C5-methyltransferase	mRNA
**Q14764**	**MVP**	Vaults are multi-ribonucleoproteic subunit structures that may be involved in nucleo-cytoplasmic transport.	-	mRNA
Q16891	IMMT	Identified as an mRNA interacting protein in HeLa cells (PMID: 22658674)	-	mRNA
Q5JTH9	RRP12		-	mRNA
Q6P2Q9	PRPF8	This protein is essential for the catalytic step II in pre-mRNA splicing process.	RRM_4, U6-snRNA_bdg, U5_2-snRNA_bdg,	mRNA
Q92499	DDX1	RNA helicase that acts as key modulator in miRNA maturation (PMID: 25176654)	-	mRNA
Q92616	GCN1L1	Acts as a translation activator that mediates translational control and perform an EF3-related function on the ribosome by regulating GCN2 protein kinase.	-	mRNA
Q92621	NUP205	This protein is part of the nuclear pore and is involved in mRNA nuclear export.	-	mRNA
Q9NSE4	IARS2	Aminoacyl-tRNA synthetase.	Anticodon_Ia_Ile_BEm	mRNA
Q9UKM9	RALY	Member of the heterogeneous nuclear ribonucleoprotein (hnRNP) gene family. This protein may play a role in pre-mRNA splicing and in embryonic development.	RRM	mRNA
Q9Y3I0	RTCB (HSPC117)	A complex frormed by HSPC117, hCLE, DDX1 and FAM98B shuttles between the nucleus and the cytoplasm transporting RNAs, suggesting that this complex has a prominent role on nuclear and cytoplasmic RNA fate (PMID: 24608264).	-	mRNA
Q9Y3U8	RPL36		Ribosomal_L36e	mRNA
