# Supplementary material for: Identification of RNA-binding proteins in exosomes capable of interacting with different types of RNA: RBP-facilitated transport of RNAs into exosomes
Source: PLoS One. 2018 Apr 24;13(4):e0195969. doi: 10.1371/journal.pone.0195969 (PMC5918169; doi:10.1371/journal.pone.0195969)
Supplement: S5 Table — In total, 157 proteins were identified, including 72 RBPs (bold), according to GO terms. Proteins in common with negative controls (33 proteins) are listed separately below. None of the proteins present in the negative control were RBPs. (PDF) [file pone.0195969.s011.pdf]

S5 Table. All proteins identified in the assay with cell: "Cellular proteins + cellular miRNA". In total, 157 proteins were identified, including 72 RBPs (bold), according to GO terms. Proteins in common with negative controls (33 proteins) are listed separately below. None of the proteins present in the negative control were RBPs.

Accession	Gene name	Description
**P52594**	**AGFG1**	**Arf-GAP domain and FG repeats-containing protein 1 OS=Homo sapiens GN=AGFG1 PE=1 SV=2 - [AGFG1_HUMAN]**
**Q12904**	**AIMP1**	**aminoacyl tRNA synthetase complex-interacting multifunctional protein 1**
**P14868**	**DARS**	**aspartyl-tRNA synthetase**
**Q92841**	**DDX17**	**Probable ATP-dependent RNA helicase DDX17 OS=Homo sapiens GN=DDX17 PE=1 SV=1 - [DDX17_HUMAN]**
**Q08211**	**DHX9**	**ATP-dependent RNA helicase A OS=Homo sapiens GN=DHX9 PE=1 SV=4 - [DHX9_HUMAN]**
**Q6P2E9**	**EDC4**	**Enhancer of mRNA-decapping protein 4 OS=Homo sapiens GN=EDC4 PE=1 SV=1 - [EDC4_HUMAN]**
**P68104**	**EEF1A1**	**eukaryotic translation elongation factor 1 alpha-like 7; eukaryotic translation elongation factor 1 alpha-like 3; similar to eukaryotic translation elongation factor 1 alpha 1; eukaryotic translation elongation factor 1 alpha 1**
**P29692**	**EEF1D**	**Elongation factor 1-delta OS=Homo sapiens GN=EEF1D PE=1 SV=5 - [EF1D_HUMAN]**
**O43324**	**EEF1E1**	**eukaryotic translation elongation factor 1 epsilon 1**
**P13639**	**EEF2**	**Elongation factor 2 OS=Homo sapiens GN=EEF2 PE=1 SV=4 - [EF2_HUMAN]**
**P60842**	**EIF4A1**	**Eukaryotic initiation factor 4A-I OS=Homo sapiens GN=EIF4A1 PE=1 SV=1 - [IF4A1_HUMAN]**
**Q9BQ52**	**ELAC2**	**Zinc phosphodiesterase ELAC protein 2 OS=Homo sapiens GN=ELAC2 PE=1 SV=2 - [RNZ2_HUMAN]**
**P07814**	**EPRS**	**glutamyl-prolyl-tRNA synthetase**
**Q9Y285**	**FARSA**	**Phenylalanyl-tRNA synthetase alpha chain OS=Homo sapiens GN=FARSA PE=1 SV=3 - [SYFA_HUMAN]**
**Q9NSD9**	**FARSB**	**Phenylalanyl-tRNA synthetase beta chain OS=Homo sapiens GN=FARSB PE=1 SV=3 - [SYFB_HUMAN]**
**Q96AE4**	**FUBP1**	**Far upstream element-binding protein 1 OS=Homo sapiens GN=FUBP1 PE=1 SV=3 - [FUBP1_HUMAN]**
**P41250**	**GARS**	**Glycyl-tRNA synthetase OS=Homo sapiens GN=GARS PE=1 SV=3 - [SYG_HUMAN]**
**P55084**	**HADHB**	**Trifunctional enzyme subunit beta, mitochondrial OS=Homo sapiens GN=HADHB PE=1 SV=3 - [ECHB_HUMAN]**
**P12081**	**HARS**	**Histidyl-tRNA synthetase, cytoplasmic OS=Homo sapiens GN=HARS PE=1 SV=2 - [SYHC_HUMAN]**
**P09651**	**HNRNPA1**	**heterogeneous nuclear ribonucleoprotein A1-like 3; similar to heterogeneous nuclear ribonucleoprotein A1; heterogeneous nuclear ribonucleoprotein A1 pseudogene 2; heterogeneous nuclear ribonucleoprotein A1; heterogeneous nuclear ribonucleoprotein A1 pseudogene**
**P22626**	**HNRNPA2B1**	**heterogeneous nuclear ribonucleoprotein A2/B1**
**Q99729**	**HNRNPAB**	**Heterogeneous nuclear ribonucleoprotein A/B OS=Homo sapiens GN=HNRNPAB PE=1 SV=2 - [ROAA_HUMAN]**
**Q14103**	**HNRNPD**	**heterogeneous nuclear ribonucleoprotein D (AU-rich element RNA binding protein 1, 37kDa)**
**P52597**	**HNRNPF**	**heterogeneous nuclear ribonucleoprotein F**
**P31943**	**HNRNPH1**	**Heterogeneous nuclear ribonucleoprotein H OS=Homo sapiens GN=HNRNPH1 PE=1 SV=4 - [HNRH1_HUMAN]**

**P31942**	**HNRNPH3**	**Heterogeneous nuclear ribonucleoprotein H3 OS=Homo sapiens GN=HNRNPH3 PE=1 SV=2 - [HNRH3_HUMAN]**
**P61978**	**HNRNPK**	**Heterogeneous nuclear ribonucleoprotein K OS=Homo sapiens GN=HNRNPK PE=1 SV=1 - [HNRPK_HUMAN]**
**P52272**	**HNRNPM**	**Heterogeneous nuclear ribonucleoprotein M OS=Homo sapiens GN=HNRNPM PE=1 SV=3 - [HNRPM_HUMAN]**
**Q00839**	**HNRNPU**	**Heterogeneous nuclear ribonucleoprotein U OS=Homo sapiens GN=HNRNPU PE=1 SV=6 - [HNRPU_HUMAN]**
**P07900**	**HSP90AA1**	**Heat shock protein HSP 90-alpha OS=Homo sapiens GN=HSP90AA1 PE=1 SV=5 - [HS90A_HUMAN]**
**P08238**	**HSP90AB1**	**heat shock protein 90kDa alpha (cytosolic), class B member 1**
**P11142**	**HSPA8**	**Heat shock cognate 71 kDa protein OS=Homo sapiens GN=HSPA8 PE=1 SV=1 - [HSP7C_HUMAN]**
**P41252**	**IARS**	**isoleucyl-tRNA synthetase**
**Q15046**	**KARS**	**lysyl-tRNA synthetase**
**Q92945**	**KHSRP**	**Far upstream element-binding protein 2 OS=Homo sapiens GN=KHSRP PE=1 SV=4 - [FUBP2_HUMAN]**
**Q9P2J5**	**LARS**	**leucyl-tRNA synthetase**
**Q32MZ4**	**LRRFIP1**	**Leucine-rich repeat flightless-interacting protein 1 OS=Homo sapiens GN=LRRFIP1 PE=1 SV=2 - [LRRF1_HUMAN]**
**P56192**	**MARS**	**Methionyl-tRNA synthetase, cytoplasmic OS=Homo sapiens GN=MARS PE=1 SV=2 - [SYMC_HUMAN]**
**O43776**	**Nars**	**Asparaginyl-tRNA synthetase, cytoplasmic OS=Homo sapiens GN=NARS PE=1 SV=1 - [SYNC_HUMAN]**
**P19338**	**NCL**	**nucleolin**
**P06748**	**NPM1**	**Nucleophosmin OS=Homo sapiens GN=NPM1 PE=1 SV=2 - [NPM_HUMAN]**
**O43809**	**NUDT21**	**Cleavage and polyadenylation specificity factor subunit 5 OS=Homo sapiens GN=NUDT21 PE=1 SV=1 - [CPSF5_HUMAN]**
**Q9UQ80**	**PA2G4**	**Proliferation-associated protein 2G4 OS=Homo sapiens GN=PA2G4 PE=1 SV=3 - [PA2G4_HUMAN]**
**Q15365**	**PCBP1**	**Poly(rC)-binding protein 1 OS=Homo sapiens GN=PCBP1 PE=1 SV=2 - [PCBP1_HUMAN]**
**Q9UMS4**	**PRPF19**	**PRP19/PSO4 pre-mRNA processing factor 19 homolog (S. cerevisiae)**
**P26599**	**PTBP1**	**polypyrimidine tract binding protein 1**
**P47897**	**QARS**	**Glutaminyl-tRNA synthetase OS=Homo sapiens GN=QARS PE=1 SV=1 - [SYQ_HUMAN]**
**P62826**	**RAN**	**RAN, member RAS oncogene family**
**P54136**	**RARS**	**arginyl-tRNA synthetase**
**Q96E39**	**RBMXL**	**Heterogeneous nuclear ribonucleoprotein G-like 1 OS=Homo sapiens GN=RBMXL1 PE=1 SV=1 - [RBMXL_HUMAN]**
**P13489**	**RNH1**	**Ribonuclease inhibitor OS=Homo sapiens GN=RNH1 PE=1 SV=2 - [RINI_HUMAN]**
**P30050**	**RPL12**	**60S ribosomal protein L12 OS=Homo sapiens GN=RPL12 PE=1 SV=1 - [RL12_HUMAN]**
**P35268**	**RPL22**	**60S ribosomal protein L22 OS=Homo sapiens GN=RPL22 PE=1 SV=2 - [RL22_HUMAN]**
**P62899**	**RPL31**	**ribosomal protein L31 pseudogene 49; ribosomal protein L31 pseudogene 17; ribosomal protein L31**
**P05388**	**rplP0**	**ribosomal protein, large, P0 pseudogene 2; ribosomal protein, large, P0 pseudogene 3; ribosomal protein, large, P0 pseudogene 6; ribosomal protein, large, P0**
**P05387**	**RPLP2**	**60S acidic ribosomal protein P2 OS=Homo sapiens GN=RPLP2 PE=1 SV=1 - [RLA2_HUMAN]**
**P46783**	**RPS10**	**40S ribosomal protein S10 OS=Homo sapiens GN=RPS10 PE=1 SV=1 - [RS10_HUMAN]**
**P62249**	**RPS16**	**40S ribosomal protein S16 OS=Homo sapiens GN=RPS16 PE=1 SV=2 - [RS16_HUMAN]**
**P39019**	**RPS19**	**40S ribosomal protein S19 OS=Homo sapiens GN=RPS19 PE=1 SV=2 - [RS19_HUMAN]**
**P08865**	**rpsA**	**40S ribosomal protein SA OS=Homo sapiens GN=RPSA PE=1 SV=4 - [RSSA_HUMAN]**
**Q9Y265**	**RUVBL1**	**RuvB-like 1 OS=Homo sapiens GN=RUVBL1 PE=1 SV=1 - [RUVB1_HUMAN]**

**Q8NC51**	**SERBP1**	**Plasminogen activator inhibitor 1 RNA-binding protein OS=Homo sapiens GN=SERBP1 PE=1 SV=2 - [PAIRB_HUMAN]**
**P23246**	**SFPQ**	**Splicing factor, proline- and glutamine-rich OS=Homo sapiens GN=SFPQ PE=1 SV=2 - [SFPQ_HUMAN]**
**Q9BXP5**	**SRRT**	**Serrate RNA effector molecule homolog OS=Homo sapiens GN=SRRT PE=1 SV=1 - [SRRT_HUMAN]**
**Q92804**	**TAF15**	**TATA-binding protein-associated factor 2N OS=Homo sapiens GN=TAF15 PE=1 SV=1 - [RBP56_HUMAN]**
**Q13148**	**TARDBP**	**TAR DNA binding protein**
**Q01085**	**TIAL1**	**TIA1 cytotoxic granule-associated RNA binding protein-like 1**
**Q9NXH9**	**TRMT1**	**N(2),N(2)-dimethylguanosine tRNA methyltransferase OS=Homo sapiens GN=TRMT1 PE=1 SV=1 - [TRM1_HUMAN]**
**P49411**	**TUFM**	**Tu translation elongation factor, mitochondrial**
**Q9BYJ9**	**YTHDF1**	**YTH domain family protein 1 OS=Homo sapiens GN=YTHDF1 PE=1 SV=1 - [YTHD1_HUMAN]**
**Q9Y5A9**	**YTHDF2**	**YTH domain family protein 2 OS=Homo sapiens GN=YTHDF2 PE=1 SV=2 - [YTHD2_HUMAN]**
**Q7Z739**	**YTHDF3**	**YTH domain family protein 3 OS=Homo sapiens GN=YTHDF3 PE=1 SV=1 - [YTHD3_HUMAN]**
P60709	ACTB	actin, beta
O95994	AGR2	Anterior gradient protein 2 homolog OS=Homo sapiens GN=AGR2 PE=1 SV=1 - [AGR2_HUMAN]
O95831	AIFM1	Apoptosis-inducing factor 1, mitochondrial OS=Homo sapiens GN=AIFM1 PE=1 SV=1 - [AIFM1_HUMAN]
Q13155	AIMP2	aminoacyl tRNA synthetase complex-interacting multifunctional protein 2; stromal antigen 3-like 3
P42330	AKR1C3	Aldo-keto reductase family 1 member C3 OS=Homo sapiens GN=AKR1C3 PE=1 SV=4 - [AK1C3_HUMAN]
P02768	ALB	Serum albumin OS=Homo sapiens GN=ALB PE=1 SV=2 - [ALBU_HUMAN]
P09972	ALDOC	Fructose-bisphosphate aldolase C OS=Homo sapiens GN=ALDOC PE=1 SV=2 - [ALDOC_HUMAN]
P04083	ANXA1	Annexin A1 OS=Homo sapiens GN=ANXA1 PE=1 SV=2 - [ANXA1_HUMAN]
P50995	ANXA11	Annexin A11 OS=Homo sapiens GN=ANXA11 PE=1 SV=1 - [ANX11_HUMAN]
P20073	ANXA7	Annexin A7 OS=Homo sapiens GN=ANXA7 PE=1 SV=3 - [ANXA7_HUMAN]
P61204	ARF3	ADP-ribosylation factor 3 OS=Homo sapiens GN=ARF3 PE=1 SV=2 - [ARF3_HUMAN]
P46379	BAT3	Large proline-rich protein BAT3 OS=Homo sapiens GN=BAT3 PE=1 SV=2 - [BAT3_HUMAN]
Q7L1Q6	BZW1	Basic leucine zipper and W2 domain-containing protein 1 OS=Homo sapiens GN=BZW1 PE=1 SV=1 - [BZW1_HUMAN]
Q9Y6E2	BZW2	Basic leucine zipper and W2 domain-containing protein 2 OS=Homo sapiens GN=BZW2 PE=1 SV=1 - [BZW2_HUMAN]
Q9HB71	CACYBP	Calcyclin-binding protein OS=Homo sapiens GN=CACYBP PE=1 SV=2 - [CYBP_HUMAN]
P27797	CALR	Calreticulin OS=Homo sapiens GN=CALR PE=1 SV=1 - [CALR_HUMAN]
P49368	CCT3	T-complex protein 1 subunit gamma OS=Homo sapiens GN=CCT3 PE=1 SV=4 - [TCPG_HUMAN]
P48643	CCT5	chaperonin containing TCP1, subunit 5 (epsilon)
P40227	CCT6A	T-complex protein 1 subunit zeta OS=Homo sapiens GN=CCT6A PE=1 SV=3 - [TCPZ_HUMAN]
Q99832	CCT7	T-complex protein 1 subunit eta OS=Homo sapiens GN=CCT7 PE=1 SV=2 - [TCPH_HUMAN]
P01040	CSTA	Cystatin-A OS=Homo sapiens GN=CSTA PE=1 SV=1 - [CYTA_HUMAN]
P81605	DCD	Dermcidin OS=Homo sapiens GN=DCD PE=1 SV=2 - [DCD_HUMAN]
Q14204	DYNC1H1	Cytoplasmic dynein 1 heavy chain 1 OS=Homo sapiens GN=DYNC1H1 PE=1 SV=5 - [DYHC1_HUMAN]
P26641	EEF1G	Elongation factor 1-gamma OS=Homo sapiens GN=EEF1G PE=1 SV=3 - [EF1G_HUMAN]
P13804	ETFA	Electron transfer flavoprotein subunit alpha, mitochondrial OS=Homo sapiens GN=ETFA PE=1 SV=1 - [ETFA_HUMAN]

P15311	EZR	Ezrin OS=Homo sapiens GN=EZR PE=1 SV=4 - [EZRI_HUMAN]
Q96TA1	FAM129B	Niban-like protein 1 OS=Homo sapiens GN=FAM129B PE=1 SV=3 - [NIBL1_HUMAN]
Q00688	FKBP3	Peptidyl-prolyl cis-trans isomerase FKBP3 OS=Homo sapiens GN=FKBP3 PE=1 SV=1 - [FKBP3_HUMAN]
Q16658	FSCN1	fascin homolog 1, actin-bundling protein (Strongylocentrotus purpuratus)
P04899	GNAI2	Guanine nucleotide-binding protein G(i) subunit alpha-2 OS=Homo sapiens GN=GNAI2 PE=1 SV=3 - [GNAI2_HUMAN]
P62873	GNB1	guanine nucleotide binding protein (G protein), beta polypeptide 1
P69905	HBA1	Hemoglobin subunit alpha OS=Homo sapiens GN=HBA1 PE=1 SV=2 - [HBA_HUMAN]
P16403	HIST1H1C	Histone H1.2 OS=Homo sapiens GN=HIST1H1C PE=1 SV=2 - [H12_HUMAN]
Q96KK5	HIST1H2AH	Histone H2A type 1-H OS=Homo sapiens GN=HIST1H2AH PE=1 SV=3 - [H2A1H_HUMAN]
B2RPK0	HMGB1L1	Putative high mobility group protein B1-like 1 OS=Homo sapiens GN=HMGB1L1 PE=5 SV=1 - [HGB1A_HUMAN]
P08107	HSPA1A	Heat shock 70 kDa protein 1A/1B OS=Homo sapiens GN=HSPA1A PE=1 SV=5 - [HSP71_HUMAN]
P34932	HSPA4	Heat shock 70 kDa protein 4 OS=Homo sapiens GN=HSPA4 PE=1 SV=4 - [HSP74_HUMAN]
P11021	HSPA5	78 kDa glucose-regulated protein OS=Homo sapiens GN=HSPA5 PE=1 SV=2 - [GRP78_HUMAN]
P17066	HSPA6	heat shock 70kDa protein 7 (HSP70B); heat shock 70kDa protein 6 (HSP70B')
P38646	HSPA9	Stress-70 protein, mitochondrial OS=Homo sapiens GN=HSPA9 PE=1 SV=2 - [GRP75_HUMAN]
P04792	HSPB1	Heat shock protein beta-1 OS=Homo sapiens GN=HSPB1 PE=1 SV=2 - [HSPB1_HUMAN]
P48735	IDH2	Isocitrate dehydrogenase [NADP], mitochondrial OS=Homo sapiens GN=IDH2 PE=1 SV=2 - [IDHP_HUMAN]
P12268	IMPDH2	Inosine-5'-monophosphate dehydrogenase 2 OS=Homo sapiens GN=IMPDH2 PE=1 SV=2 - [IMDH2_HUMAN]
Q14974	KPNB1	Importin subunit beta-1 OS=Homo sapiens GN=KPNB1 PE=1 SV=2 - [IMB1_HUMAN]
P43490	NAMPT	nicotinamide phosphoribosyltransferase
Q00653	NFKB2	Nuclear factor NF-kappa-B p100 subunit OS=Homo sapiens GN=NFKB2 PE=1 SV=4 - [NFKB2_HUMAN]
P22392	NME2	non-metastatic cells 1, protein (NM23A) expressed in; NME1-NME2 readthrough transcript; non-metastatic cells 2, protein (NM23B) expressed in
P22234	PAICS	Multifunctional protein ADE2 OS=Homo sapiens GN=PAICS PE=1 SV=3 - [PUR6_HUMAN]
P05166	PCCB	Propionyl-CoA carboxylase beta chain, mitochondrial OS=Homo sapiens GN=PCCB PE=1 SV=3 - [PCCB_HUMAN]
Q15084	PDIA6	Protein disulfide-isomerase A6 OS=Homo sapiens GN=PDIA6 PE=1 SV=1 - [PDIA6_HUMAN]
Q96HC4	PDLIM5	PDZ and LIM domain protein 5 OS=Homo sapiens GN=PDLIM5 PE=1 SV=5 - [PDLI5_HUMAN]
P52209	PGD	6-phosphogluconate dehydrogenase, decarboxylating OS=Homo sapiens GN=PGD PE=1 SV=3 - [6PGD_HUMAN]
Q99623	PHB2	Prohibitin-2 OS=Homo sapiens GN=PHB2 PE=1 SV=2 - [PHB2_HUMAN]
O43175	PHGDH	D-3-phosphoglycerate dehydrogenase OS=Homo sapiens GN=PHGDH PE=1 SV=4 - [SERA_HUMAN]
P30041	PRDX6	Peroxiredoxin-6 OS=Homo sapiens GN=PRDX6 PE=1 SV=3 - [PRDX6_HUMAN]
P78527	PRKDC	similar to protein kinase, DNA-activated, catalytic polypeptide; protein kinase, DNA-activated, catalytic polypeptide
P61026	RAB10	Ras-related protein Rab-10 OS=Homo sapiens GN=RAB10 PE=1 SV=1 - [RAB10_HUMAN]
P62491	RAB11A	Ras-related protein Rab-11A OS=Homo sapiens GN=RAB11A PE=1 SV=3 - [RB11A_HUMAN]
P61019	RAB2A	Ras-related protein Rab-2A OS=Homo sapiens GN=RAB2A PE=1 SV=1 - [RAB2A_HUMAN]
P61020	RAB5B	Ras-related protein Rab-5B OS=Homo sapiens GN=RAB5B PE=1 SV=1 - [RAB5B_HUMAN]
P51148	RAB5C	Ras-related protein Rab-5C OS=Homo sapiens GN=RAB5C PE=1 SV=2 -

		[RAB5C_HUMAN]
Q9Y230	RUVBL2	RuvB-like 2 (E. coli)
P31949	S100A11	Protein S100-A11 OS=Homo sapiens GN=S100A11 PE=1 SV=2 - [S10AB_HUMAN]
P06703	S100A6	Protein S100-A6 OS=Homo sapiens GN=S100A6 PE=1 SV=1 - [S10A6_HUMAN]
Q15436	SEC23A	Protein transport protein Sec23A OS=Homo sapiens GN=SEC23A PE=1 SV=2 - [SC23A_HUMAN]
Q15019	SEPT2	Septin-2 OS=Homo sapiens GN=SEPT2 PE=1 SV=1 - [SEPT2_HUMAN]
Q16181	SEPT7	Septin-7 OS=Homo sapiens GN=SEPT7 PE=1 SV=2 - [SEPT7_HUMAN]
Q9UHD8	SEPT9	Septin-9 OS=Homo sapiens GN=SEPT9 PE=1 SV=2 - [SEPT9_HUMAN]
Q01105	SET	Protein SET OS=Homo sapiens GN=SET PE=1 SV=3 - [SET_HUMAN]
Q04837	SSBP1	single-stranded DNA binding protein 1
Q9UJZ1	STOML2	Stomatin-like protein 2 OS=Homo sapiens GN=STOML2 PE=1 SV=1 - [STML2_HUMAN]
P53999	SUB1	SUB1 homolog (S. cerevisiae)
P17987	TCP1	T-complex protein 1 subunit alpha OS=Homo sapiens GN=TCP1 PE=1 SV=1 - [TCPA_HUMAN]
P29401	TKT	Transketolase OS=Homo sapiens GN=TKT PE=1 SV=3 - [TKT_HUMAN]
P49755	TMED10	Transmembrane emp24 domain-containing protein 10 OS=Homo sapiens GN=TMED10 PE=1 SV=2 - [TMEDA_HUMAN]
Q12931	TRAP1	Heat shock protein 75 kDa, mitochondrial OS=Homo sapiens GN=TRAP1 PE=1 SV=3 - [TRAP1_HUMAN]
Q13263	TRIM28	Transcription intermediary factor 1-beta OS=Homo sapiens GN=TRIM28 PE=1 SV=5 - [TIF1B_HUMAN]
P10599	TXN	Thioredoxin OS=Homo sapiens GN=TXN PE=1 SV=3 - [THIO_HUMAN]
Q14157	UBAP2L	Ubiquitin-associated protein 2-like OS=Homo sapiens GN=UBAP2L PE=1 SV=2 - [UBP2L_HUMAN]
P0CG47	UBB	Polyubiquitin-B OS=Homo sapiens GN=UBB PE=1 SV=1 - [UBB_HUMAN]
P11441	UBL4A	Ubiquitin-like protein 4A OS=Homo sapiens GN=UBL4A PE=1 SV=1 - [UBL4A_HUMAN]
Q9UHD9	UBQLN2	Ubiquilin-2 OS=Homo sapiens GN=UBQLN2 PE=1 SV=2 - [UBQL2_HUMAN]
P13010	XRCC5	X-ray repair cross-complementing protein 5 OS=Homo sapiens GN=XRCC5 PE=1 SV=3 - [XRCC5_HUMAN]
P12956	XRCC6	X-ray repair complementing defective repair in Chinese hamster cells 6; similar to ATP-dependent DNA helicase II, 70 kDa subunit
P62258	YWHAE	14-3-3 protein epsilon OS=Homo sapiens GN=YWHAE PE=1 SV=1 - [1433E_HUMAN]
**Proteins identified in this assay, but in common with proteins in the negative control**		
O60701	UGDH	UDP-glucose 6-dehydrogenase OS=Homo sapiens GN=UGDH PE=1 SV=1 - [UGDH_HUMAN]
P02545	LMNA	Prelamin-A/C OS=Homo sapiens GN=LMNA PE=1 SV=1 - [LMNA_HUMAN]
P04350	TUBB4	tubulin, beta 4
P04406	GAPDH	Glyceraldehyde-3-phosphate dehydrogenase OS=Homo sapiens GN=GAPDH PE=1 SV=3 - [G3P_HUMAN]
P06576	ATP5B	ATP synthase, H+ transporting, mitochondrial F1 complex, beta polypeptide
P07355	ANXA2	Annexin A2 OS=Homo sapiens GN=ANXA2 PE=1 SV=2 - [ANXA2_HUMAN]
P08758	ANXA5	annexin A5
P0CG48	UBC	Polyubiquitin-C
P12236	SLC25A6	solute carrier family 25 (mitochondrial carrier; adenine nucleotide translocator), member 6
P02794	FTH1	Ferritin heavy chain OS=Homo sapiens GN=FTH1 PE=1 SV=2 - [FRIH_HUMAN]
P15559	NQO1	NAD(P)H dehydrogenase, quinone 1
P20337	RAB3B	RAB3B, member RAS oncogene family
P21333	FLNA	filamin A, alpha (actin binding protein 280)
P25705	ATP5A1	ATP synthase, H+ transporting, mitochondrial F1 complex, alpha subunit 1, cardiac muscle

P30048	PRDX3	peroxiredoxin 3
P30101	PDIA3	Protein disulfide-isomerase A3 OS=Homo sapiens GN=PDIA3 PE=1 SV=4 - [PDIA3_HUMAN]
P31327	Cps1	carbamoyl-phosphate synthetase 1, mitochondrial
P34897	SHMT2	serine hydroxymethyltransferase 2 (mitochondrial)
P35579	MYH9	Myosin-9 OS=Homo sapiens GN=MYH9 PE=1 SV=4 - [MYH9_HUMAN]
P35580	MYH10	myosin, heavy chain 10, non-muscle
P02771	AFP	Alpha-fetoprotein OS=Homo sapiens GN=AFP PE=1 SV=1 - [FETA_HUMAN]
P62820	RAB1A	RAB1A, member RAS oncogene family
P62937	PPIA	similar to TRIMCyp; peptidylprolyl isomerase A (cyclophilin A); peptidylprolyl isomerase A (cyclophilin A)-like 3
P63104	YWHAZ	tyrosine 3-monooxygenase/tryptophan 5-monooxygenase activation protein, zeta polypeptide
P68032	ACTC1	actin, alpha, cardiac muscle 1
Q00325	SLC25A3	solute carrier family 25 (mitochondrial carrier; phosphate carrier), member 3
Q00610	CLTC	clathrin, heavy chain (Hc)
Q01082	SPTBN1	spectrin, beta, non-erythrocytic 1
Q13492	PICALM	phosphatidylinositol binding clathrin assembly protein
Q13813	SPTAN1	spectrin, alpha, non-erythrocytic 1 (alpha-fodrin)
Q96M27	PRRC1	proline-rich coiled-coil 1
Q99714	HSD17B10	hydroxysteroid (17-beta) dehydrogenase 10
Q9NR31	SAR1A	SAR1 homolog A (S. cerevisiae)
