# Supplementary material for: Identification of RNA-binding proteins in exosomes capable of interacting with different types of RNA: RBP-facilitated transport of RNAs into exosomes
Source: PLoS One. 2018 Apr 24;13(4):e0195969. doi: 10.1371/journal.pone.0195969 (PMC5918169; doi:10.1371/journal.pone.0195969)
Supplement: S6 Table — In total, 238 proteins were identified of which 83 proteins were RBPs (bold) according to the GO terms. Proteins in common with negative controls (56 proteins) are listed separately below. None of the proteins present in the negative control were RBPs. (PDF) [file pone.0195969.s012.pdf]

S6 Table. All proteins identified in the assay with cell: "Cellular proteins + Cellular mRNA". In total, 238 proteins were identified of which 83 proteins were RBPs (bold) according to the GO terms. Proteins in common with negative controls (56 proteins) are listed separately below. None of the proteins present in the negative control were RBPs.

Accession	Gene name	Description
**P54886**	**ALDH18A1**	**Delta-1-pyrroline-5-carboxylate synthase OS=Homo sapiens GN=ALDH18A1 PE=1 SV=2 - [P5CS_HUMAN]**
**Q92499**	**DDX1**	**ATP-dependent RNA helicase DDX1 OS=Homo sapiens GN=DDX1 PE=1 SV=2 - [DDX1_HUMAN]**
**O00571**	**DDX3X**	**ATP-dependent RNA helicase DDX3X OS=Homo sapiens GN=DDX3X PE=1 SV=3 - [DDX3X_HUMAN]**
**P17844**	**DDX5**	**Probable ATP-dependent RNA helicase DDX5 OS=Homo sapiens GN=DDX5 PE=1 SV=1 - [DDX5_HUMAN]**
**Q08211**	**DHX9**	**ATP-dependent RNA helicase A OS=Homo sapiens GN=DHX9 PE=1 SV=4 - [DHX9_HUMAN]**
**P68104**	**EEF1A1**	**Elongation factor 1-alpha 1 OS=Homo sapiens GN=EEF1A1 PE=1 SV=1 - [EF1A1_HUMAN]**
**P29692**	**EEF1D**	**Elongation factor 1-delta OS=Homo sapiens GN=EEF1D PE=1 SV=5 - [EF1D_HUMAN]**
**P13639**	**EEF2**	**Elongation factor 2 OS=Homo sapiens GN=EEF2 PE=1 SV=4 - [EF2_HUMAN]**
**P60842**	**EIF4A1**	**Eukaryotic initiation factor 4A-I OS=Homo sapiens GN=EIF4A1 PE=1 SV=1 - [IF4A1_HUMAN]**
**P38919**	**EIF4A3**	**Eukaryotic initiation factor 4A-III OS=Homo sapiens GN=EIF4A3 PE=1 SV=4 - [IF4A3_HUMAN]**
**Q9Y285**	**FARSA**	**Phenylalanine--tRNA ligase alpha subunit OS=Homo sapiens GN=FARSA PE=1 SV=3 - [SYFA_HUMAN]**
**Q9NSD9**	**FARSB**	**Phenylalanine--tRNA ligase beta subunit OS=Homo sapiens GN=FARSB PE=1 SV=3 - [SYFB_HUMAN]**
**P22087**	**FBL**	**rRNA 2'-O-methyltransferase fibrillarin OS=Homo sapiens GN=FBL PE=1 SV=2 - [FBRL_HUMAN]**
**Q96AE4**	**FUBP1**	**Far upstream element-binding protein 1 OS=Homo sapiens GN=FUBP1 PE=1 SV=3 - [FUBP1_HUMAN]**
**Q92616**	**GCN1L1**	**Translational activator GCN1 OS=Homo sapiens GN=GCN1L1 PE=1 SV=6 - [GCN1L_HUMAN]**
**P09651**	**HNRNPA1**	**Heterogeneous nuclear ribonucleoprotein A1 OS=Homo sapiens GN=HNrnpA1 PE=1 SV=5 - [ROA1_HUMAN]**
**P22626**	**HNRNPA2B1**	**Heterogeneous nuclear ribonucleoproteins A2/B1 OS=Homo sapiens GN=HNrnpA2B1 PE=1 SV=2 - [ROA2_HUMAN]**
**P07910**	**HNRNPC**	**Heterogeneous nuclear ribonucleoproteins C1/C2 OS=Homo sapiens GN=HNrnpC PE=1 SV=4 - [HNRPC_HUMAN]**
**Q14103**	**HNRNPD**	**Heterogeneous nuclear ribonucleoprotein D0 OS=Homo sapiens GN=HNrnpD PE=1 SV=1 - [HNRPD_HUMAN]**
**P31943**	**HNRNPH1**	**Heterogeneous nuclear ribonucleoprotein H OS=Homo sapiens GN=HNrnpH1 PE=1 SV=4 - [HNRH1_HUMAN]**
**P31942**	**HNRNPH3**	**Heterogeneous nuclear ribonucleoprotein H3 OS=Homo sapiens GN=HNrnpH3 PE=1 SV=2 - [HNRH3_HUMAN]**
**P61978**	**HNRNPK**	**Heterogeneous nuclear ribonucleoprotein K OS=Homo sapiens GN=HNrnpK PE=1 SV=1 - [HNRPK_HUMAN]**

**P52272**	**HNRNPM**	**Heterogeneous nuclear ribonucleoprotein M OS=Homo sapiens GN=HNrnpM PE=1 SV=3 - [HNRPM_HUMAN]**
**Q00839**	**HNRNPU**	**Heterogeneous nuclear ribonucleoprotein U OS=Homo sapiens GN=HNrnpU PE=1 SV=6 - [HNRPU_HUMAN]**
**P07900**	**HSP90AA1**	**Heat shock protein HSP 90-alpha OS=Homo sapiens GN=HSP90AA1 PE=1 SV=5 - [HS90A_HUMAN]**
**P08238**	**HSP90AB1**	**Heat shock protein HSP 90-beta OS=Homo sapiens GN=HSP90AB1 PE=1 SV=4 - [HS90B_HUMAN]**
**P11142**	**HSPA8**	**Heat shock cognate 71 kDa protein OS=Homo sapiens GN=HSPA8 PE=1 SV=1 - [HSP7C_HUMAN]**
**Q9NSE4**	**IARS2**	**Isoleucine--tRNA ligase, mitochondrial OS=Homo sapiens GN=IARS2 PE=1 SV=2 - [SYIM_HUMAN]**
**Q16891**	**IMMT**	**Mitochondrial inner membrane protein OS=Homo sapiens GN=IMMT PE=1 SV=1 - [IMMT_HUMAN]**
**Q9P2J5**	**LARS**	**Leucine--tRNA ligase, cytoplasmic OS=Homo sapiens GN=LARS PE=1 SV=2 - [SYLC_HUMAN]**
**P42704**	**LRPPRC**	**Leucine-rich PPR motif-containing protein, mitochondrial OS=Homo sapiens GN=LRPPRC PE=1 SV=3 - [LPPRC_HUMAN]**
**P56192**	**MARS**	**Methionine--tRNA ligase, cytoplasmic OS=Homo sapiens GN=MARS PE=1 SV=2 - [SYMC_HUMAN]**
**Q14764**	**MVP**	**Major vault protein OS=Homo sapiens GN=MVP PE=1 SV=4 - [MVP_HUMAN]**
**P19338**	**NCL**	**Nucleolin OS=Homo sapiens GN=NCL PE=1 SV=3 - [NUCL_HUMAN]**
**Q08J23**	**NSUN2**	**tRNA (cytosine(34)-C(5))-methyltransferase OS=Homo sapiens GN=NSUN2 PE=1 SV=2 - [NSUN2_HUMAN]**
**Q92621**	**NUP205**	**Nuclear pore complex protein Nup205 OS=Homo sapiens GN=NUP205 PE=1 SV=3 - [NU205_HUMAN]**
**Q15365**	**PCBP1**	**Poly(rC)-binding protein 1 OS=Homo sapiens GN=PCBP1 PE=1 SV=2 - [PCBP1_HUMAN]**
**Q6P2Q9**	**PRPF8**	**Pre-mRNA-processing-splicing factor 8 OS=Homo sapiens GN=PRPF8 PE=1 SV=2 - [PRP8_HUMAN]**
**P26599**	**PTBP1**	**Polypyrimidine tract-binding protein 1 OS=Homo sapiens GN=PTBP1 PE=1 SV=1 - [PTBP1_HUMAN]**
**Q9UKM9**	**RALY**	**RNA-binding protein Raly OS=Homo sapiens GN=RALY PE=1 SV=1 - [RALY_HUMAN]**
**P62826**	**RAN**	**GTP-binding nuclear protein Ran OS=Homo sapiens GN=RAN PE=1 SV=3 - [RAN_HUMAN]**
**P13489**	**RNH1**	**Ribonuclease inhibitor OS=Homo sapiens GN=RNH1 PE=1 SV=2 - [RINI_HUMAN]**
**P27635**	**RPL10**	**60S ribosomal protein L10 OS=Homo sapiens GN=RPL10 PE=1 SV=4 - [RL10_HUMAN]**
**P62906**	**RPL10A**	**60S ribosomal protein L10a OS=Homo sapiens GN=RPL10A PE=1 SV=2 - [RL10A_HUMAN]**
**P62913**	**RPL11**	**60S ribosomal protein L11 OS=Homo sapiens GN=RPL11 PE=1 SV=2 - [RL11_HUMAN]**
**P30050**	**RPL12**	**60S ribosomal protein L12 OS=Homo sapiens GN=RPL12 PE=1 SV=1 - [RL12_HUMAN]**
**P26373**	**RPL13**	**60S ribosomal protein L13 OS=Homo sapiens GN=RPL13 PE=1 SV=4 - [RL13_HUMAN]**
**P50914**	**RPL14**	**60S ribosomal protein L14 OS=Homo sapiens GN=RPL14 PE=1 SV=4 - [RL14_HUMAN]**
**P18621**	**RPL17**	**60S ribosomal protein L17 OS=Homo sapiens GN=RPL17 PE=1 SV=3 - [RL17_HUMAN]**
**Q07020**	**RPL18**	**60S ribosomal protein L18 OS=Homo sapiens GN=RPL18 PE=1 SV=2 - [RL18_HUMAN]**
**P46778**	**RPL21**	**60S ribosomal protein L21 OS=Homo sapiens GN=RPL21 PE=1 SV=2 - [RL21_HUMAN]**
**P62829**	**RPL23**	**60S ribosomal protein L23 OS=Homo sapiens GN=RPL23 PE=1 SV=1 - [RL23_HUMAN]**

**P83731**	**RPL24**	**60S ribosomal protein L24 OS=Homo sapiens GN=RPL24 PE=1 SV=1 - [RL24_HUMAN]**
**P61353**	**RPL27**	**60S ribosomal protein L27 OS=Homo sapiens GN=RPL27 PE=1 SV=2 - [RL27_HUMAN]**
**P46779**	**RPL28**	**60S ribosomal protein L28 OS=Homo sapiens GN=RPL28 PE=1 SV=3 - [RL28_HUMAN]**
**P39023**	**RPL3**	**60S ribosomal protein L3 OS=Homo sapiens GN=RPL3 PE=1 SV=2 - [RL3_HUMAN]**
**P18077**	**RPL35A**	**60S ribosomal protein L35a OS=Homo sapiens GN=RPL35A PE=1 SV=2 - [RL35A_HUMAN]**
**Q9Y3U8**	**RPL36**	**60S ribosomal protein L36 OS=Homo sapiens GN=RPL36 PE=1 SV=3 - [RL36_HUMAN]**
**P36578**	**RPL4**	**60S ribosomal protein L4 OS=Homo sapiens GN=RPL4 PE=1 SV=5 - [RL4_HUMAN]**
**Q02878**	**RPL6**	**60S ribosomal protein L6 OS=Homo sapiens GN=RPL6 PE=1 SV=3 - [RL6_HUMAN]**
**P62917**	**RPL8**	**60S ribosomal protein L8 OS=Homo sapiens GN=RPL8 PE=1 SV=2 - [RL8_HUMAN]**
**P05388**	**RPLP0**	**60S acidic ribosomal protein P0 OS=Homo sapiens GN=RPLP0 PE=1 SV=1 - [RLA0_HUMAN]**
**P62277**	**RPS13**	**40S ribosomal protein S13 OS=Homo sapiens GN=RPS13 PE=1 SV=2 - [RS13_HUMAN]**
**P62244**	**RPS15A**	**40S ribosomal protein S15a OS=Homo sapiens GN=RPS15A PE=1 SV=2 - [RS15A_HUMAN]**
**P62249**	**RPS16**	**40S ribosomal protein S16 OS=Homo sapiens GN=RPS16 PE=1 SV=2 - [RS16_HUMAN]**
**P0CW22**	**RPS17L**	**40S ribosomal protein S17-like OS=Homo sapiens GN=RPS17L PE=1 SV=1 - [RS17L_HUMAN]**
**P62266**	**RPS23**	**40S ribosomal protein S23 OS=Homo sapiens GN=RPS23 PE=1 SV=3 - [RS23_HUMAN]**
**P62851**	**RPS25**	**40S ribosomal protein S25 OS=Homo sapiens GN=RPS25 PE=1 SV=1 - [RS25_HUMAN]**
**P62854**	**RPS26**	**40S ribosomal protein S26 OS=Homo sapiens GN=RPS26 PE=1 SV=3 - [RS26_HUMAN]**
**P46782**	**RPS5**	**40S ribosomal protein S5 OS=Homo sapiens GN=RPS5 PE=1 SV=4 - [RS5_HUMAN]**
**P62753**	**RPS6**	**40S ribosomal protein S6 OS=Homo sapiens GN=RPS6 PE=1 SV=1 - [RS6_HUMAN]**
**P62081**	**RPS7**	**40S ribosomal protein S7 OS=Homo sapiens GN=RPS7 PE=1 SV=1 - [RS7_HUMAN]**
**P62241**	**RPS8**	**40S ribosomal protein S8 OS=Homo sapiens GN=RPS8 PE=1 SV=2 - [RS8_HUMAN]**
**P46781**	**RPS9**	**40S ribosomal protein S9 OS=Homo sapiens GN=RPS9 PE=1 SV=3 - [RS9_HUMAN]**
**Q5JTH9**	**RRP12**	**RRP12-like protein OS=Homo sapiens GN=RRP12 PE=1 SV=2 - [RRP12_HUMAN]**
**Q9Y3I0**	**RTCB**	**tRNA-splicing ligase RtcB homolog OS=Homo sapiens GN=RTCB PE=1 SV=1 - [RTCB_HUMAN]**
**Q9Y265**	**RUVBL1**	**RuvB-like 1 OS=Homo sapiens GN=RUVBL1 PE=1 SV=1 - [RUVB1_HUMAN]**
**P23246**	**SFPQ**	**Splicing factor, proline- and glutamine-rich OS=Homo sapiens GN=SFPQ PE=1 SV=2 - [SFPQ_HUMAN]**
**O15427**	**SLC16A3**	**Monocarboxylate transporter 4 OS=Homo sapiens GN=SLC16A3 PE=1 SV=1 - [MOT4_HUMAN]**
**P08195**	**SLC3A2**	**4F2 cell-surface antigen heavy chain OS=Homo sapiens GN=SLC3A2 PE=1 SV=3 - [4F2_HUMAN]**
**P02786**	**TFRC**	**Transferrin receptor protein 1 OS=Homo sapiens GN=TFRC PE=1 SV=2 - [TFR1_HUMAN]**
**P49411**	**TUFM**	**Elongation factor Tu, mitochondrial OS=Homo sapiens GN=TUFM PE=1**

		**SV=2 - [EFTU_HUMAN]**
P28288	ABCD3	ATP-binding cassette sub-family D member 3 OS=Homo sapiens GN=ABCD3 PE=1 SV=1 - [ABCD3_HUMAN]
P61221	ABCE1	ATP-binding cassette sub-family E member 1 OS=Homo sapiens GN=ABCE1 PE=1 SV=1 - [ABCE1_HUMAN]
P11310	ACADM	Medium-chain specific acyl-CoA dehydrogenase, mitochondrial OS=Homo sapiens GN=ACADM PE=1 SV=1 - [ACADM_HUMAN]
P45954	ACADSB	Short/branched chain specific acyl-CoA dehydrogenase, mitochondrial OS=Homo sapiens GN=ACADSB PE=1 SV=1 - [ACDSB_HUMAN]
Q9Y305	ACOT9	Acyl-coenzyme A thioesterase 9, mitochondrial OS=Homo sapiens GN=ACOT9 PE=1 SV=2 - [ACOT9_HUMAN]
P33121	ACSL1	Long-chain-fatty-acid--CoA ligase 1 OS=Homo sapiens GN=ACSL1 PE=1 SV=1 - [ACSL1_HUMAN]
P42025	ACTR1B	Beta-centractin OS=Homo sapiens GN=ACTR1B PE=1 SV=1 - [ACTY_HUMAN]
Q9Y4W6	AFG3L2	AFG3-like protein 2 OS=Homo sapiens GN=AFG3L2 PE=1 SV=2 - [AFG32_HUMAN]
O95433	AHSA1	Activator of 90 kDa heat shock protein ATPase homolog 1 OS=Homo sapiens GN=AHSA1 PE=1 SV=1 - [AHSA1_HUMAN]
P51648	ALDH3A2	Fatty aldehyde dehydrogenase OS=Homo sapiens GN=ALDH3A2 PE=1 SV=1 - [AL3A2_HUMAN]
P04083	ANXA1	Annexin A1 OS=Homo sapiens GN=ANXA1 PE=1 SV=2 - [ANXA1_HUMAN]
P50995	ANXA11	Annexin A11 OS=Homo sapiens GN=ANXA11 PE=1 SV=1 - [ANX11_HUMAN]
P20073	ANXA7	Annexin A7 OS=Homo sapiens GN=ANXA7 PE=1 SV=3 - [ANXA7_HUMAN]
Q9BQE5	APOL2	Apolipoprotein L2 OS=Homo sapiens GN=APOL2 PE=1 SV=1 - [APOL2_HUMAN]
P61204	ARF3	ADP-ribosylation factor 3 OS=Homo sapiens GN=ARF3 PE=1 SV=2 - [ARF3_HUMAN]
Q9NVJ2	ARL8B	ADP-ribosylation factor-like protein 8B OS=Homo sapiens GN=ARL8B PE=1 SV=1 - [ARL8B_HUMAN]
Q12797	ASPH	Aspartyl/asparaginyl beta-hydroxylase OS=Homo sapiens GN=ASPH PE=1 SV=3 - [ASPH_HUMAN]
Q9NVI7	ATAD3A	ATPase family AAA domain-containing protein 3A OS=Homo sapiens GN=ATAD3A PE=1 SV=2 - [ATD3A_HUMAN]
P05023	ATP1A1	Sodium/potassium-transporting ATPase subunit alpha-1 OS=Homo sapiens GN=ATP1A1 PE=1 SV=1 - [AT1A1_HUMAN]
P20020	ATP2B1	Plasma membrane calcium-transporting ATPase 1 OS=Homo sapiens GN=ATP2B1 PE=1 SV=3 - [AT2B1_HUMAN]
P24539	ATP5F1	ATP synthase subunit b, mitochondrial OS=Homo sapiens GN=ATP5F1 PE=1 SV=2 - [AT5F1_HUMAN]
O75947	ATP5H	ATP synthase subunit d, mitochondrial OS=Homo sapiens GN=ATP5H PE=1 SV=3 - [ATP5H_HUMAN]
P48047	ATP5O	ATP synthase subunit O, mitochondrial OS=Homo sapiens GN=ATP5O PE=1 SV=1 - [ATPO_HUMAN]
O95816	BAG2	BAG family molecular chaperone regulator 2 OS=Homo sapiens GN=BAG2 PE=1 SV=1 - [BAG2_HUMAN]
P35613	BSG	Basigin OS=Homo sapiens GN=BSG PE=1 SV=2 - [BASI_HUMAN]
Q7L1Q6	BZW1	Basic leucine zipper and W2 domain-containing protein 1 OS=Homo sapiens GN=BZW1 PE=1 SV=1 - [BZW1_HUMAN]
Q96GQ5	C16orf58	UPF0420 protein C16orf58 OS=Homo sapiens GN=C16orf58 PE=1 SV=2 - [CP058_HUMAN]
Q9BQA9	C17orf62	Uncharacterized protein C17orf62 OS=Homo sapiens GN=C17orf62 PE=1 SV=1 - [CQ062_HUMAN]
P27708	CAD	CAD protein OS=Homo sapiens GN=CAD PE=1 SV=3 - [PYR1_HUMAN]
P62158	CALM1	Calmodulin OS=Homo sapiens GN=CALM1 PE=1 SV=2 - [CALM_HUMAN]
O43852	CALU	Calumenin OS=Homo sapiens GN=CALU PE=1 SV=2 - [CALU_HUMAN]

P78371	CCT2	T-complex protein 1 subunit beta OS=Homo sapiens GN=CCT2 PE=1 SV=4 - [TCPB_HUMAN]
P49368	CCT3	T-complex protein 1 subunit gamma OS=Homo sapiens GN=CCT3 PE=1 SV=4 - [TCPG_HUMAN]
P48643	CCT5	T-complex protein 1 subunit epsilon OS=Homo sapiens GN=CCT5 PE=1 SV=1 - [TCPE_HUMAN]
P40227	CCT6A	T-complex protein 1 subunit zeta OS=Homo sapiens GN=CCT6A PE=1 SV=3 - [TCPZ_HUMAN]
Q99832	CCT7	T-complex protein 1 subunit eta OS=Homo sapiens GN=CCT7 PE=1 SV=2 - [TCPH_HUMAN]
P50990	CCT8	T-complex protein 1 subunit theta OS=Homo sapiens GN=CCT8 PE=1 SV=4 - [TCPQ_HUMAN]
P06493	CDK1	Cyclin-dependent kinase 1 OS=Homo sapiens GN=CDK1 PE=1 SV=3 - [CDK1_HUMAN]
P23528	CFL1	Cofilin-1 OS=Homo sapiens GN=CFL1 PE=1 SV=3 - [COF1_HUMAN]
Q9NX63	CHCHD3	Coiled-coil-helix-coiled-coil-helix domain-containing protein 3, mitochondrial OS=Homo sapiens GN=CHCHD3 PE=1 SV=1 - [CHCH3_HUMAN]
P10909	CLU	Clusterin OS=Homo sapiens GN=CLU PE=1 SV=1 - [CLUS_HUMAN]
P53618	COPB1	Coatomer subunit beta OS=Homo sapiens GN=COPB1 PE=1 SV=3 - [COPB_HUMAN]
P35606	COPB2	Coatomer subunit beta' OS=Homo sapiens GN=COPB2 PE=1 SV=2 - [COPB2_HUMAN]
Q9Y678	COPG1	Coatomer subunit gamma-1 OS=Homo sapiens GN=COPG1 PE=1 SV=1 - [COPG1_HUMAN]
Q9UBF2	COPG2	Coatomer subunit gamma-2 OS=Homo sapiens GN=COPG2 PE=1 SV=1 - [COPG2_HUMAN]
P50416	CPT1A	Carnitine O-palmitoyltransferase 1, liver isoform OS=Homo sapiens GN=CPT1A PE=1 SV=2 - [CPT1A_HUMAN]
P08574	CYC1	Cytochrome c1, heme protein, mitochondrial OS=Homo sapiens GN=CYC1 PE=1 SV=3 - [CY1_HUMAN]
P31689	DNAJA1	DnaJ homolog subfamily A member 1 OS=Homo sapiens GN=DNAJA1 PE=1 SV=2 - [DNJA1_HUMAN]
O60884	DNAJA2	DnaJ homolog subfamily A member 2 OS=Homo sapiens GN=DNAJA2 PE=1 SV=1 - [DNJA2_HUMAN]
Q02413	DSG1	Desmoglein-1 OS=Homo sapiens GN=DSG1 PE=1 SV=2 - [DSG1_HUMAN]
Q13011	ECH1	Delta(3,5)-Delta(2,4)-dienoyl-CoA isomerase, mitochondrial OS=Homo sapiens GN=ECH1 PE=1 SV=2 - [ECH1_HUMAN]
Q9H223	EHD4	EH domain-containing protein 4 OS=Homo sapiens GN=EHD4 PE=1 SV=1 - [EHD4_HUMAN]
Q969X5	ERGIC1	Endoplasmic reticulum-Golgi intermediate compartment protein 1 OS=Homo sapiens GN=ERGIC1 PE=1 SV=1 - [ERGI1_HUMAN]
O75477	ERLIN1	Erlin-1 OS=Homo sapiens GN=ERLIN1 PE=1 SV=1 - [ERLN1_HUMAN]
Q96CS3	FAF2	FAS-associated factor 2 OS=Homo sapiens GN=FAF2 PE=1 SV=2 - [FAF2_HUMAN]
Q96GK7	FAHD2A	Fumarylacetoacetate hydrolase domain-containing protein 2A OS=Homo sapiens GN=FAHD2A PE=1 SV=1 - [FAH2A_HUMAN]
Q96KR6	FAM210B	Protein FAM210B OS=Homo sapiens GN=FAM210B PE=1 SV=2 - [F210B_HUMAN]
O75369	FLNB	Filamin-B OS=Homo sapiens GN=FLNB PE=1 SV=2 - [FLNB_HUMAN]
P62873	GNB1	Guanine nucleotide-binding protein G(I)/G(S)/G(T) subunit beta-1 OS=Homo sapiens GN=GNB1 PE=1 SV=3 - [GBB1_HUMAN]
Q9UBI6	GNG12	Guanine nucleotide-binding protein G(I)/G(S)/G(O) subunit gamma-12 OS=Homo sapiens GN=GNG12 PE=1 SV=3 - [GBG12_HUMAN]
O43824	GTPBP6	Putative GTP-binding protein 6 OS=Homo sapiens GN=GTPBP6 PE=2 SV=3 - [GTPB6_HUMAN]
P0C0S5	H2AFZ	Histone H2A.Z OS=Homo sapiens GN=H2AFZ PE=1 SV=2 - [H2AZ_HUMAN]
O60814	HIST1H2BK	Histone H2B type 1-K OS=Homo sapiens GN=HIST1H2BK PE=1 SV=3 -

		[H2B1K_HUMAN]
P62805	HIST1H4A	Histone H4 OS=Homo sapiens GN=HIST1H4A PE=1 SV=2 - [H4_HUMAN]
Q53GQ0	HSD17B12	Estradiol 17-beta-dehydrogenase 12 OS=Homo sapiens GN=HSD17B12 PE=1 SV=2 - [DHB12_HUMAN]
Q6YN16	HSDL2	Hydroxysteroid dehydrogenase-like protein 2 OS=Homo sapiens GN=HSDL2 PE=1 SV=1 - [HSDL2_HUMAN]
P08107	HSPA1A	Heat shock 70 kDa protein 1A/1B OS=Homo sapiens GN=HSPA1A PE=1 SV=5 - [HSP71_HUMAN]
P38646	HSPA9	Stress-70 protein, mitochondrial OS=Homo sapiens GN=HSPA9 PE=1 SV=2 - [GRP75_HUMAN]
P04792	HSPB1	Heat shock protein beta-1 OS=Homo sapiens GN=HSPB1 PE=1 SV=2 - [HSPB1_HUMAN]
Q9BUP3	HTATIP2	Oxidoreductase HTATIP2 OS=Homo sapiens GN=HTATIP2 PE=1 SV=2 - [HTAI2_HUMAN]
Q96D96	HVCN1	Voltage-gated hydrogen channel 1 OS=Homo sapiens GN=HVCN1 PE=1 SV=1 - [HVCN1_HUMAN]
P46940	IQGAP1	Ras GTPase-activating-like protein IQGAP1 OS=Homo sapiens GN=IQGAP1 PE=1 SV=1 - [IQGA1_HUMAN]
Q14974	KPNB1	Importin subunit beta-1 OS=Homo sapiens GN=KPNB1 PE=1 SV=2 - [IMB1_HUMAN]
P07195	LDHB	L-lactate dehydrogenase B chain OS=Homo sapiens GN=LDHB PE=1 SV=2 - [LDHB_HUMAN]
O95202	LETM1	LETM1 and EF-hand domain-containing protein 1, mitochondrial OS=Homo sapiens GN=LETM1 PE=1 SV=1 - [LETM1_HUMAN]
Q08380	LGALS3BP	Galectin-3-binding protein OS=Homo sapiens GN=LGALS3BP PE=1 SV=1 - [LG3BP_HUMAN]
P49257	LMAN1	Protein ERGIC-53 OS=Homo sapiens GN=LMAN1 PE=1 SV=2 - [LMAN1_HUMAN]
Q12907	LMAN2	Vesicular integral-membrane protein VIP36 OS=Homo sapiens GN=LMAN2 PE=1 SV=1 - [LMAN2_HUMAN]
Q96AG4	LRRC59	Leucine-rich repeat-containing protein 59 OS=Homo sapiens GN=LRRC59 PE=1 SV=1 - [LRC59_HUMAN]
Q96RQ3	MCCC1	Methylcrotonoyl-CoA carboxylase subunit alpha, mitochondrial OS=Homo sapiens GN=MCCC1 PE=1 SV=3 - [MCCA_HUMAN]
P33993	MCM7	DNA replication licensing factor MCM7 OS=Homo sapiens GN=MCM7 PE=1 SV=4 - [MCM7_HUMAN]
P60660	MYL6	Myosin light polypeptide 6 OS=Homo sapiens GN=MYL6 PE=1 SV=2 - [MYL6_HUMAN]
Q9NZM1	MYOF	Myoferlin OS=Homo sapiens GN=MYOF PE=1 SV=1 - [MYOF_HUMAN]
P43490	NAMPT	Nicotinamide phosphoribosyltransferase OS=Homo sapiens GN=NAMPT PE=1 SV=1 - [NAMPT_HUMAN]
O00483	NDUFA4	NADH dehydrogenase [ubiquinone] 1 alpha subcomplex subunit 4 OS=Homo sapiens GN=NDUFA4 PE=1 SV=1 - [NDUA4_HUMAN]
P28331	NDUFS1	NADH-ubiquinone oxidoreductase 75 kDa subunit, mitochondrial OS=Homo sapiens GN=NDUFS1 PE=1 SV=3 - [NDUS1_HUMAN]
O75306	NDUFS2	NADH dehydrogenase [ubiquinone] iron-sulfur protein 2, mitochondrial OS=Homo sapiens GN=NDUFS2 PE=1 SV=2 - [NDUS2_HUMAN]
O75251	NDUFS7	NADH dehydrogenase [ubiquinone] iron-sulfur protein 7, mitochondrial OS=Homo sapiens GN=NDUFS7 PE=1 SV=3 - [NDUS7_HUMAN]
Q13423	NNT	NAD(P) transhydrogenase, mitochondrial OS=Homo sapiens GN=NNT PE=1 SV=3 - [NNTM_HUMAN]
Q9NX40	OCIAD1	OCIA domain-containing protein 1 OS=Homo sapiens GN=OCIAD1 PE=1 SV=1 - [OCAD1_HUMAN]
O60313	OPA1	Dynamin-like 120 kDa protein, mitochondrial OS=Homo sapiens GN=OPA1 PE=1 SV=3 - [OPA1_HUMAN]
P13674	P4HA1	Prolyl 4-hydroxylase subunit alpha-1 OS=Homo sapiens GN=P4HA1 PE=1 SV=2 - [P4HA1_HUMAN]
P05166	PCCB	Propionyl-CoA carboxylase beta chain, mitochondrial OS=Homo sapiens

		GN=PCCB PE=1 SV=3 - [PCCB_HUMAN]
P12004	PCNA	Proliferating cell nuclear antigen OS=Homo sapiens GN=PCNA PE=1 SV=1 - [PCNA_HUMAN]
O95394	PGM3	Phosphoacetylglucosamine mutase OS=Homo sapiens GN=PGM3 PE=1 SV=1 - [AGM1_HUMAN]
O15173	PGRMC2	Membrane-associated progesterone receptor component 2 OS=Homo sapiens GN=PGRMC2 PE=1 SV=1 - [PGRC2_HUMAN]
Q99623	PHB2	Prohibitin-2 OS=Homo sapiens GN=PHB2 PE=1 SV=2 - [PHB2_HUMAN]
P30041	PRDX6	Peroxiredoxin-6 OS=Homo sapiens GN=PRDX6 PE=1 SV=3 - [PRDX6_HUMAN]
P78527	PRKDC	DNA-dependent protein kinase catalytic subunit OS=Homo sapiens GN=PRKDC PE=1 SV=3 - [PRKDC_HUMAN]
P62195	PSMC5	26S protease regulatory subunit 8 OS=Homo sapiens GN=PSMC5 PE=1 SV=1 - [PRS8_HUMAN]
Q99460	PSMD1	26S proteasome non-ATPase regulatory subunit 1 OS=Homo sapiens GN=PSMD1 PE=1 SV=2 - [PSMD1_HUMAN]
Q06323	PSME1	Proteasome activator complex subunit 1 OS=Homo sapiens GN=PSME1 PE=1 SV=1 - [PSME1_HUMAN]
P06454	PTMA	Prothymosin alpha OS=Homo sapiens GN=PTMA PE=1 SV=2 - [PTMA_HUMAN]
P32322	PYCR1	Pyrroline-5-carboxylate reductase 1, mitochondrial OS=Homo sapiens GN=PYCR1 PE=1 SV=2 - [P5CR1_HUMAN]
Q96C36	PYCR2	Pyrroline-5-carboxylate reductase 2 OS=Homo sapiens GN=PYCR2 PE=1 SV=1 - [P5CR2_HUMAN]
P11216	PYGB	Glycogen phosphorylase, brain form OS=Homo sapiens GN=PYGB PE=1 SV=5 - [PYGB_HUMAN]
P61026	RAB10	Ras-related protein Rab-10 OS=Homo sapiens GN=RAB10 PE=1 SV=1 - [RAB10_HUMAN]
P62491	RAB11A	Ras-related protein Rab-11A OS=Homo sapiens GN=RAB11A PE=1 SV=3 - [RB11A_HUMAN]
P51153	RAB13	Ras-related protein Rab-13 OS=Homo sapiens GN=RAB13 PE=1 SV=1 - [RAB13_HUMAN]
Q9NP72	RAB18	Ras-related protein Rab-18 OS=Homo sapiens GN=RAB18 PE=1 SV=1 - [RAB18_HUMAN]
Q15286	RAB35	Ras-related protein Rab-35 OS=Homo sapiens GN=RAB35 PE=1 SV=1 - [RAB35_HUMAN]
P51148	RAB5C	Ras-related protein Rab-5C OS=Homo sapiens GN=RAB5C PE=1 SV=2 - [RAB5C_HUMAN]
P20340	RAB6A	Ras-related protein Rab-6A OS=Homo sapiens GN=RAB6A PE=1 SV=3 - [RAB6A_HUMAN]
P51149	RAB7A	Ras-related protein Rab-7a OS=Homo sapiens GN=RAB7A PE=1 SV=1 - [RAB7A_HUMAN]
Q92878	RAD50	DNA repair protein RAD50 OS=Homo sapiens GN=RAD50 PE=1 SV=1 - [RAD50_HUMAN]
P11233	RALA	Ras-related protein Ral-A OS=Homo sapiens GN=RALA PE=1 SV=1 - [RALA_HUMAN]
P61224	RAP1B	Ras-related protein Rap-1b OS=Homo sapiens GN=RAP1B PE=1 SV=1 - [RAP1B_HUMAN]
Q15293	RCN1	Reticulocalbin-1 OS=Homo sapiens GN=RCN1 PE=1 SV=1 - [RCN1_HUMAN]
Q9HBH5	RDH14	Retinol dehydrogenase 14 OS=Homo sapiens GN=RDH14 PE=1 SV=1 - [RDH14_HUMAN]
Q15382	RHEB	GTP-binding protein Rheb OS=Homo sapiens GN=RHEB PE=1 SV=1 - [RHEB_HUMAN]
Q63HN8	RNF213	E3 ubiquitin-protein ligase RNF213 OS=Homo sapiens GN=RNF213 PE=1 SV=3 - [RN213_HUMAN]
Q16799	RTN1	Reticulon-1 OS=Homo sapiens GN=RTN1 PE=1 SV=1 - [RTN1_HUMAN]
Q9NQC3	RTN4	Reticulon-4 OS=Homo sapiens GN=RTN4 PE=1 SV=2 - [RTN4_HUMAN]

Q9Y230	RUVBL2	RuvB-like 2 OS=Homo sapiens GN=RUVBL2 PE=1 SV=3 - [RUVB2_HUMAN]
Q9Y6B6	SAR1B	GTP-binding protein SAR1b OS=Homo sapiens GN=SAR1B PE=1 SV=1 - [SAR1B_HUMAN]
Q6UWP8	SBSN	Suprabasin OS=Homo sapiens GN=SBSN PE=2 SV=2 - [SBSN_HUMAN]
O14828	SCAMP3	Secretory carrier-associated membrane protein 3 OS=Homo sapiens GN=SCAMP3 PE=1 SV=3 - [SCAM3_HUMAN]
Q8NBX0	SCCPDH	Saccharopine dehydrogenase-like oxidoreductase OS=Homo sapiens GN=SCCPDH PE=1 SV=1 - [SCPDL_HUMAN]
P31040	SDHA	Succinate dehydrogenase [ubiquinone] flavoprotein subunit, mitochondrial OS=Homo sapiens GN=SDHA PE=1 SV=2 - [DHSA_HUMAN]
P21912	SDHB	Succinate dehydrogenase [ubiquinone] iron-sulfur subunit, mitochondrial OS=Homo sapiens GN=SDHB PE=1 SV=3 - [DHSB_HUMAN]
P61619	SEC61A1	Protein transport protein Sec61 subunit alpha isoform 1 OS=Homo sapiens GN=SEC61A1 PE=1 SV=2 - [S61A1_HUMAN]
P60468	SEC61B	Protein transport protein Sec61 subunit beta OS=Homo sapiens GN=SEC61B PE=1 SV=2 - [SC61B_HUMAN]
Q9BWM7	SFXN3	Sideroflexin-3 OS=Homo sapiens GN=SFXN3 PE=1 SV=2 - [SFXN3_HUMAN]
P53007	SLC25A1	Tricarboxylate transport protein, mitochondrial OS=Homo sapiens GN=SLC25A1 PE=1 SV=2 - [TXTP_HUMAN]
Q9UBX3	SLC25A10	Mitochondrial dicarboxylate carrier OS=Homo sapiens GN=SLC25A10 PE=1 SV=2 - [DIC_HUMAN]
Q08AF3	SLFN5	Schlafen family member 5 OS=Homo sapiens GN=SLFN5 PE=1 SV=1 - [SLFN5_HUMAN]
Q9Y6N5	SQRDL	Sulfide:quinone oxidoreductase, mitochondrial OS=Homo sapiens GN=SQRDL PE=1 SV=1 - [SQRD_HUMAN]
P17987	TCP1	T-complex protein 1 subunit alpha OS=Homo sapiens GN=TCP1 PE=1 SV=1 - [TCPA_HUMAN]
Q9UM00	TMCO1	Transmembrane and coiled-coil domain-containing protein 1 OS=Homo sapiens GN=TMCO1 PE=1 SV=1 - [TMCO1_HUMAN]
P49755	TMED10	Transmembrane emp24 domain-containing protein 10 OS=Homo sapiens GN=TMED10 PE=1 SV=2 - [TMEDA_HUMAN]
Q15363	TMED2	Transmembrane emp24 domain-containing protein 2 OS=Homo sapiens GN=TMED2 PE=1 SV=1 - [TMED2_HUMAN]
Q7Z7H5	TMED4	Transmembrane emp24 domain-containing protein 4 OS=Homo sapiens GN=TMED4 PE=1 SV=1 - [TMED4_HUMAN]
Q9Y3B3	TMED7	Transmembrane emp24 domain-containing protein 7 OS=Homo sapiens GN=TMED7 PE=1 SV=2 - [TMED7_HUMAN]
P57088	TMEM33	Transmembrane protein 33 OS=Homo sapiens GN=TMEM33 PE=1 SV=2 - [TMM33_HUMAN]
Q5JTV8	TOR1AIP1	Torsin-1A-interacting protein 1 OS=Homo sapiens GN=TOR1AIP1 PE=1 SV=2 - [TOIP1_HUMAN]
P60174	TPI1	Triosephosphate isomerase OS=Homo sapiens GN=TPI1 PE=1 SV=3 - [TPIS_HUMAN]
Q12931	TRAP1	Heat shock protein 75 kDa, mitochondrial OS=Homo sapiens GN=TRAP1 PE=1 SV=3 - [TRAP1_HUMAN]
Q13263	TRIM28	Transcription intermediary factor 1-beta OS=Homo sapiens GN=TRIM28 PE=1 SV=5 - [TIF1B_HUMAN]
P10599	TXN	Thioredoxin OS=Homo sapiens GN=TXN PE=1 SV=3 - [THIO_HUMAN]
P11441	UBL4A	Ubiquitin-like protein 4A OS=Homo sapiens GN=UBL4A PE=1 SV=1 - [UBL4A_HUMAN]
P22695	UQCRC2	Cytochrome b-c1 complex subunit 2, mitochondrial OS=Homo sapiens GN=UQCRC2 PE=1 SV=3 - [QCR2_HUMAN]
Q9P0L0	VAPA	Vesicle-associated membrane protein-associated protein A OS=Homo sapiens GN=VAPA PE=1 SV=3 - [VAPA_HUMAN]
O95292	VAPB	Vesicle-associated membrane protein-associated protein B/C OS=Homo sapiens GN=VAPB PE=1 SV=3 - [VAPB_HUMAN]
Q9Y277	VDAC3	Voltage-dependent anion-selective channel protein 3 OS=Homo sapiens GN=VDAC3 PE=1 SV=1 - [VDAC3_HUMAN]

A3KMH1	VWA8	von Willebrand factor A domain-containing protein 8 OS=Homo sapiens GN=VWA8 PE=1 SV=2 - [VWA8_HUMAN]
P31946	YWHAB	14-3-3 protein beta/alpha OS=Homo sapiens GN=YWHAB PE=1 SV=3 - [1433B_HUMAN]
**Proteins identified in this assay, but in common with proteins in the negative control**		
O95340	PAPSS2	Bifunctional 3'-phosphoadenosine 5'-phosphosulfate synthase 2 OS=Homo sapiens GN=PAPSS2 PE=1 SV=2 - [PAPS2_HUMAN]
O95573	ACSL3	Long-chain-fatty-acid--CoA ligase 3 OS=Homo sapiens GN=ACSL3 PE=1 SV=3 - [ACSL3_HUMAN]
P00966	ASS1	Argininosuccinate synthase OS=Homo sapiens GN=ASS1 PE=1 SV=2 - [ASSY_HUMAN]
P04075	ALDOA	Fructose-bisphosphate aldolase A OS=Homo sapiens GN=ALDOA PE=1 SV=2 - [ALDOA_HUMAN]
P04406	GAPDH	Glyceraldehyde-3-phosphate dehydrogenase OS=Homo sapiens GN=GAPDH PE=1 SV=3 - [G3P_HUMAN]
P04439	HLA-A	HLA class I histocompatibility antigen, A-3 alpha chain OS=Homo sapiens GN=HLA-A PE=1 SV=2 - [1A03_HUMAN]
P04843	RPN1	Dolichyl-diphosphooligosaccharide--protein glycosyltransferase subunit 1 OS=Homo sapiens GN=RPN1 PE=1 SV=1 - [RPN1_HUMAN]
P04844	RPN2	Dolichyl-diphosphooligosaccharide--protein glycosyltransferase subunit 2 OS=Homo sapiens GN=RPN2 PE=1 SV=3 - [RPN2_HUMAN]
P05141	SLC25A5	ADP/ATP translocase 2 OS=Homo sapiens GN=SLC25A5 PE=1 SV=7 - [ADT2_HUMAN]
P07099	EPHX1	Epoxide hydrolase 1 OS=Homo sapiens GN=EPHX1 PE=1 SV=1 - [HYEP_HUMAN]
P07237	P4HB	Protein disulfide-isomerase OS=Homo sapiens GN=P4HB PE=1 SV=3 - [PDIA1_HUMAN]
P08240	SRPR	Signal recognition particle receptor subunit alpha OS=Homo sapiens GN=SRPR PE=1 SV=2 - [SRPR_HUMAN]
P08670	VIM	Vimentin OS=Homo sapiens GN=VIM PE=1 SV=4 - [VIME_HUMAN]
P09622	DLD	Dihydrolipoyl dehydrogenase, mitochondrial OS=Homo sapiens GN=DLD PE=1 SV=2 - [DLDH_HUMAN]
P10155	TROVE2	60 kDa SS-A/Ro ribonucleoprotein OS=Homo sapiens GN=TROVE2 PE=1 SV=2 - [RO60_HUMAN]
P10809	HSPD1	60 kDa heat shock protein, mitochondrial OS=Homo sapiens GN=HSPD1 PE=1 SV=2 - [CH60_HUMAN]
P11172	UMPS	Uridine 5'-monophosphate synthase OS=Homo sapiens GN=UMPS PE=1 SV=1 - [UMPS_HUMAN]
P11586	MTHFD1	C-1-tetrahydrofolate synthase, cytoplasmic OS=Homo sapiens GN=MTHFD1 PE=1 SV=3 - [C1TC_HUMAN]
P12236	SLC25A6	ADP/ATP translocase 3 OS=Homo sapiens GN=SLC25A6 PE=1 SV=4 - [ADT3_HUMAN]
P14625	HSP90B1	Endoplasmin OS=Homo sapiens GN=HSP90B1 PE=1 SV=1 - [ENPL_HUMAN]
P14923	JUP	Junction plakoglobin OS=Homo sapiens GN=JUP PE=1 SV=3 - [PLAK_HUMAN]
P15559	NQO1	NAD(P)H dehydrogenase [quinone] 1 OS=Homo sapiens GN=NQO1 PE=1 SV=1 - [NQO1_HUMAN]
P15924	DSP	Desmoplakin OS=Homo sapiens GN=DSP PE=1 SV=3 - [DESP_HUMAN]
P17931	LGALS3	Galectin-3 OS=Homo sapiens GN=LGALS3 PE=1 SV=5 - [LEG3_HUMAN]
P18124	RPL7	60S ribosomal protein L7 OS=Homo sapiens GN=RPL7 PE=1 SV=1 - [RL7_HUMAN]
P20700	LMNB1	Lamin-B1 OS=Homo sapiens GN=LMNB1 PE=1 SV=2 - [LMNB1_HUMAN]
P23396	RPS3	40S ribosomal protein S3 OS=Homo sapiens GN=RPS3 PE=1 SV=2 - [RS3_HUMAN]
P25705	ATP5A1	ATP synthase subunit alpha, mitochondrial OS=Homo sapiens GN=ATP5A1 PE=1 SV=1 - [ATPA_HUMAN]
P27824	CANX	Calnexin OS=Homo sapiens GN=CANX PE=1 SV=2 - [CALX_HUMAN]

P31327	CPS1	Carbamoyl-phosphate synthase [ammonia], mitochondrial OS=Homo sapiens GN=CPS1 PE=1 SV=2 - [CPSM_HUMAN]
P35232	PHB	Prohibitin OS=Homo sapiens GN=PHB PE=1 SV=1 - [PHB_HUMAN]
P38117	ETFB	Electron transfer flavoprotein subunit beta OS=Homo sapiens GN=ETFB PE=1 SV=3 - [ETFB_HUMAN]
P39656	DDOST	Dolichyl-diphosphooligosaccharide--protein glycosyltransferase 48 kDa subunit OS=Homo sapiens GN=DDOST PE=1 SV=4 - [OST48_HUMAN]
P46379	BAG6	Large proline-rich protein BAG6 OS=Homo sapiens GN=BAG6 PE=1 SV=2 - [BAG6_HUMAN]
P50991	CCT4	T-complex protein 1 subunit delta OS=Homo sapiens GN=CCT4 PE=1 SV=4 - [TCPD_HUMAN]
P53621	COPA	Coatomer subunit alpha OS=Homo sapiens GN=COPA PE=1 SV=2 - [COPA_HUMAN]
P61106	RAB14	Ras-related protein Rab-14 OS=Homo sapiens GN=RAB14 PE=1 SV=4 - [RAB14_HUMAN]
P61247	RPS3A	40S ribosomal protein S3a OS=Homo sapiens GN=RPS3A PE=1 SV=2 - [RS3A_HUMAN]
P62263	RPS14	40S ribosomal protein S14 OS=Homo sapiens GN=RPS14 PE=1 SV=3 - [RS14_HUMAN]
P62269	RPS18	40S ribosomal protein S18 OS=Homo sapiens GN=RPS18 PE=1 SV=3 - [RS18_HUMAN]
P62424	RPL7A	60S ribosomal protein L7a OS=Homo sapiens GN=RPL7A PE=1 SV=2 - [RL7A_HUMAN]
P62701	RPS4X	40S ribosomal protein S4, X isoform OS=Homo sapiens GN=RPS4X PE=1 SV=2 - [RS4X_HUMAN]
P62820	RAB1A	Ras-related protein Rab-1A OS=Homo sapiens GN=RAB1A PE=1 SV=3 - [RAB1A_HUMAN]
P63244	GNB2L1	Guanine nucleotide-binding protein subunit beta-2-like 1 OS=Homo sapiens GN=GNB2L1 PE=1 SV=3 - [GBLP_HUMAN]
P67936	TPM4	Tropomyosin alpha-4 chain OS=Homo sapiens GN=TPM4 PE=1 SV=3 - [TPM4_HUMAN]
P68371	TUBB4B	Tubulin beta-4B chain OS=Homo sapiens GN=TUBB4B PE=1 SV=1 - [TBB4B_HUMAN]
Q00325	SLC25A3	Phosphate carrier protein, mitochondrial OS=Homo sapiens GN=SLC25A3 PE=1 SV=2 - [MPCP_HUMAN]
Q02218	OGDH	2-oxoglutarate dehydrogenase, mitochondrial OS=Homo sapiens GN=OGDH PE=1 SV=3 - [ODO1_HUMAN]
Q06830	PRDX1	Peroxiredoxin-1 OS=Homo sapiens GN=PRDX1 PE=1 SV=1 - [PRDX1_HUMAN]
Q13085	ACACA	Acetyl-CoA carboxylase 1 OS=Homo sapiens GN=ACACA PE=1 SV=2 - [ACACA_HUMAN]
Q13409	DYNC1I2	Cytoplasmic dynein 1 intermediate chain 2 OS=Homo sapiens GN=DYNC1I2 PE=1 SV=3 - [DC1I2_HUMAN]
Q15233	NONO	Non-POU domain-containing octamer-binding protein OS=Homo sapiens GN=NONO PE=1 SV=4 - [NONO_HUMAN]
Q86YZ3	HRNR	Hornerin OS=Homo sapiens GN=HRNR PE=1 SV=2 - [HORN_HUMAN]
Q92604	LPGAT1	Acyl-CoA:lysophosphatidylglycerol acyltransferase 1 OS=Homo sapiens GN=LPGAT1 PE=2 SV=1 - [LGAT1_HUMAN]
Q96C19	EFHD2	EF-hand domain-containing protein D2 OS=Homo sapiens GN=EFHD2 PE=1 SV=1 - [EFHD2_HUMAN]
Q9H9B4	SFXN1	Sideroflexin-1 OS=Homo sapiens GN=SFXN1 PE=1 SV=4 - [SFXN1_HUMAN]
