# Supplementary material for: Identification of RNA-binding proteins in exosomes capable of interacting with different types of RNA: RBP-facilitated transport of RNAs into exosomes
Source: PLoS One. 2018 Apr 24;13(4):e0195969. doi: 10.1371/journal.pone.0195969 (PMC5918169; doi:10.1371/journal.pone.0195969)
Supplement: S7 Table — The RQ values have been calculated accordingly to: (1) endogenous control GAPDH and (2) calibrator sample negative control (HTB-177 cells treated with NC-siRNA). Data from four biological replicates are shown. (PDF) [file pone.0195969.s013.pdf]

S7 Table. MVP gene qPCR dataset after its silencing in HTB cells. The RQ values have been calculated accordingly to: (1) endogenous control GAPDH and (2) calibrator sample negative control (HTB-177 cells treated with NC-siRNA). Data from four biological replicates are shown.

**REPLICATE 1**					
**Sample Name**	**Gene**	**Ct**			
MVP-siRNA treated cells - 24h	GAPDH	15,00			
NC-siRNA treated cells - 24h	GAPDH	15,54			
**MVP siRNA treated cells - 48h**	**GAPDH**	**14,61**			
**NC-siRNA treated cells - 48h**	**GAPDH**	**15,43**			
MVP siRNA treated cells - 72h	GAPDH	15,30			
NC-siRNA treated cells - 72h	GAPDH	15,39			
**Sample Name**	**Gene**	**Ct**	**Delta Ct**	**Delta-delta Ct**	**RQ**
MVP-siRNA treated cells - 24h	MVP	27,89	12,89	2,87	0,14
NC-siRNA treated cells - 24h	MVP	25,56	10,02		
**MVP siRNA treated cells - 48h**	**MVP**	**27,55**	**12,93**	**2,96**	**0,13**
**NC-siRNA treated cells - 48h**	**MVP**	**25,40**	**9,97**		
MVP siRNA treated cells - 72h	MVP	27,56	12,27	2,52	0,17
NC-siRNA treated cells - 72h	MVP	25,14	9,74		

**REPLICATE 2**					
**Sample Name**	**Gene**	**Ct**			
MVP-siRNA treated cells - 24h	GAPDH	16,68			
NC-siRNA treated cells - 24h	GAPDH	17,23			
**MVP siRNA treated cells - 48h**	**GAPDH**	**16,56**			
**NC-siRNA treated cells - 48h**	**GAPDH**	**16,87**			
MVP siRNA treated cells - 72h	GAPDH	16,47			
NC-siRNA treated cells - 72h	GAPDH	16,86			
**Sample Name**	**Gene**	**Ct**	**Delta Ct**	**Delta-delta Ct**	**RQ**
MVP-siRNA treated cells - 24h	MVP	29,10	12,42	2,74	0,15
NC-siRNA treated cells - 24h	MVP	26,90	9,67		
**MVP siRNA treated cells - 48h**	**MVP**	**30,30**	**13,73**	**3,12**	**0,11**
**NC-siRNA treated cells - 48h**	**MVP**	**27,47**	**10,61**		
MVP siRNA treated cells - 72h	MVP	29,61	13,14	2,57	0,17
NC-siRNA treated cells - 72h	MVP	27,43	10,57		

REPLICATE 3					
**Sample Name**	**Gene**	**Ct**			
MVP-siRNA treated cells - 24h	GAPDH	16,19			
NC-siRNA treated cells - 24h	GAPDH	16,03			
**MVP siRNA treated cells - 48h**	**GAPDH**	**15,82**			
**NC-siRNA treated cells - 48h**	**GAPDH**	**16,06**			
MVP siRNA treated cells - 72h	GAPDH	15,53			
NC-siRNA treated cells - 72h	GAPDH	15,66			
**Sample Name**	**Gene**	**Ct**	**Delta Ct**	**Delta-delta Ct**	**RQ**
MVP-siRNA treated cells - 24h	MVP	29,47	13,28	2,16	0,22
NC-siRNA treated cells - 24h	MVP	27,14	11,12		
**MVP siRNA treated cells - 48h**	**MVP**	**29,31**	**13,49**	**1,93**	**0,26**
**NC-siRNA treated cells - 48h**	**MVP**	**27,62**	**11,57**		
MVP siRNA treated cells - 72h	MVP	27,63	12,10	1,73	0,30
NC-siRNA treated cells - 72h	MVP	26,02	10,36		

REPLICATE 4					
**Sample Name**	**Gene**	**Ct**			
MVP-siRNA treated cells - 24h	GAPDH	15,95			
NC-siRNA treated cells - 24h	GAPDH	15,97			
**MVP siRNA treated cells - 48h**	**GAPDH**	**15,93**			
**NC-siRNA treated cells - 48h**	**GAPDH**	**15,93**			
MVP siRNA treated cells - 72h	GAPDH	15,68			
NC-siRNA treated cells - 72h	GAPDH	15,30			
**Sample Name**	**Gene**	**Ct**	**Delta Ct**	**Delta-delta Ct**	**RQ**
MVP-siRNA treated cells - 24h	MVP	28,85	12,90	1,59	0,33
NC-siRNA treated cells - 24h	MVP	27,28	11,31		
**MVP siRNA treated cells - 48h**	**MVP**	**28,99**	**13,06**	**1,35**	**0,39**
**NC-siRNA treated cells - 48h**	**MVP**	**27,64**	**11,71**		
MVP siRNA treated cells - 72h	MVP	27,55	11,88	1,47	0,36
NC-siRNA treated cells - 72h	MVP	25,70	10,41		
